# Supplementary material for: Humans homozygous for rare or common hypomorphic IL23R variants are prone to tuberculosis
Source: J Exp Med. 2026 Jul 9;223(8):e20252236. doi: 10.1084/jem.20252236 (PMC13348821; doi:10.1084/jem.20252236)
Supplement: SourceData F1 — is the source file for Fig. 1. [file jem_20252236_sourcedataf1.pdf]

SourceDataF1

B

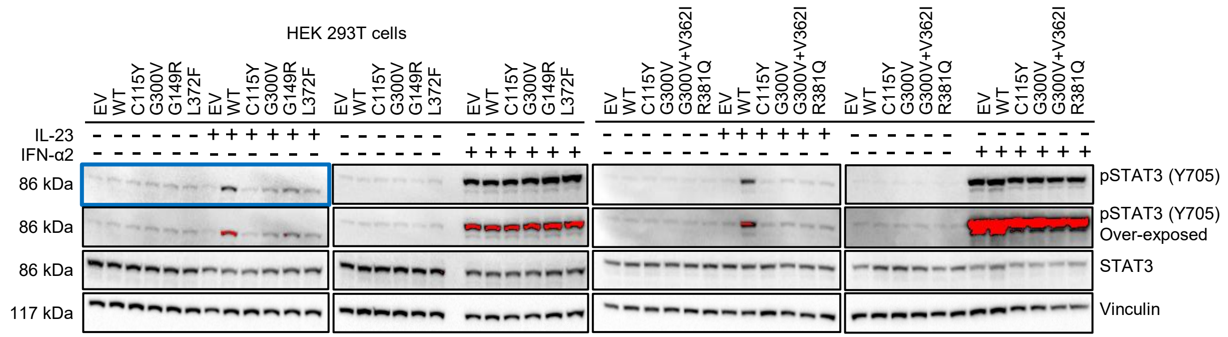

Showed

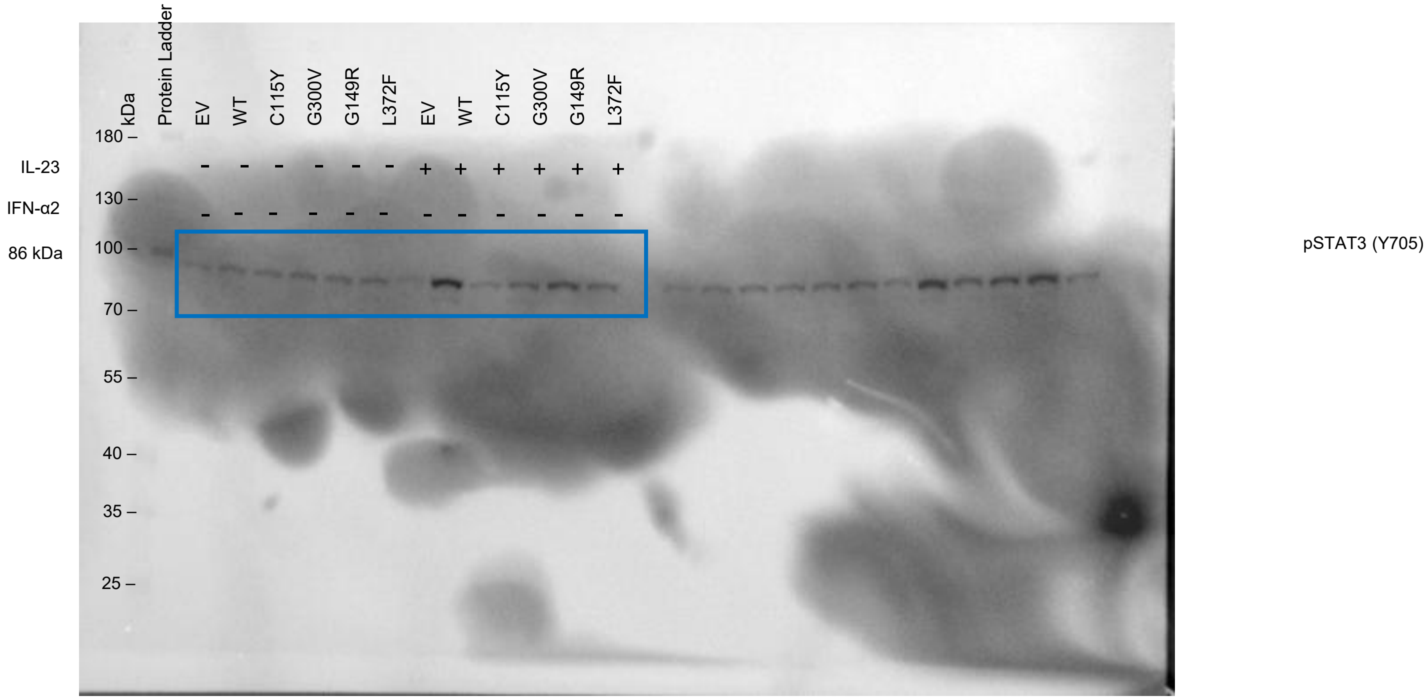

The ladder image was merged with the original image from the figure to visualize protein size

SourceDataF1

B

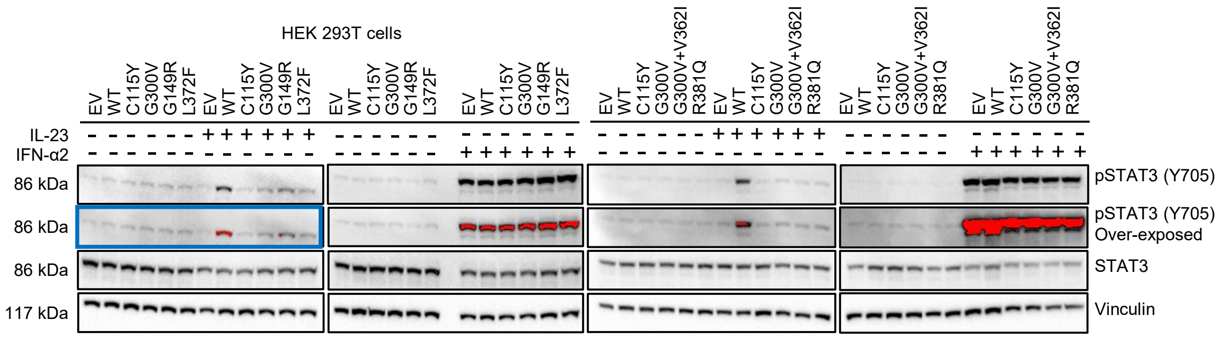

Showed

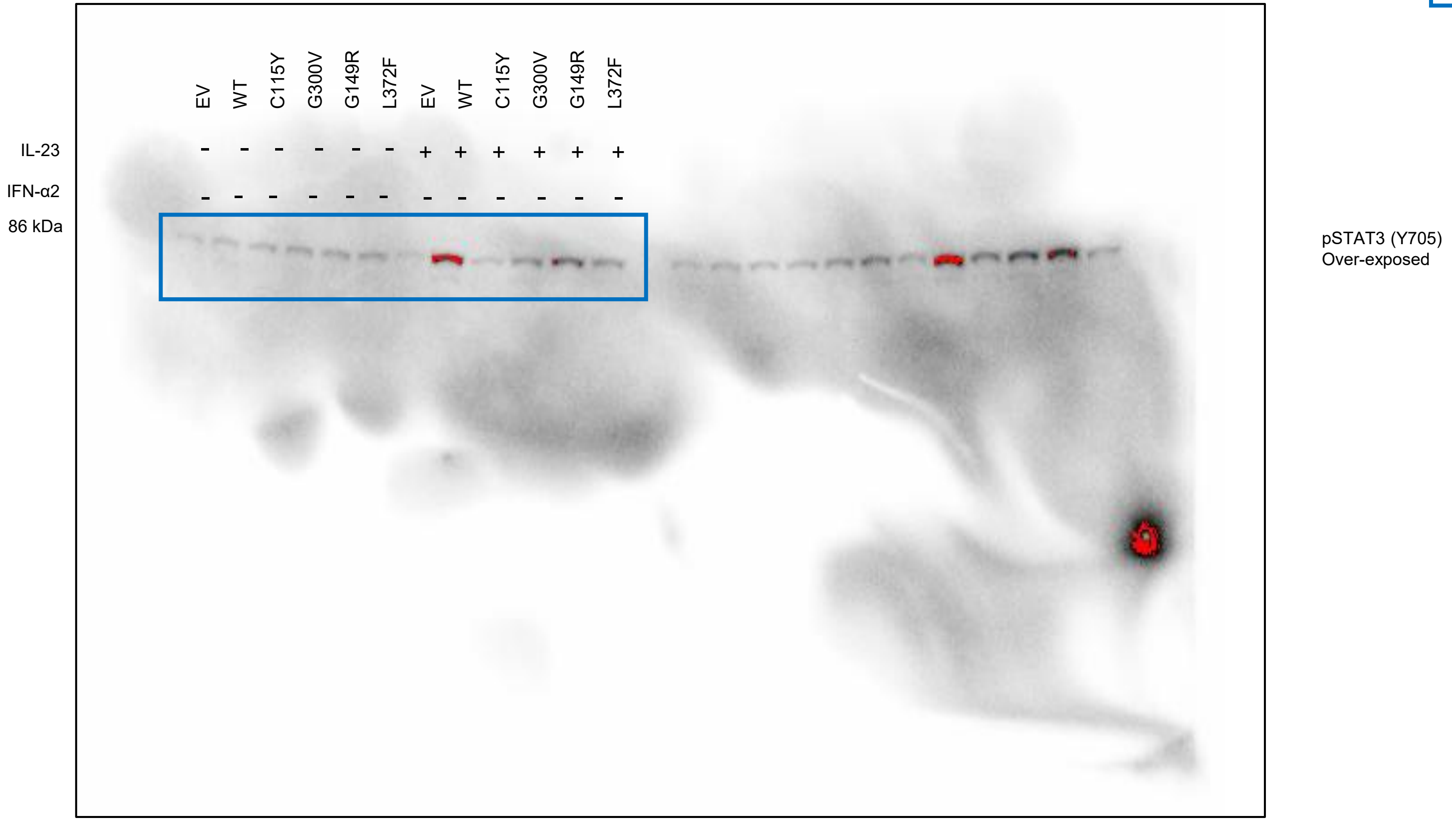

SourceDataF1

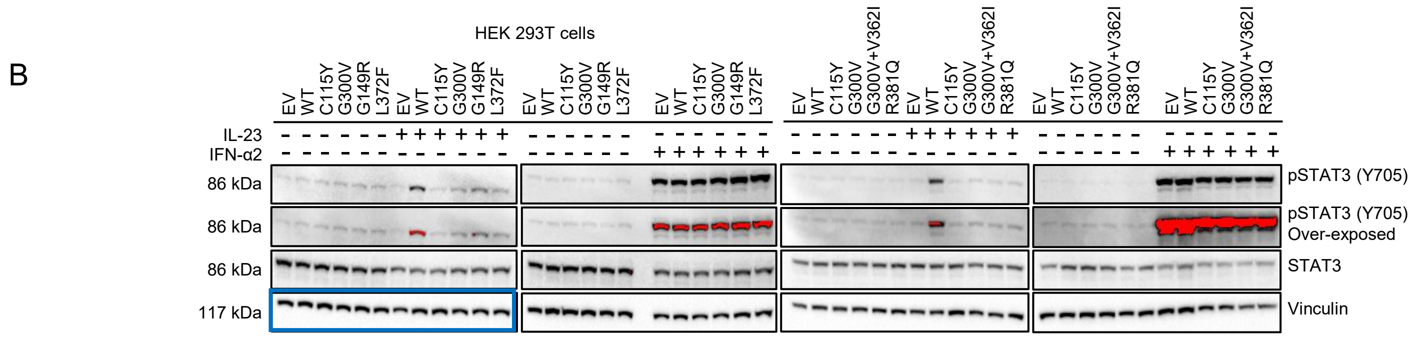

Showed

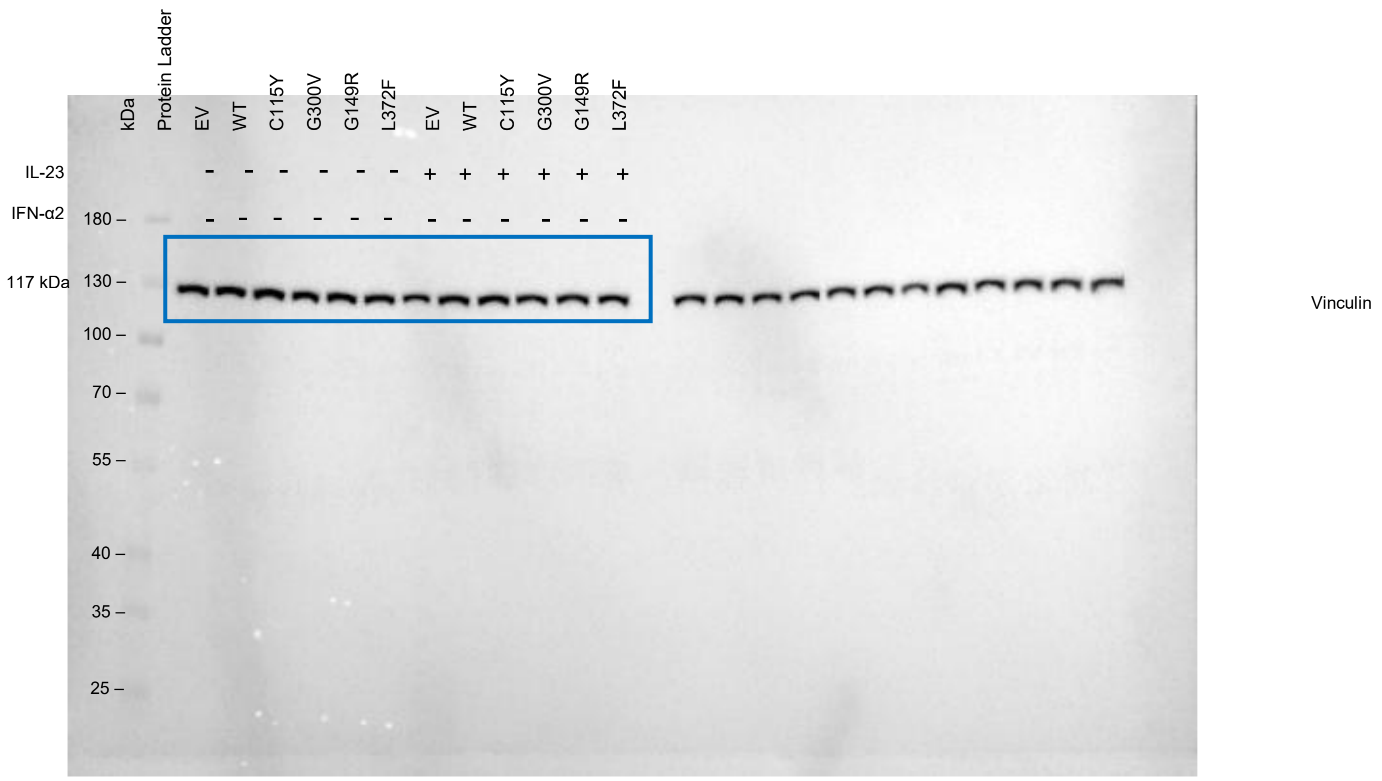

The ladder image was merged with the original image from the figure to visualize protein size

SourceDataF1

B

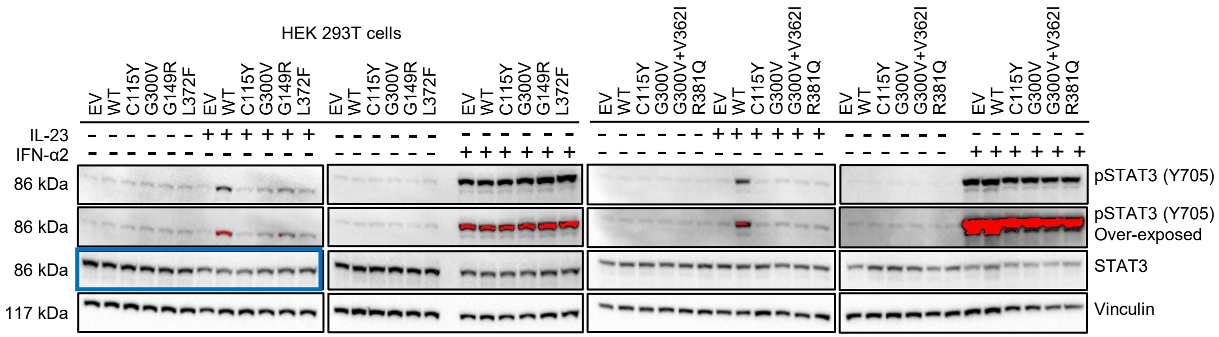

Showed

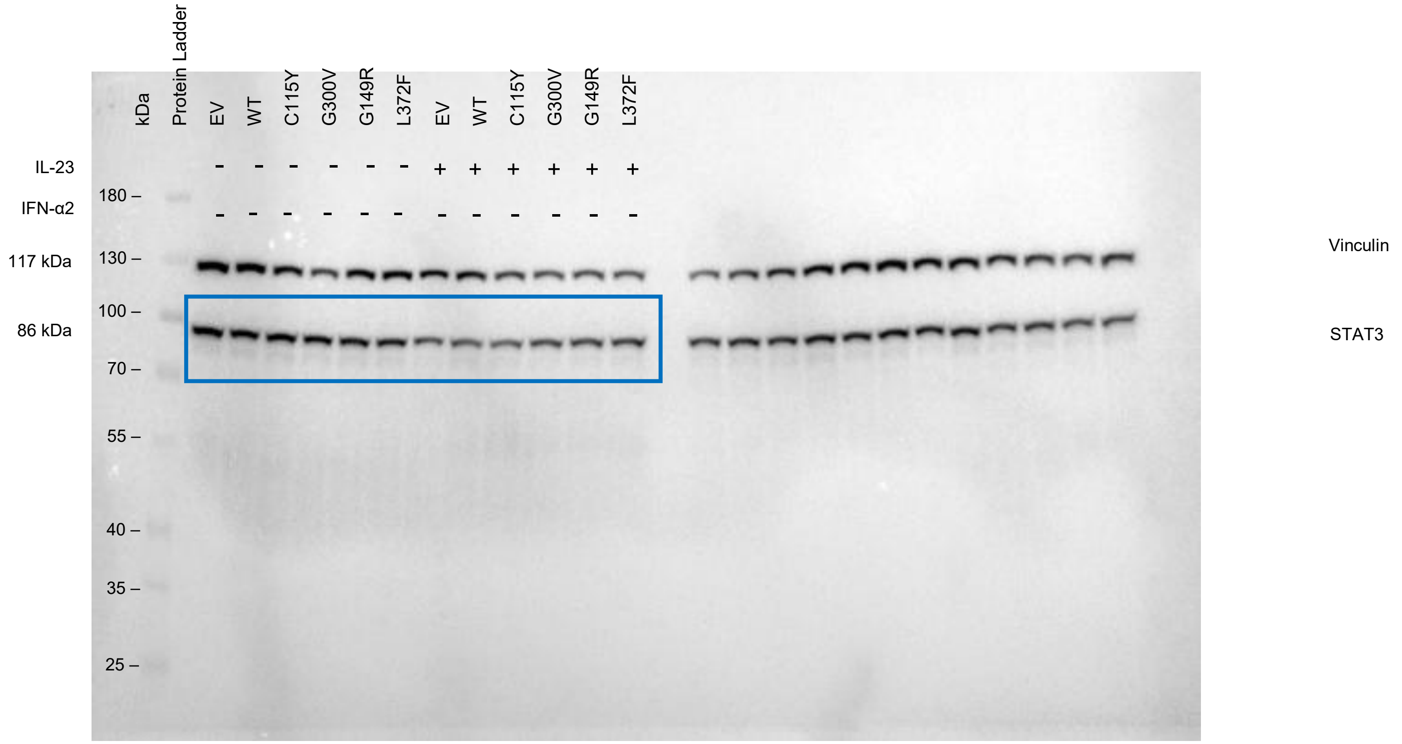

The ladder image was merged with the original image from the figure to visualize protein size

SourceDataF1

B

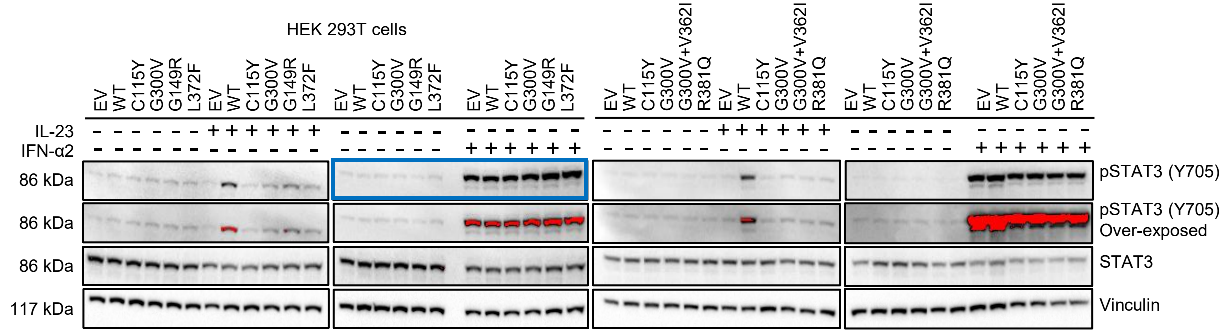

Showed

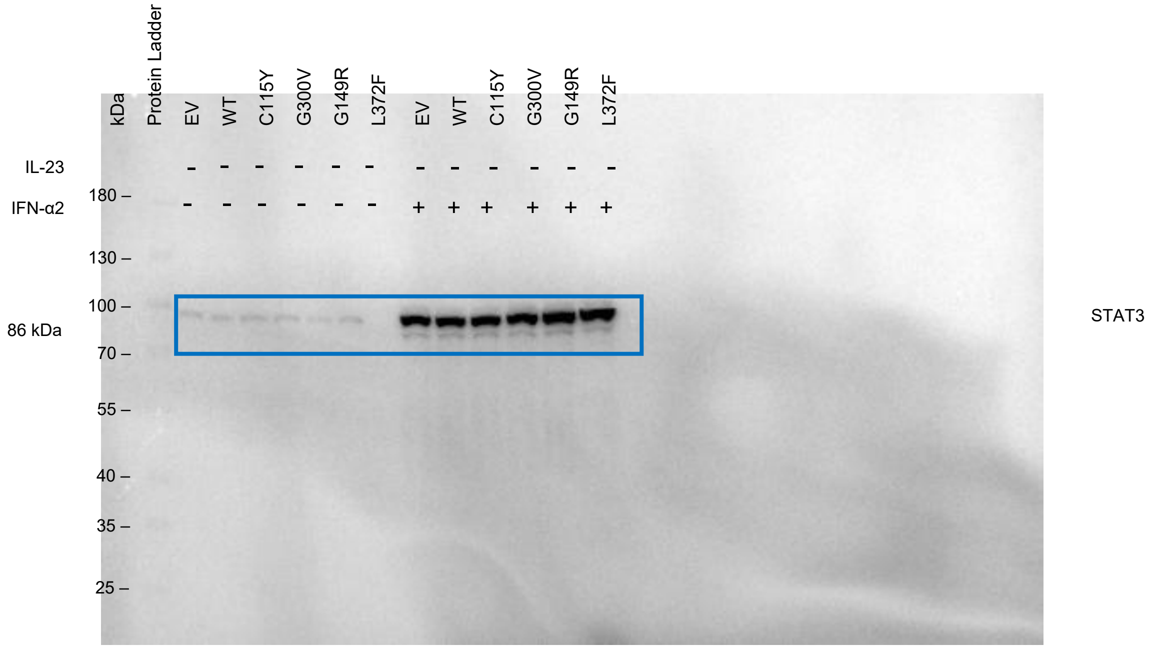

The ladder image was merged with the original image from the figure to visualize protein size

SourceDataF1

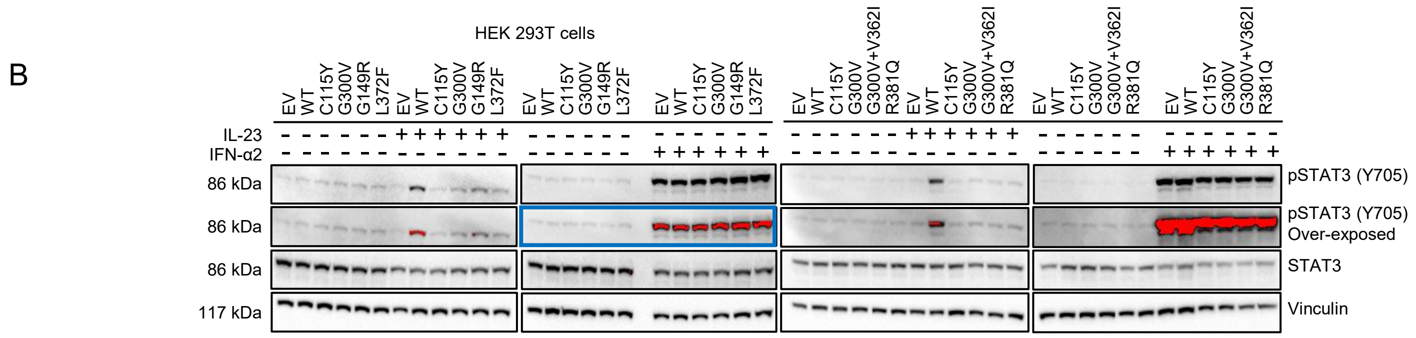

Showed

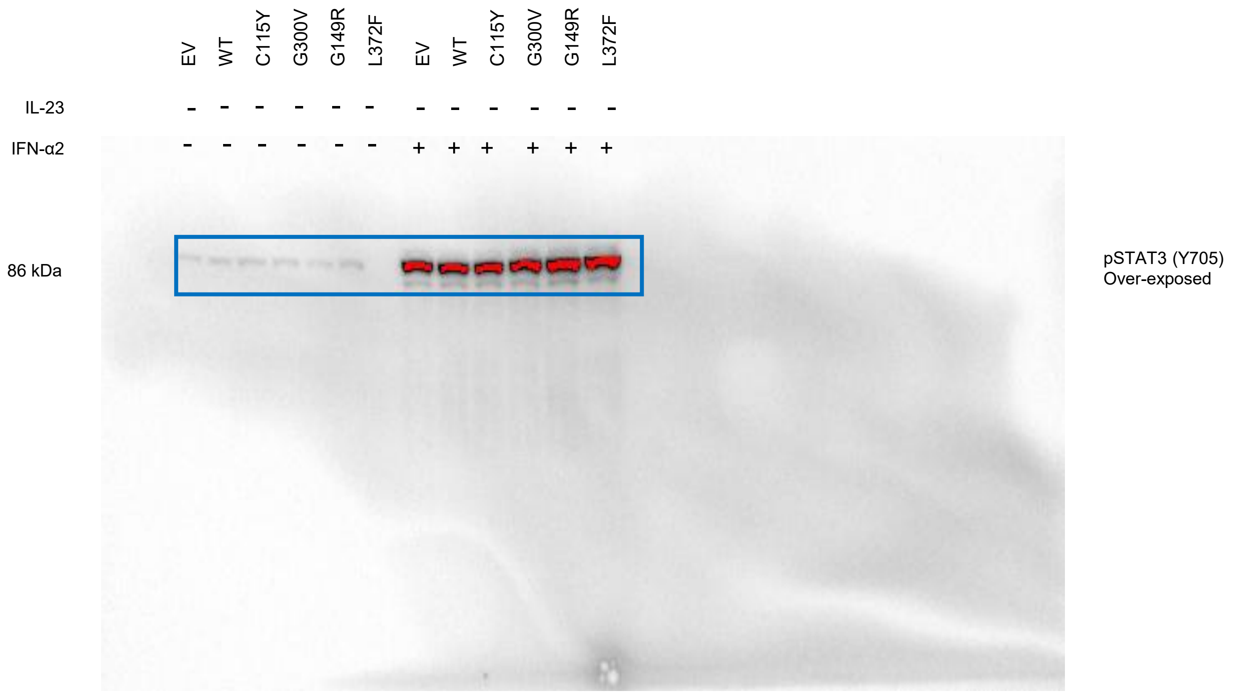

SourceDataF1

B

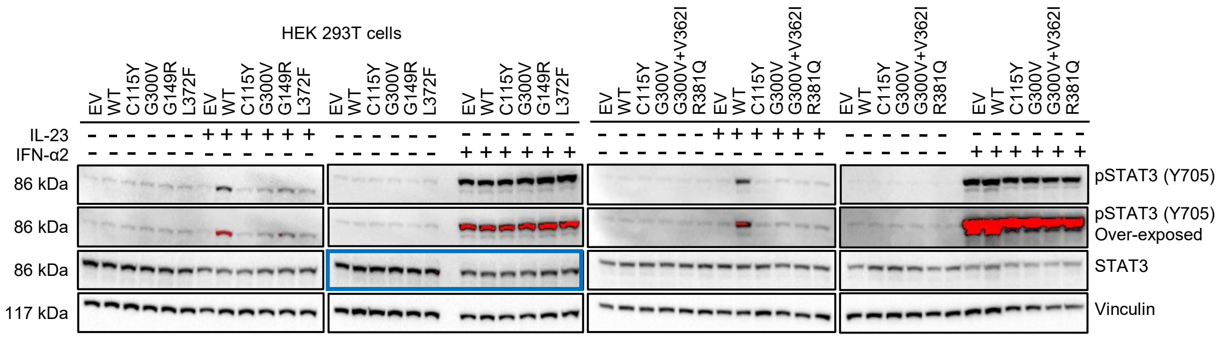

Showed

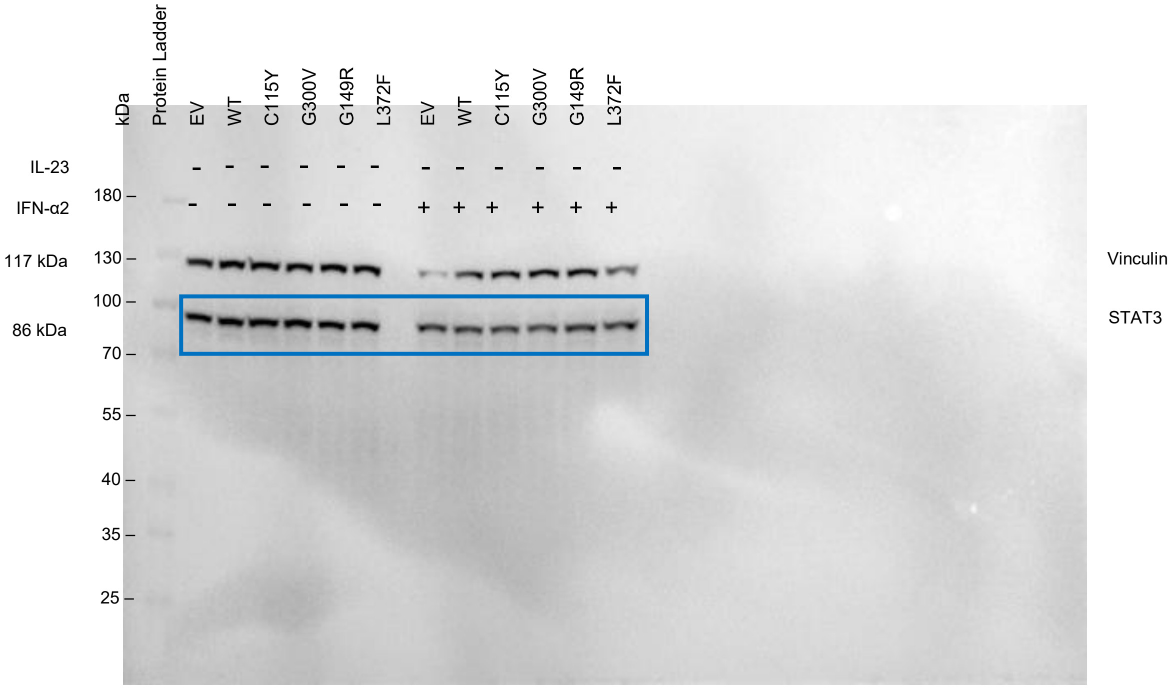

The ladder image was merged with the original image from the figure to visualize protein size

SourceDataF1

B

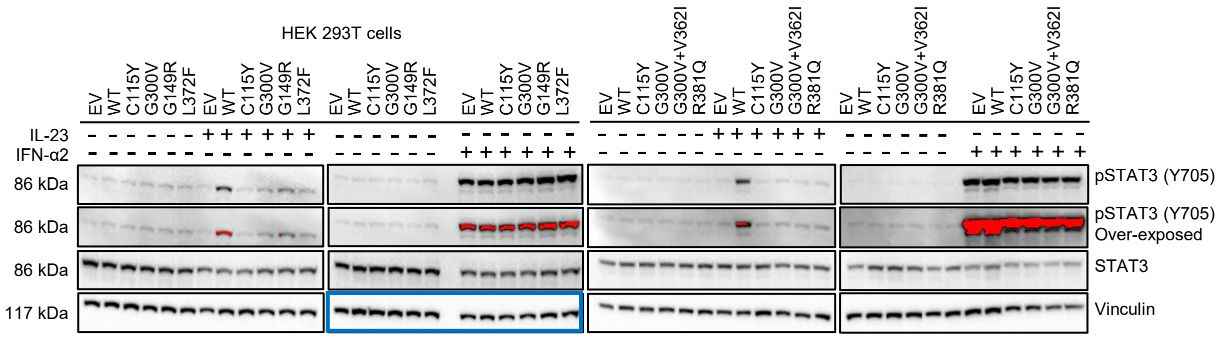

Showed

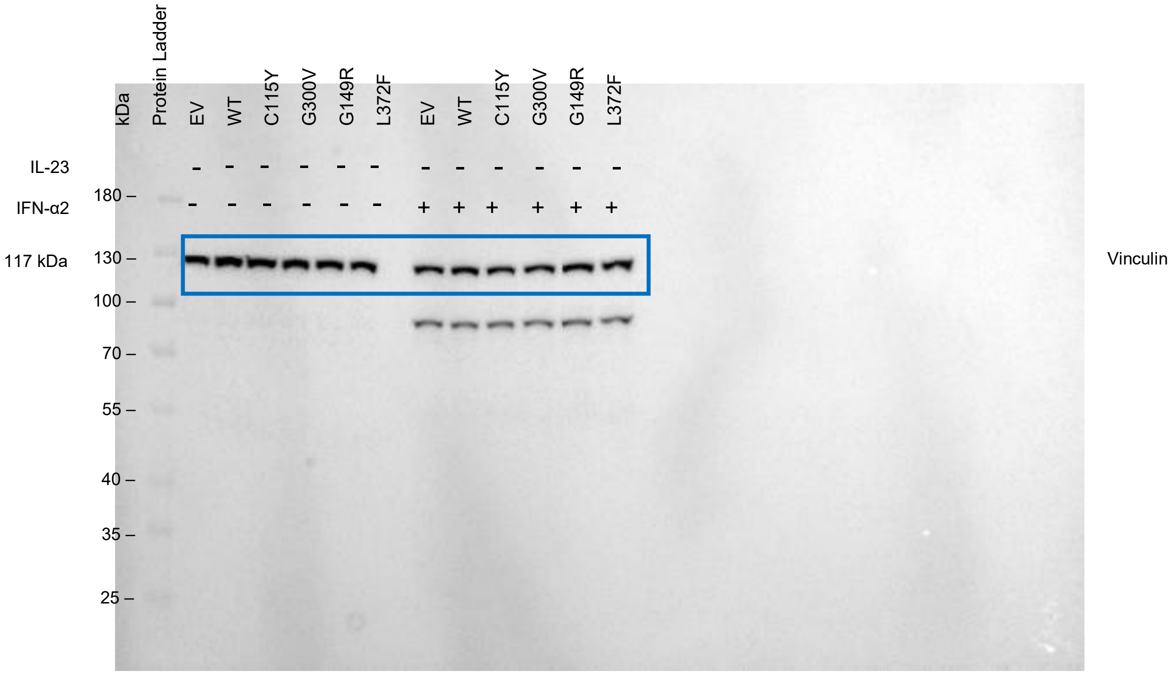

The ladder image was merged with the original image from the figure to visualize protein size

SourceDataF1

B

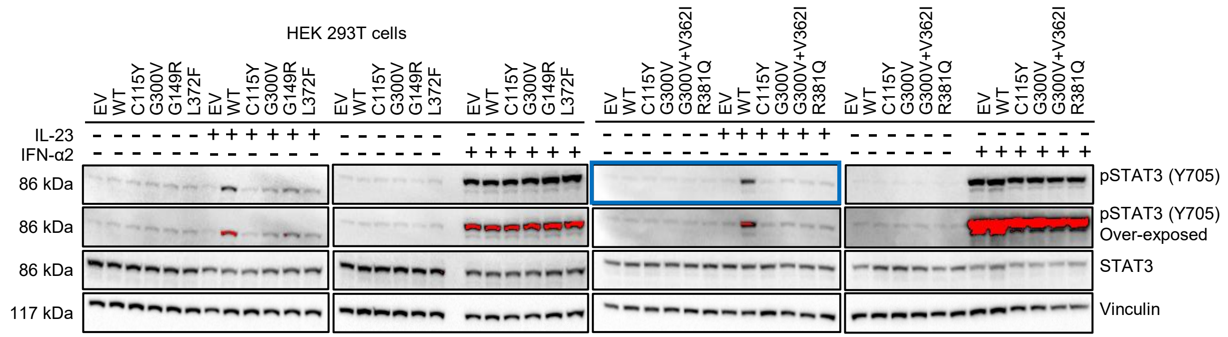

Showed

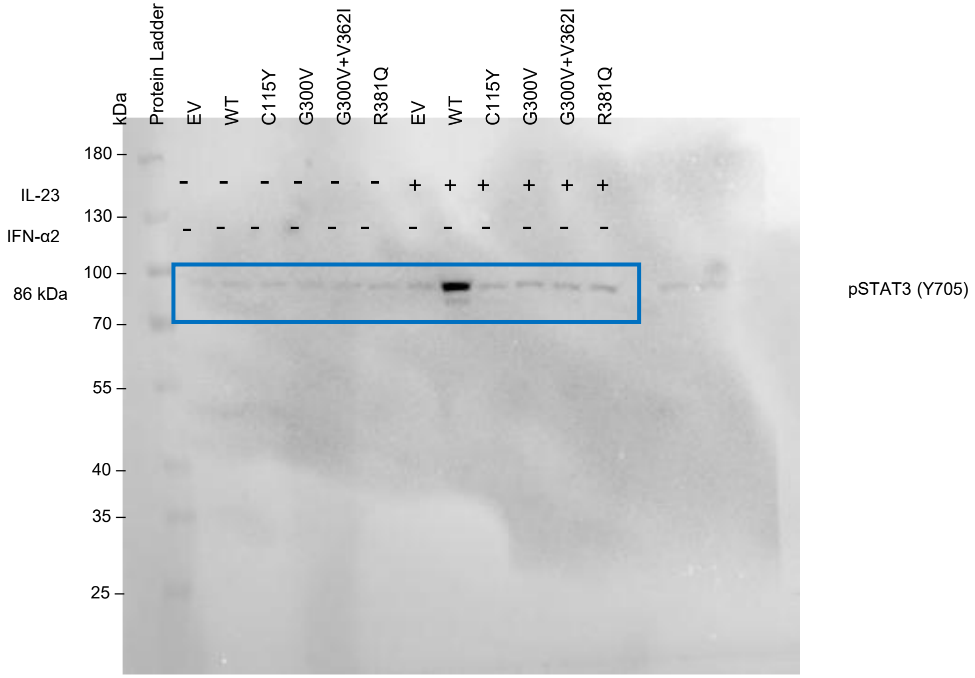

The ladder image was merged with the original image from the figure to visualize protein size

SourceDataF1

B

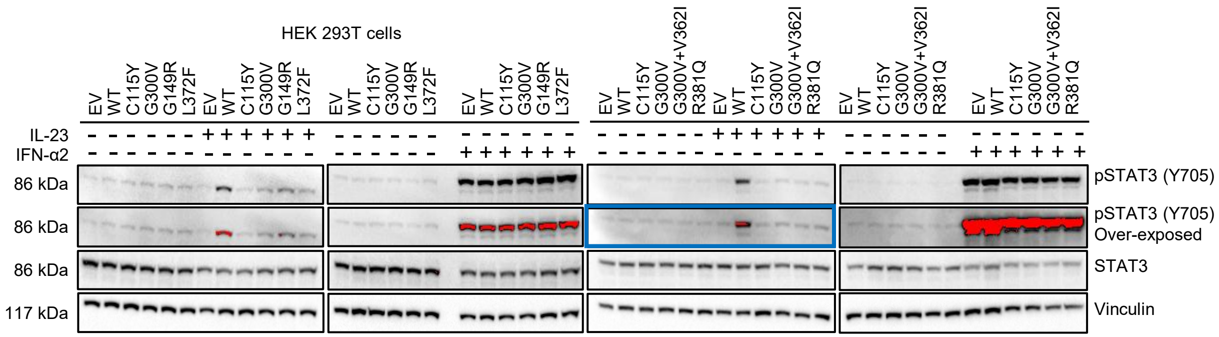

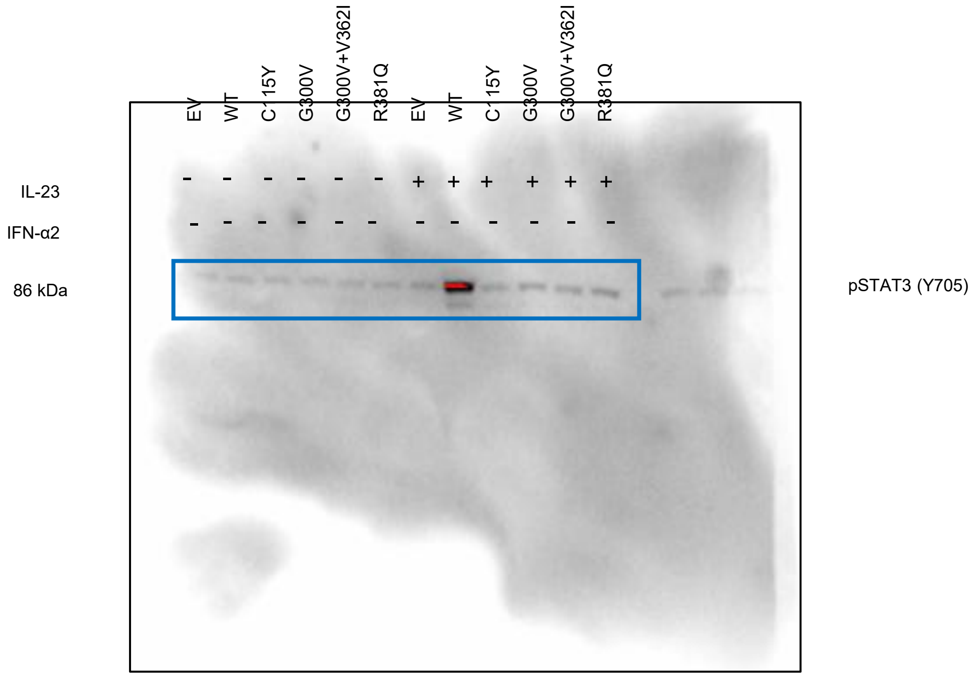

Showed

SourceDataF1

B

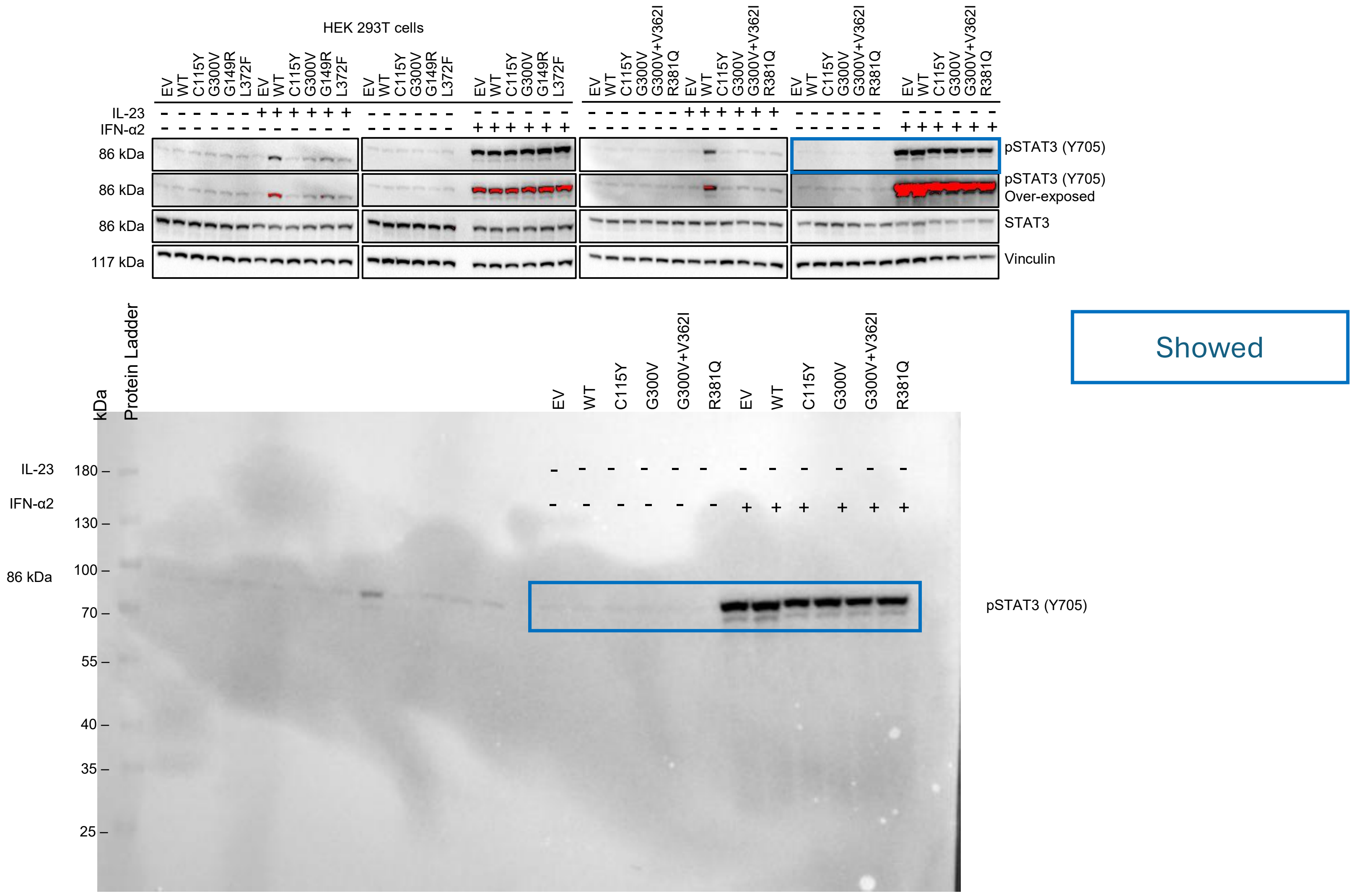

The ladder image was merged with the original image from the figure to visualize protein size

SourceDataF1

B

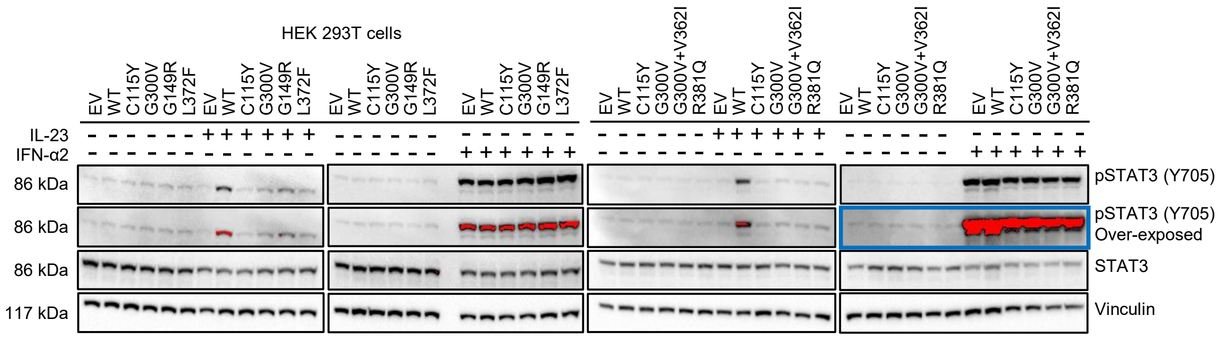

Showed

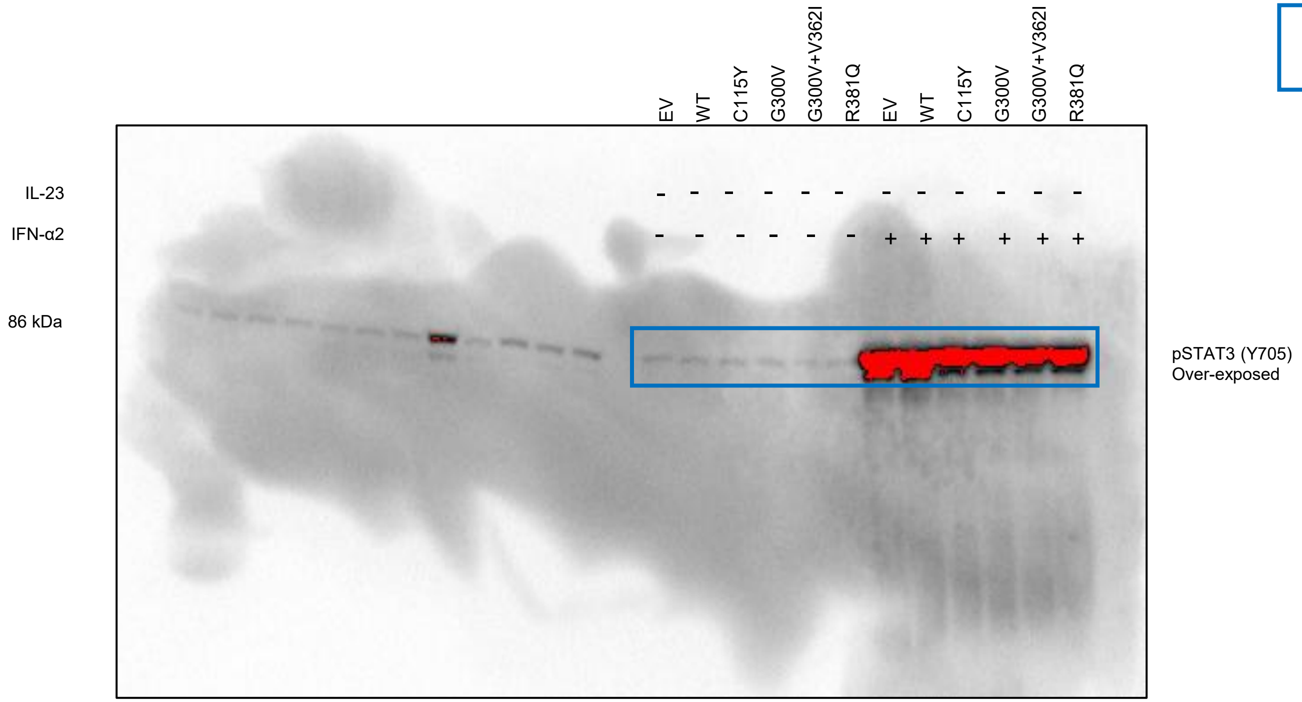

The ladder image was merged with the original image from the figure to visualize protein size

SourceDataF1

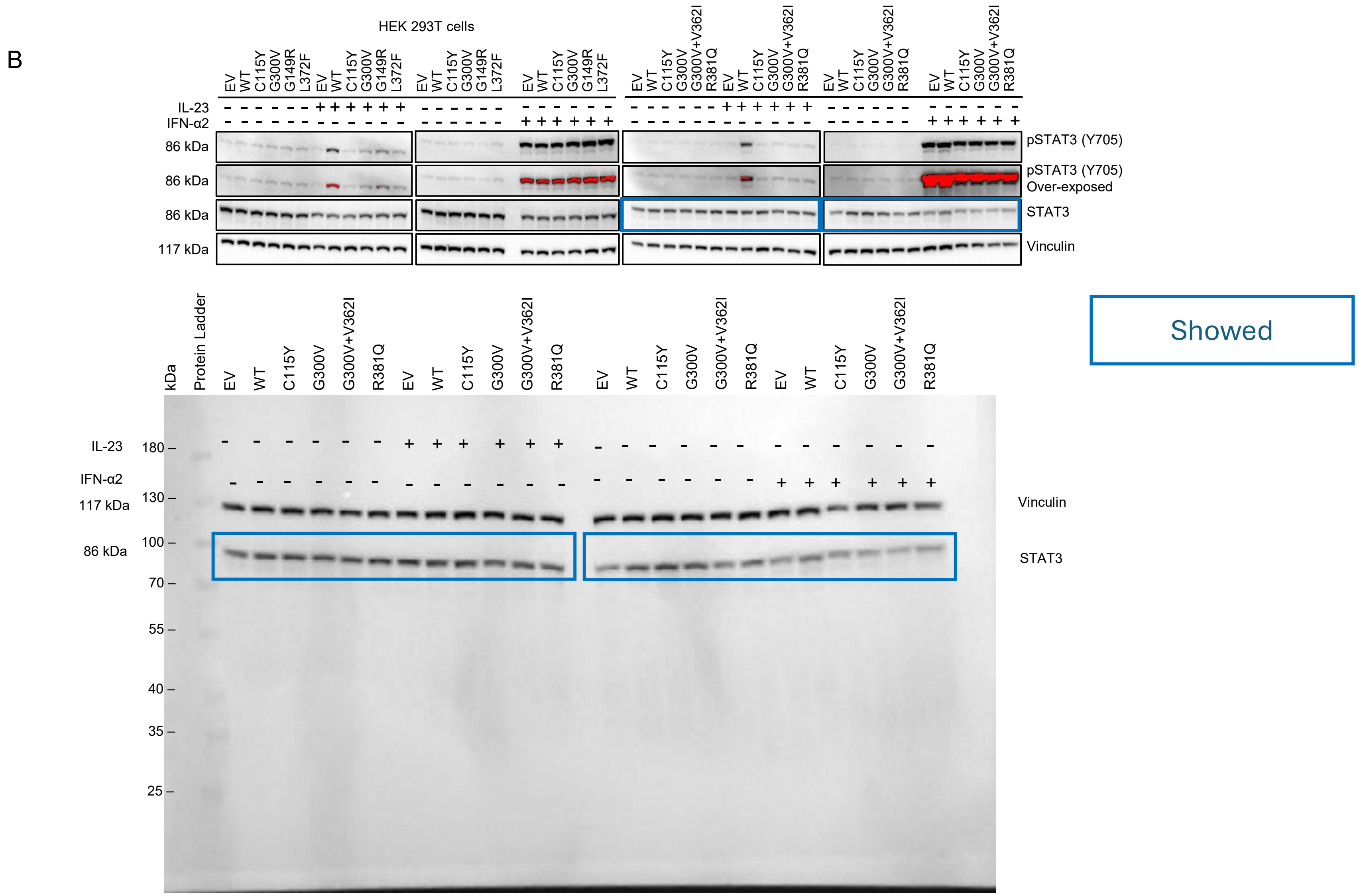

The ladder image was merged with the original image from the figure to visualize protein size

SourceDataF1

B

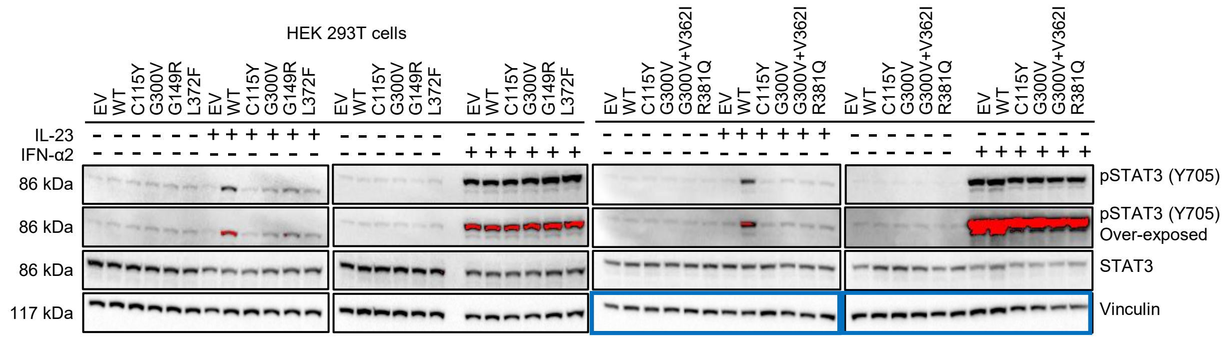

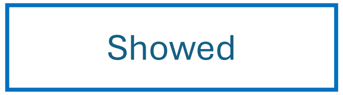

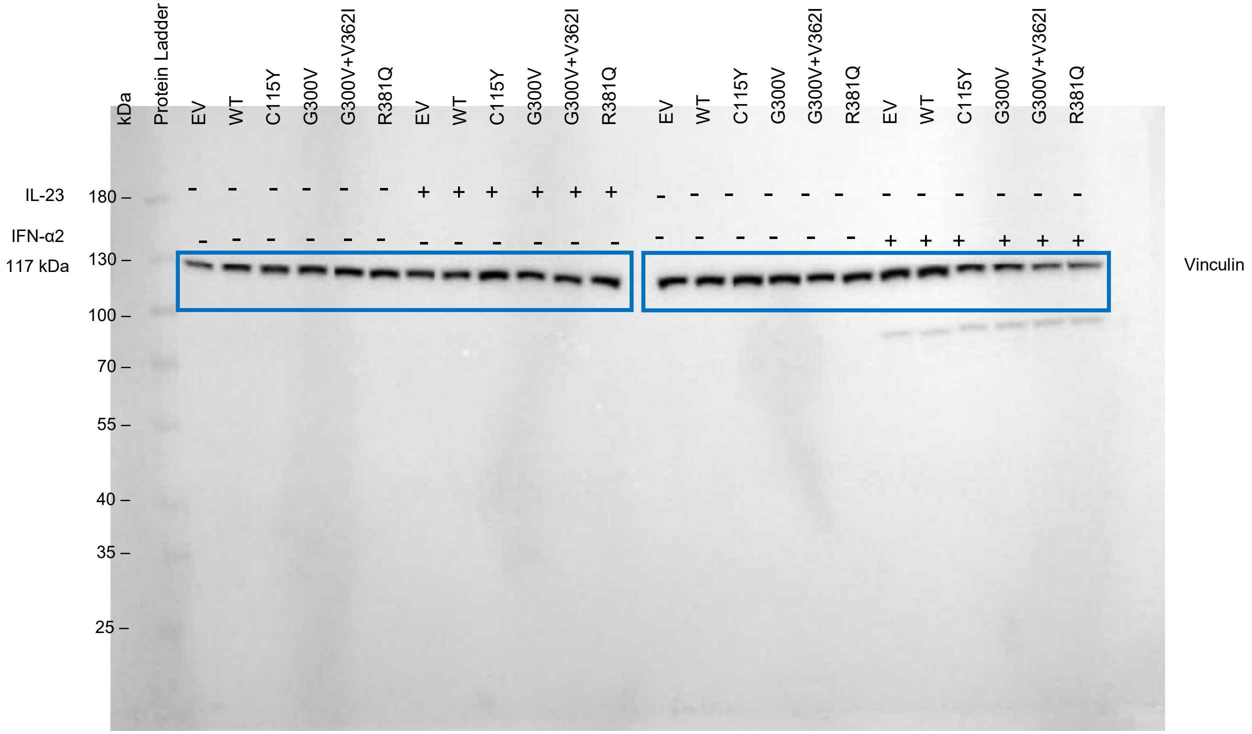

The ladder image was merged with the original image from the figure to visualize protein size
