# Supplementary material for: Humans homozygous for rare or common hypomorphic IL23R variants are prone to tuberculosis
Source: J Exp Med. 2026 Jul 9;223(8):e20252236. doi: 10.1084/jem.20252236 (PMC13348821; doi:10.1084/jem.20252236)
Supplement: SourceData F2 — is the source file for Fig. 2. [file jem_20252236_sourcedataf2.pdf]

SourceDataF2

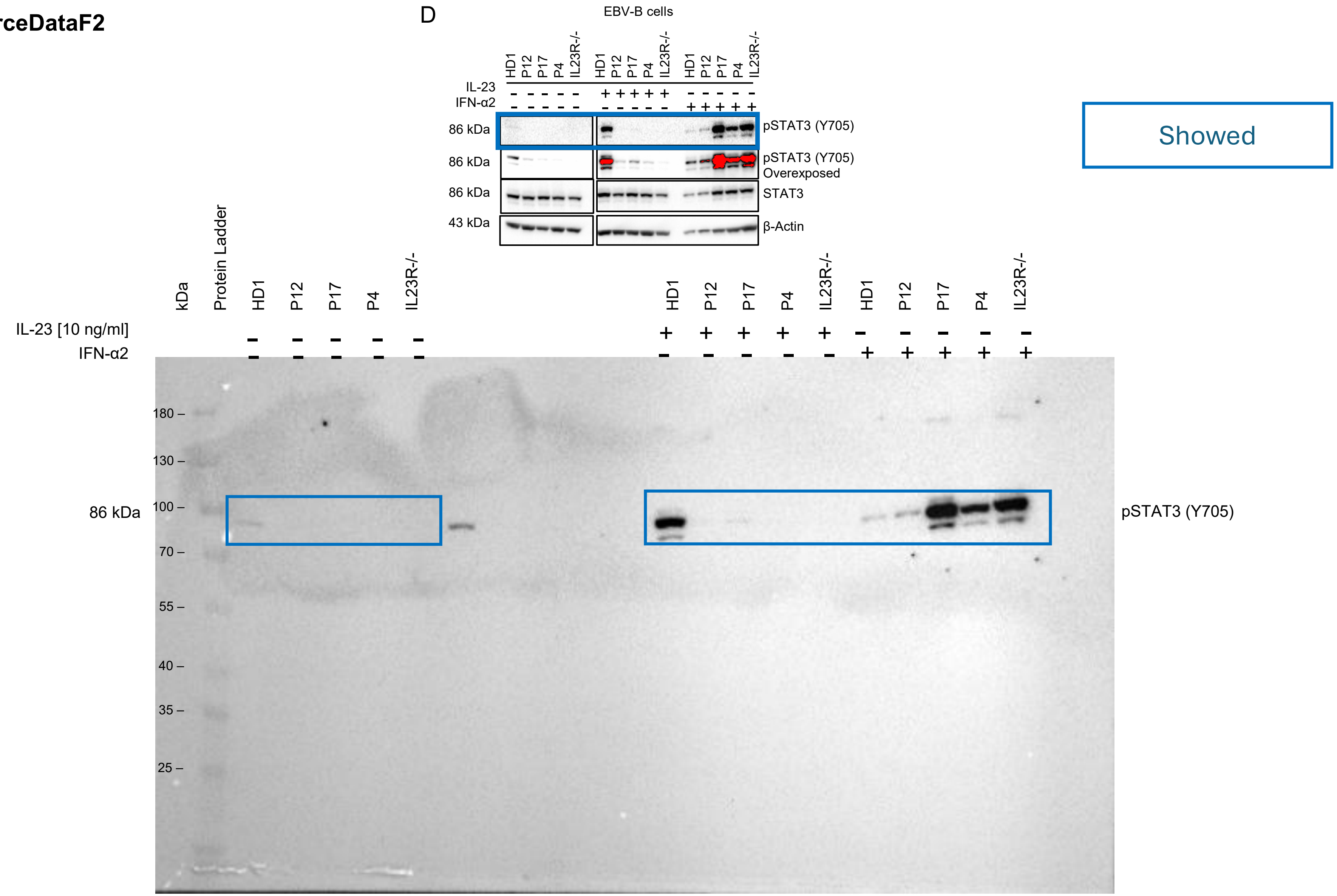

SourceDataF2

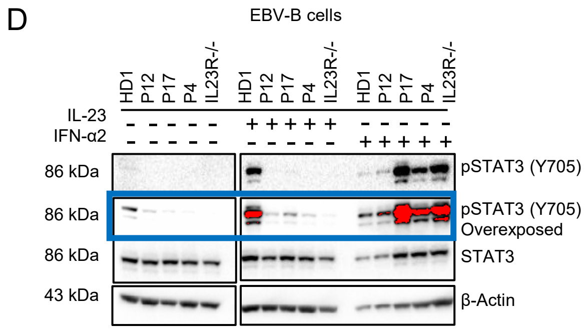

Showed

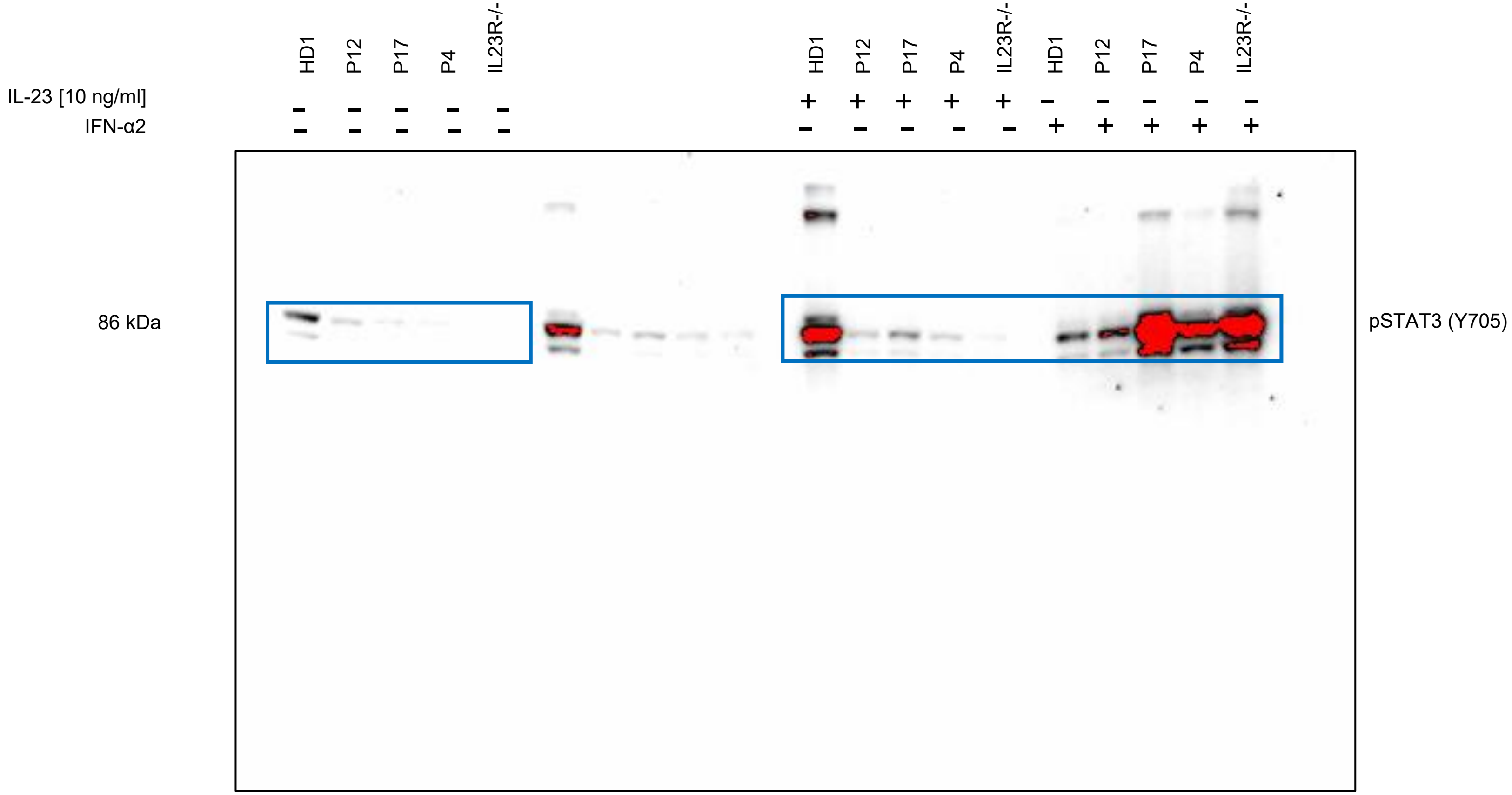

SourceDataF2

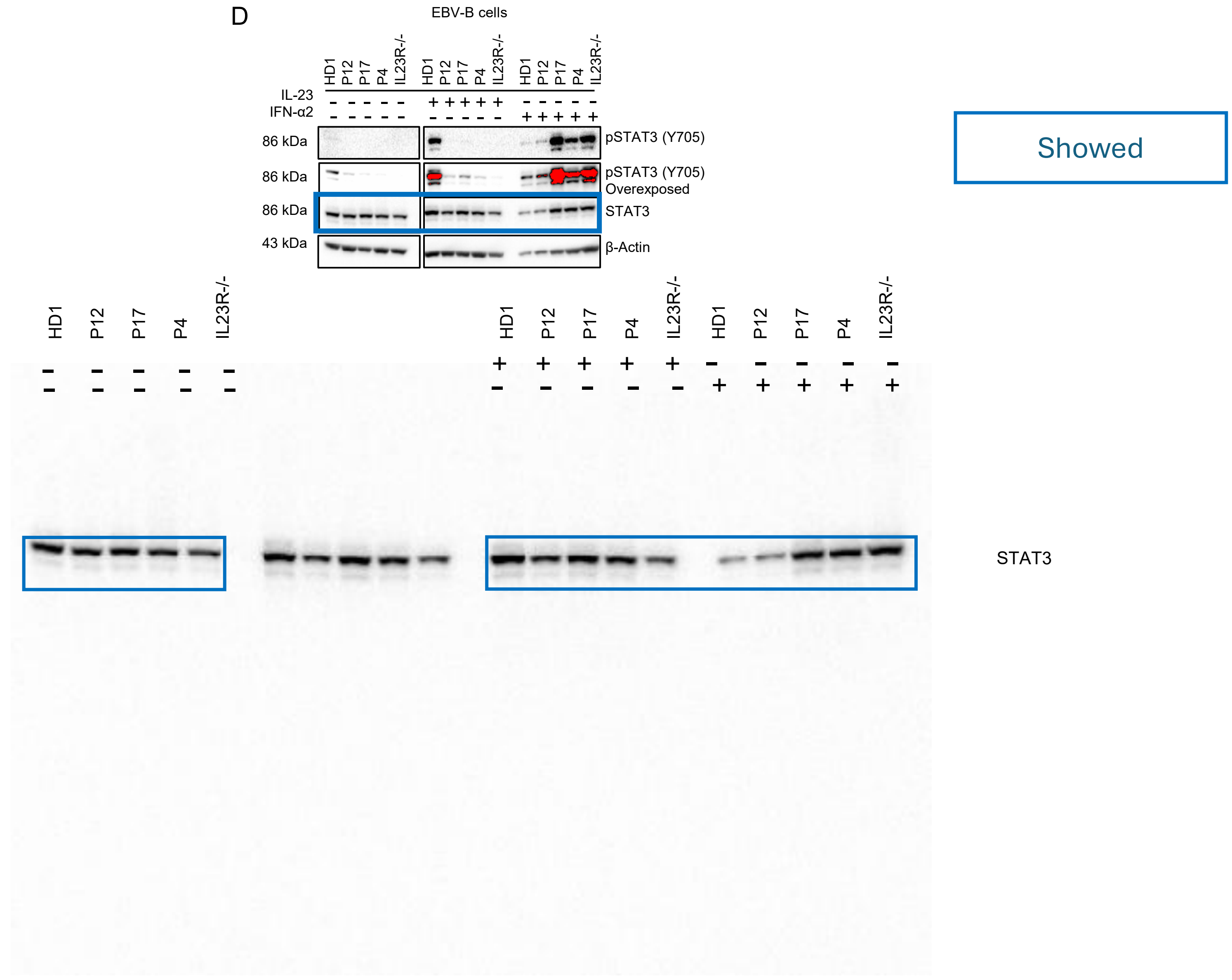

SourceDataF2

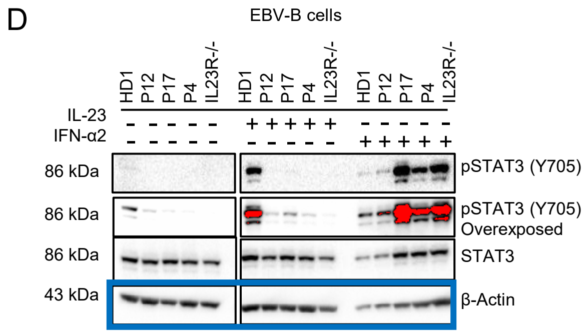

Showed

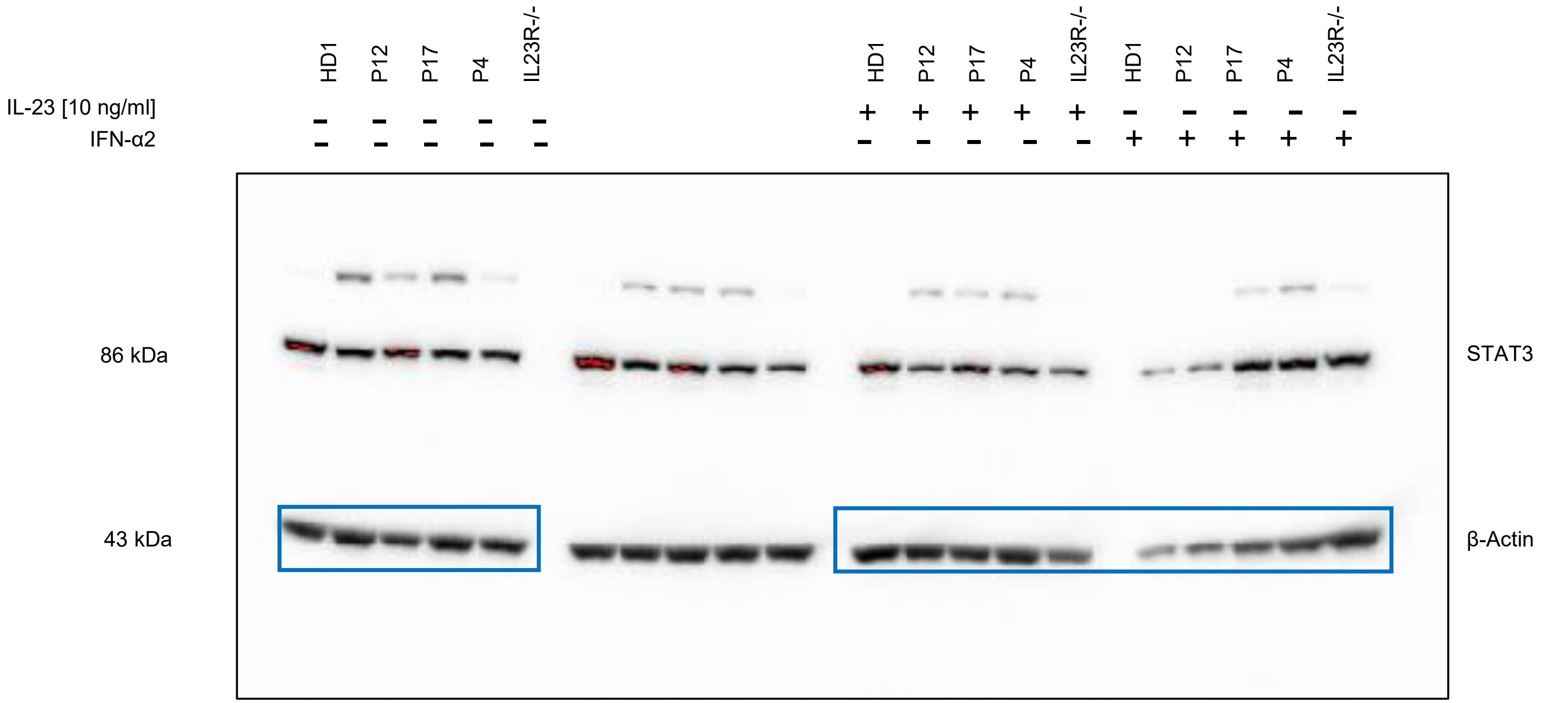

SourceDataF2

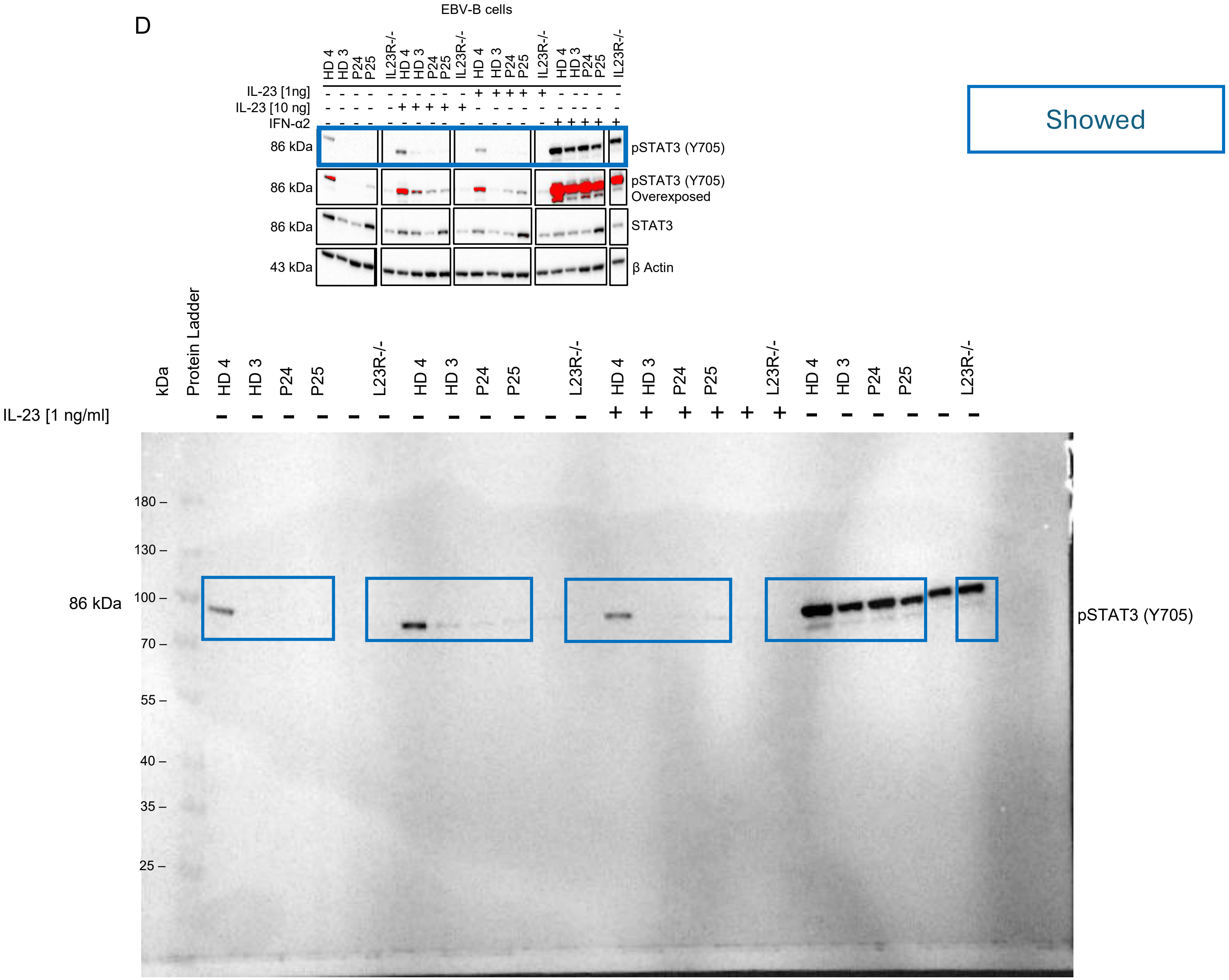

The ladder image was merged with the original image from the figure to visualize protein size

SourceDataF2

D

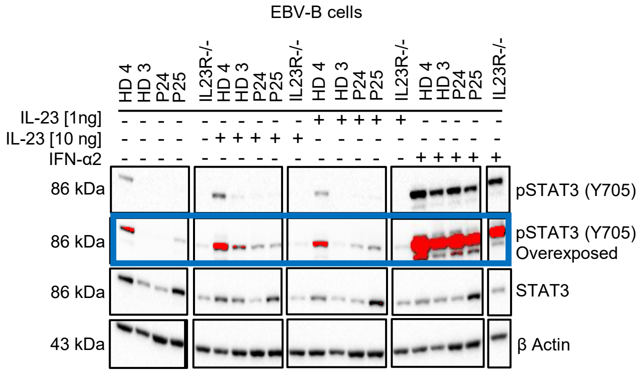

Showed

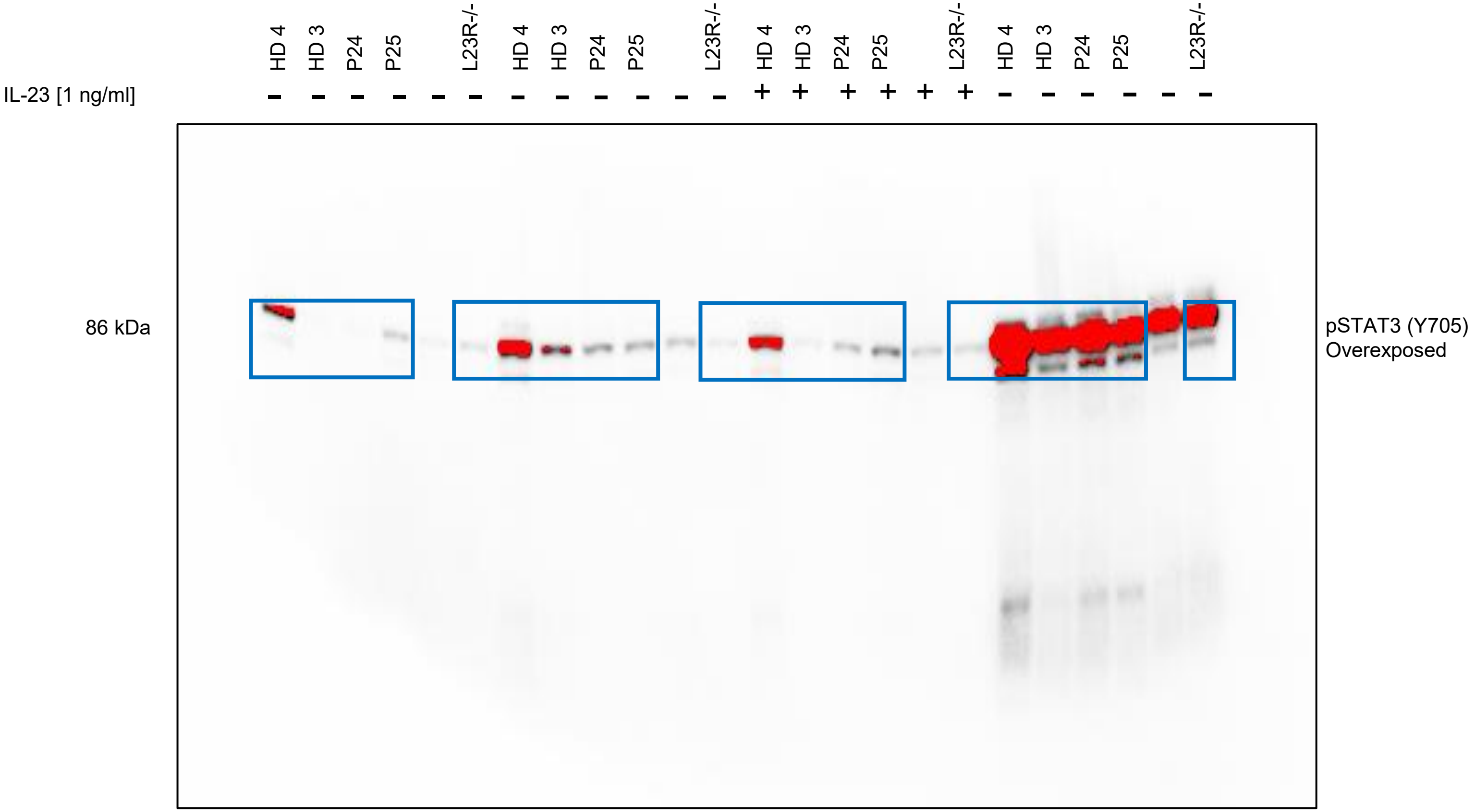

SourceDataF2

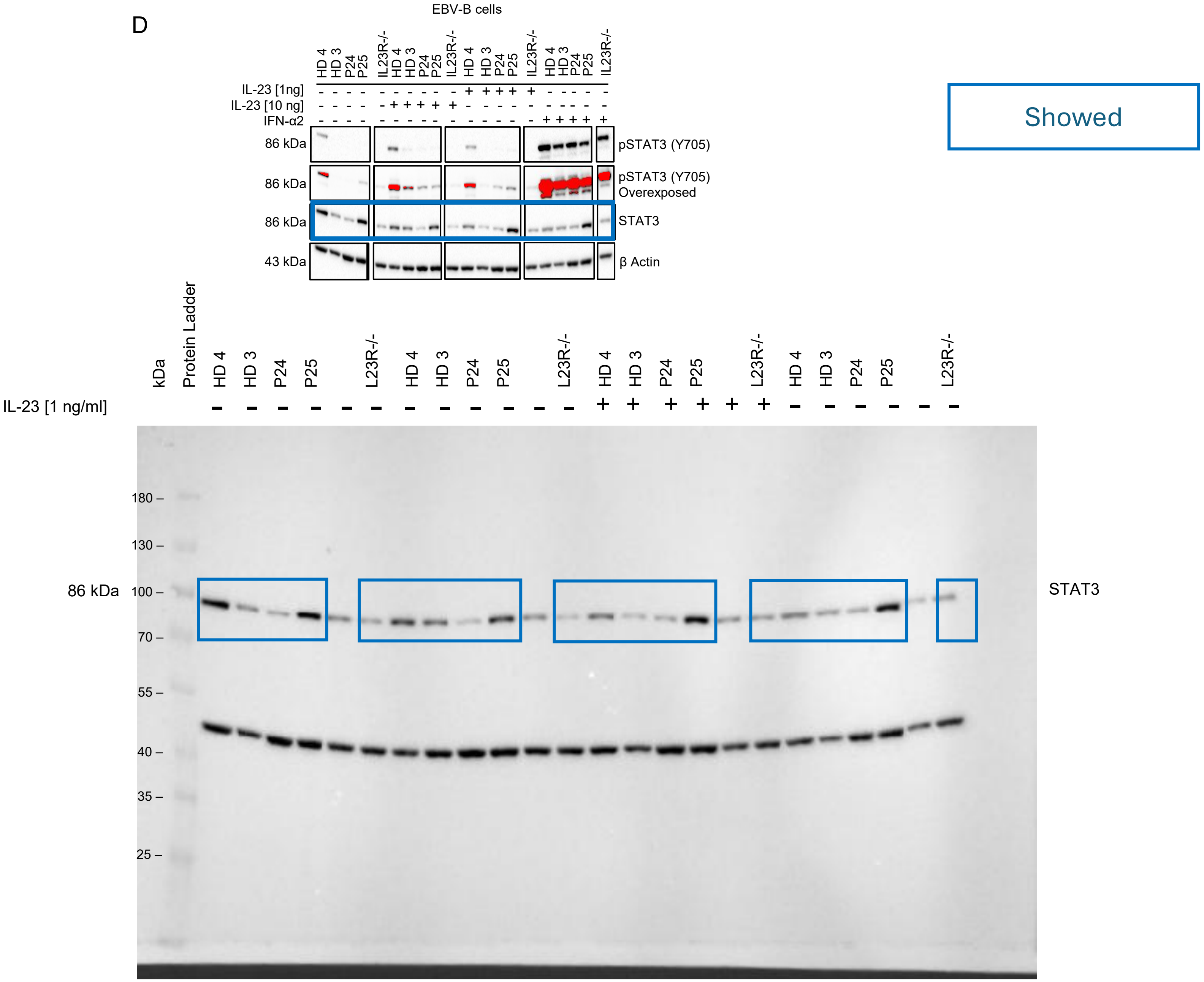

The ladder image was merged with the original image from the figure to visualize protein size

SourceDataF2

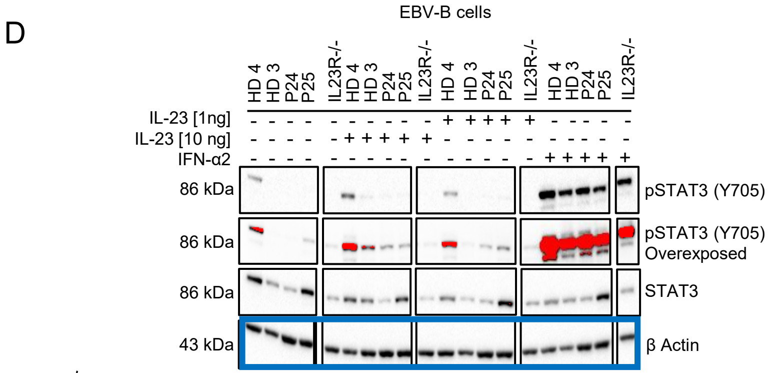

Showed

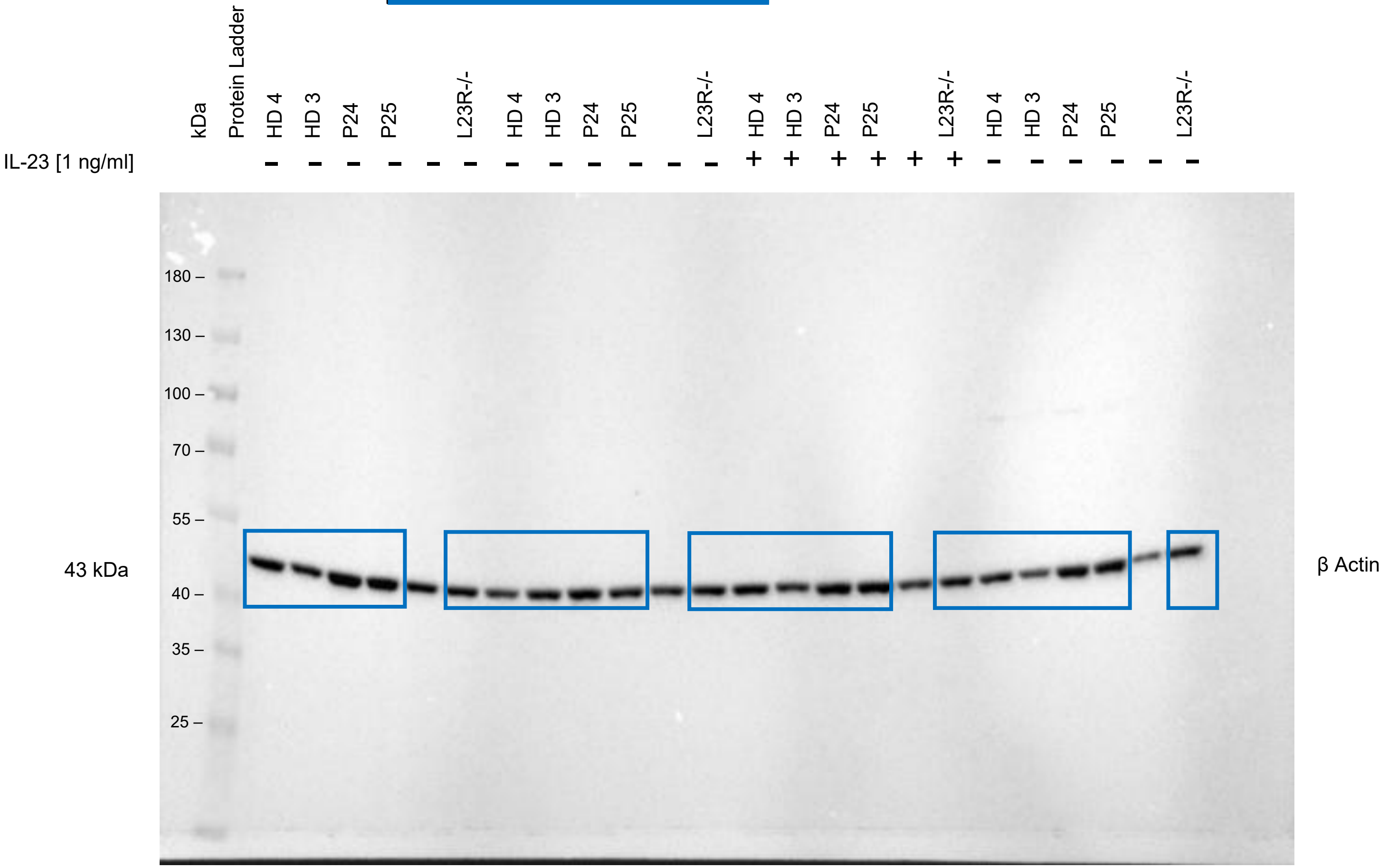

The ladder image was merged with the original image from the figure to visualize protein size
