# Supplementary material for: Humans homozygous for rare or common hypomorphic IL23R variants are prone to tuberculosis
Source: J Exp Med. 2026 Jul 9;223(8):e20252236. doi: 10.1084/jem.20252236 (PMC13348821; doi:10.1084/jem.20252236)
Supplement: SourceData FS1 — is the source file for Fig. S1. [file jem_20252236_sourcedatafs1.pdf]

SourceDataFS1

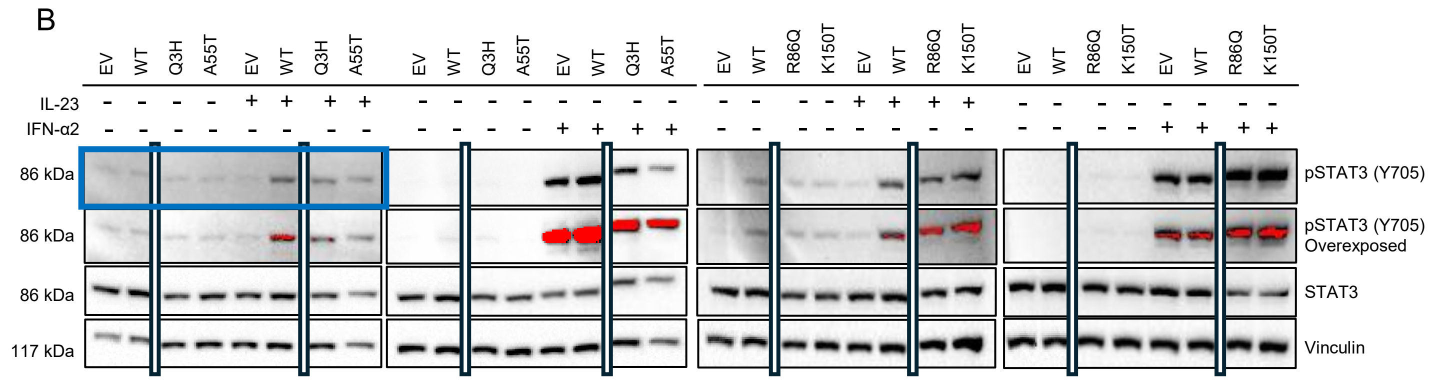

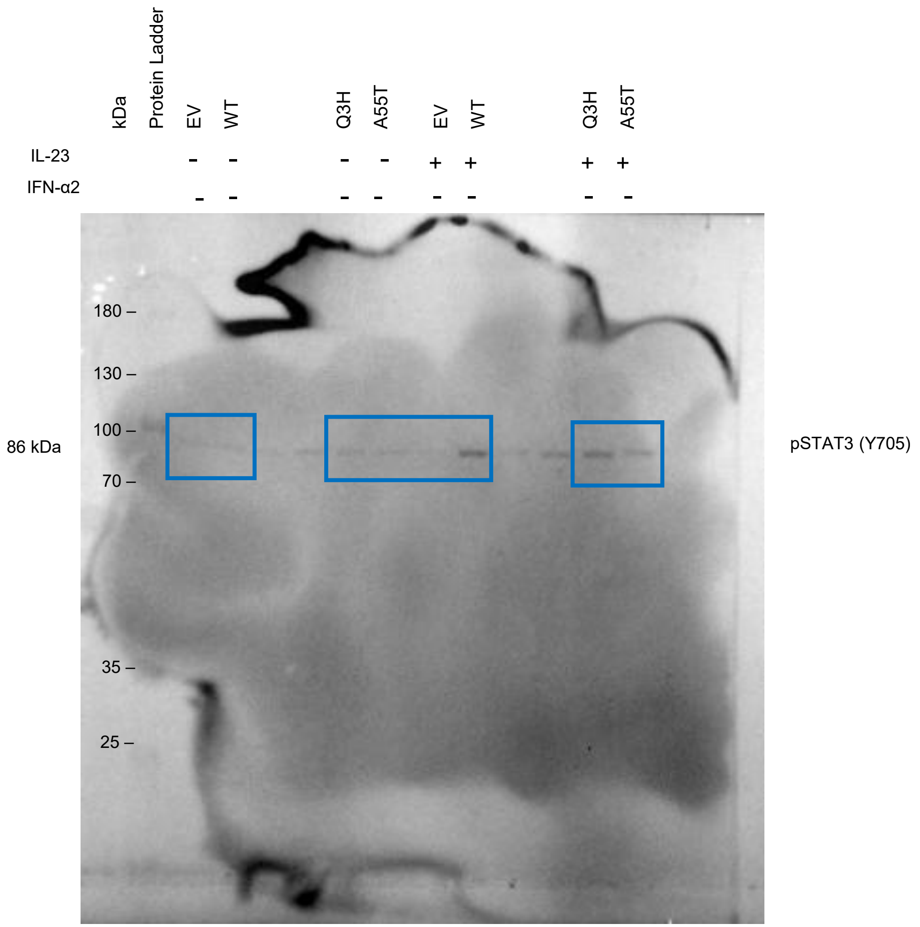

Showed

The ladder image was merged with the original image from the figure to visualize protein size

SourceDataFS1

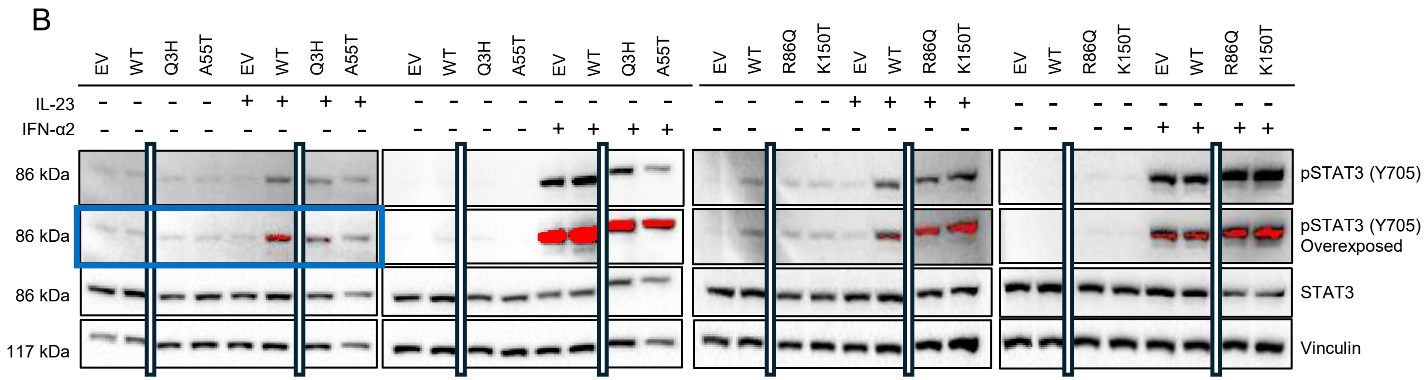

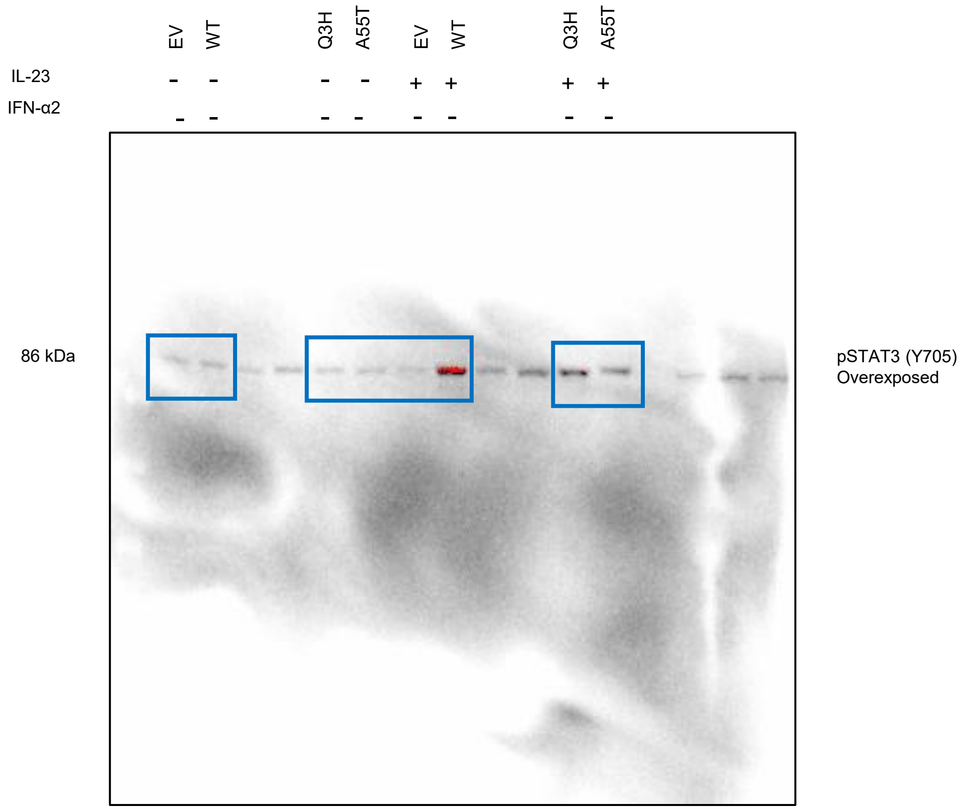

Showed

SourceDataFS1

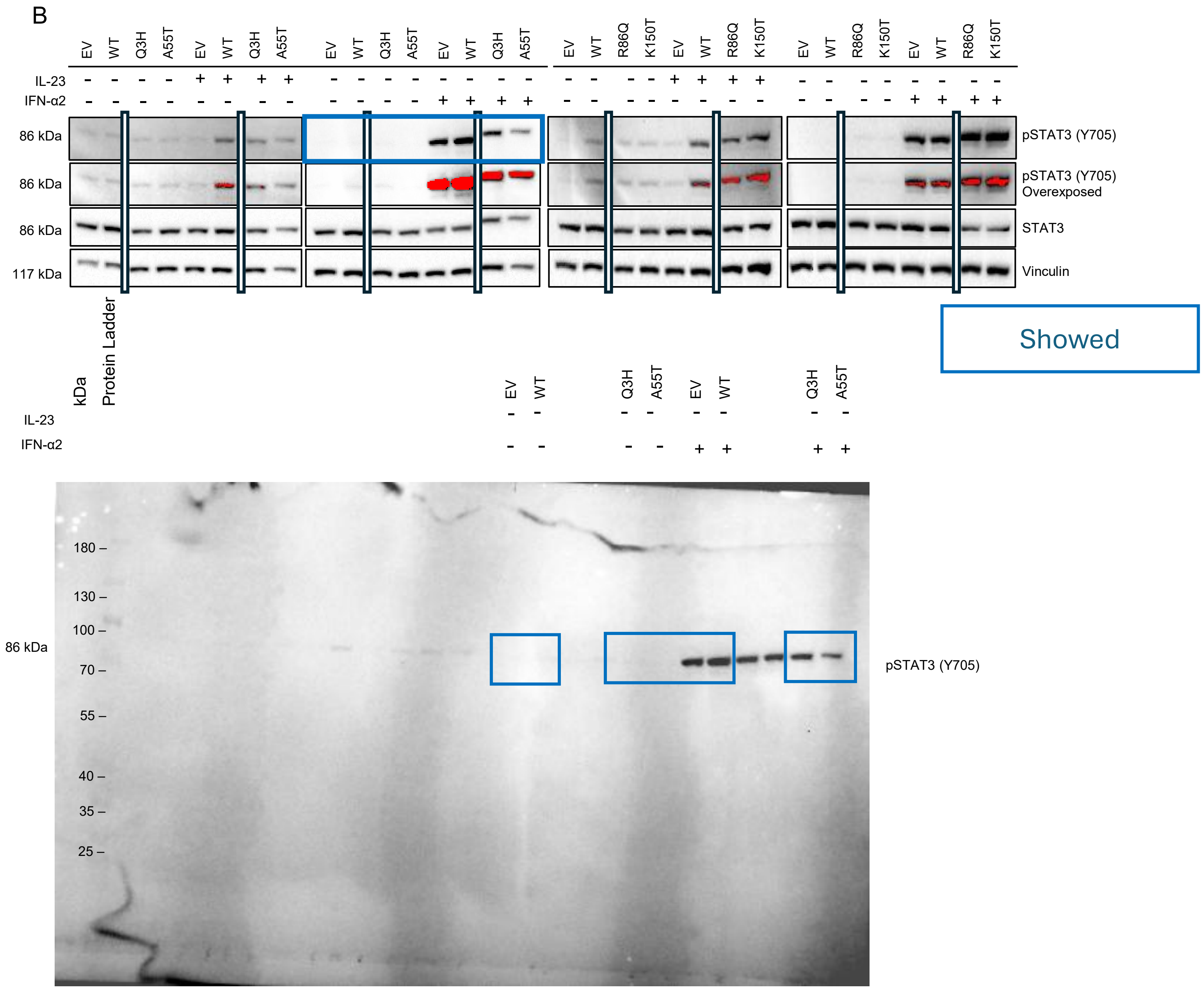

The ladder image was merged with the original image from the figure to visualize protein size

SourceDataFS1

B

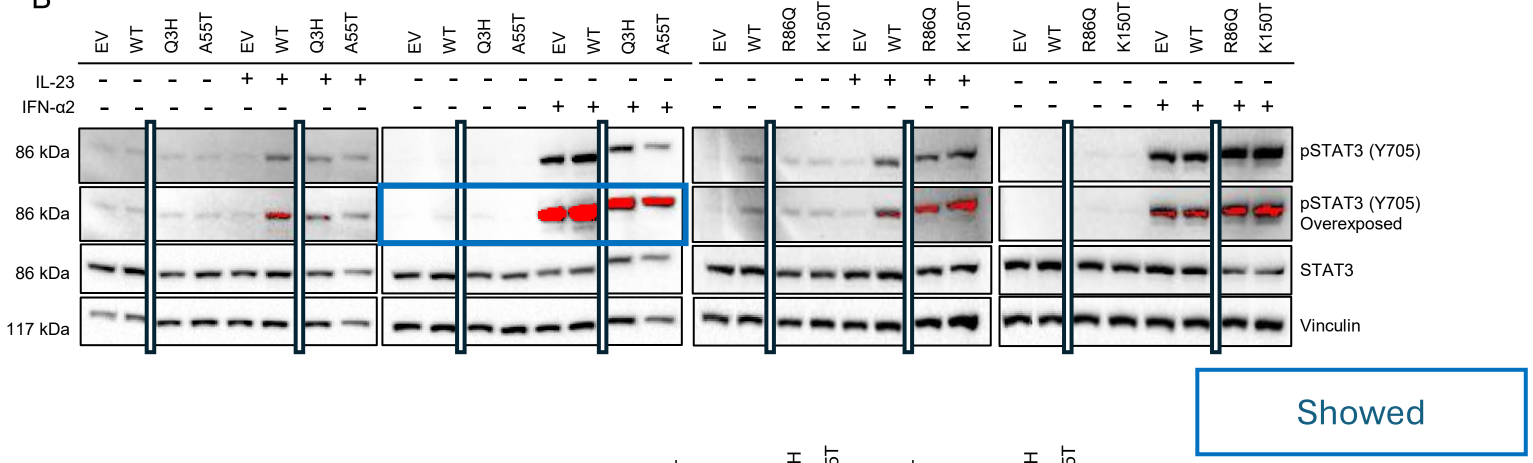

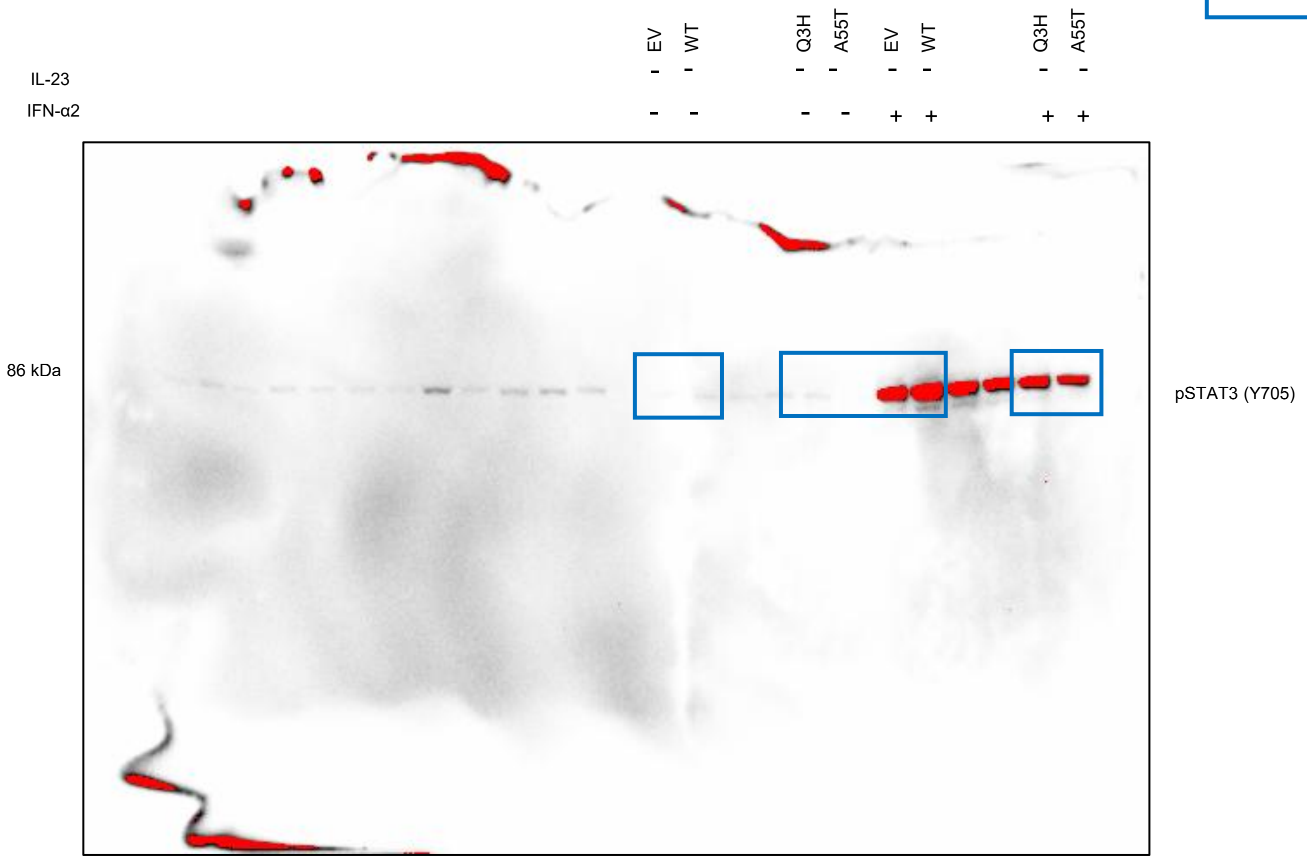

SourceDataFS1

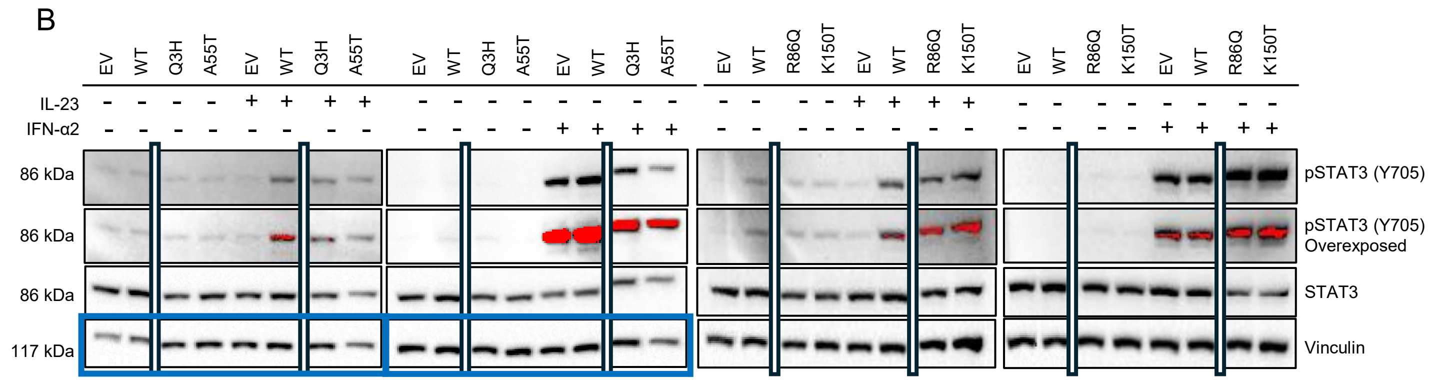

Showed

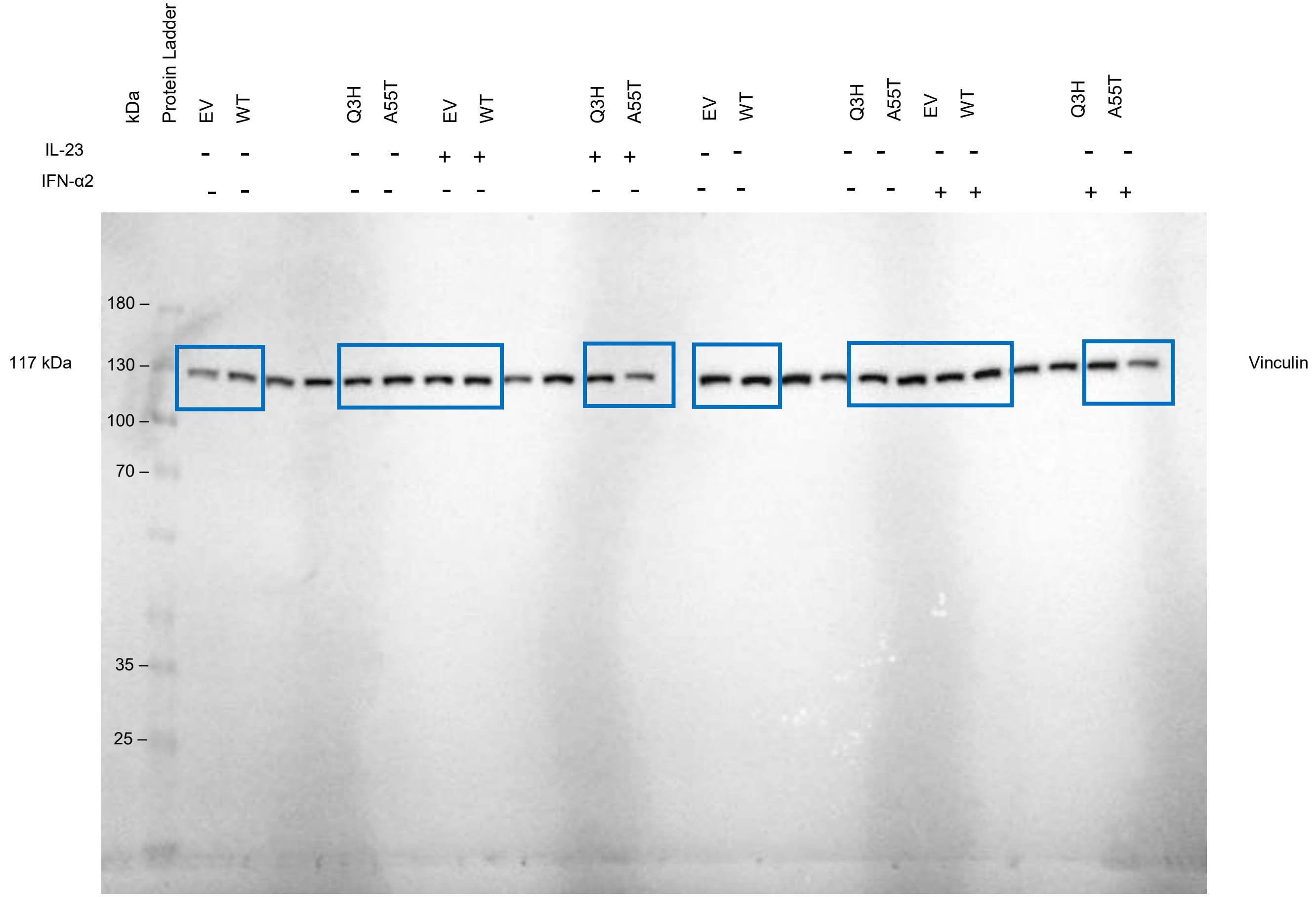

The ladder image was merged with the original image from the figure to visualize protein size

SourceDataFS1

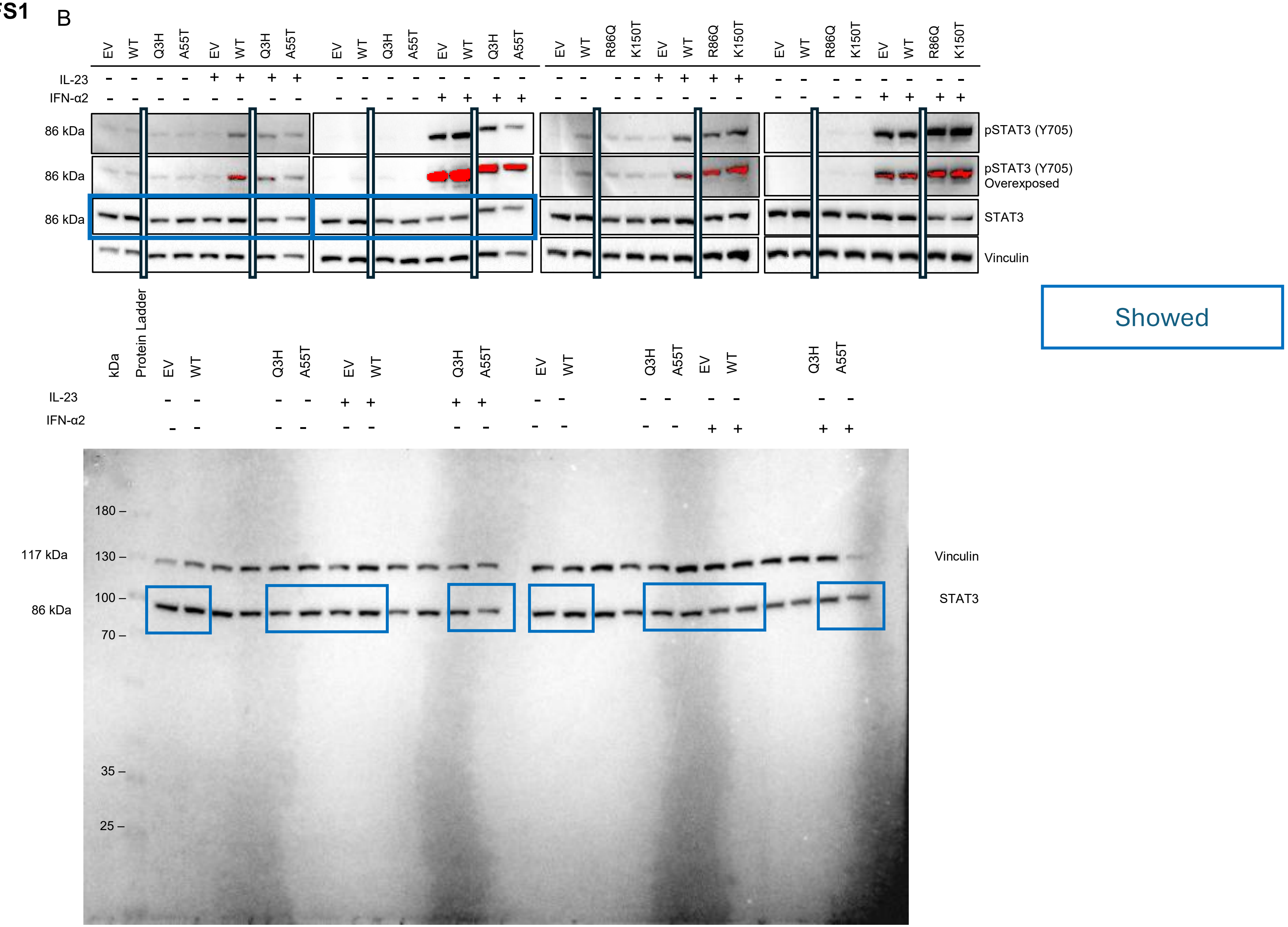

The ladder image was merged with the original image from the figure to visualize protein size

SourceDataFS1

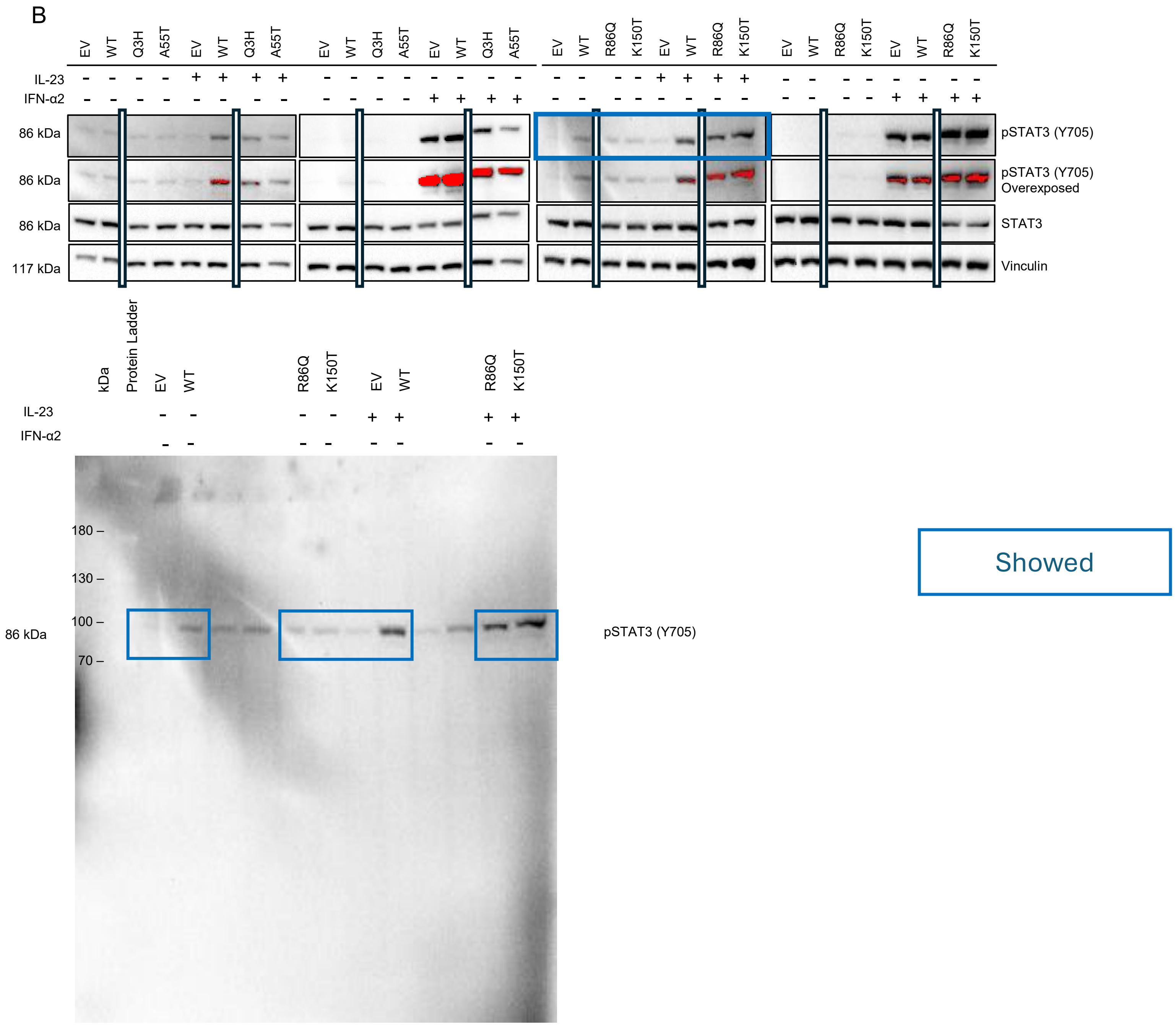

The ladder image was merged with the original image from the figure to visualize protein size

SourceDataFS1

B

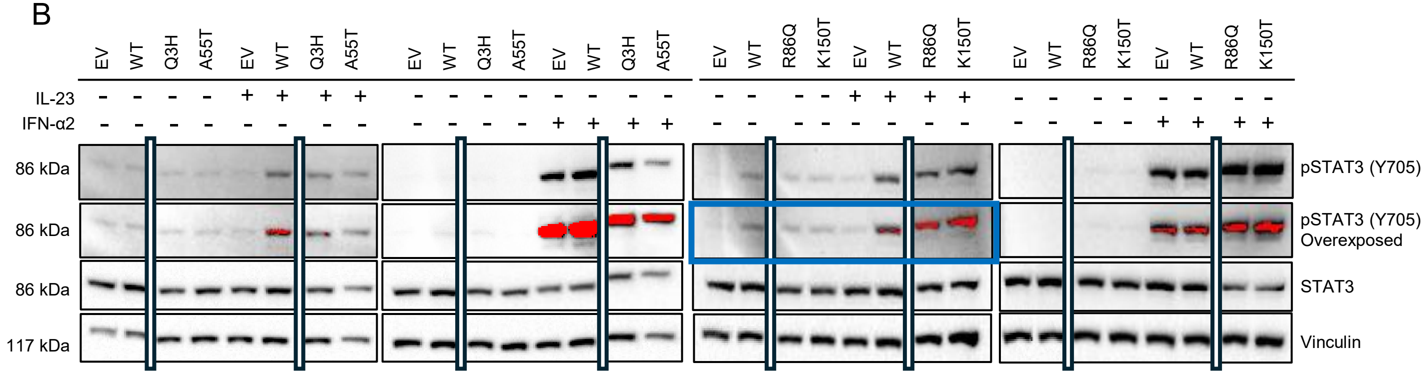

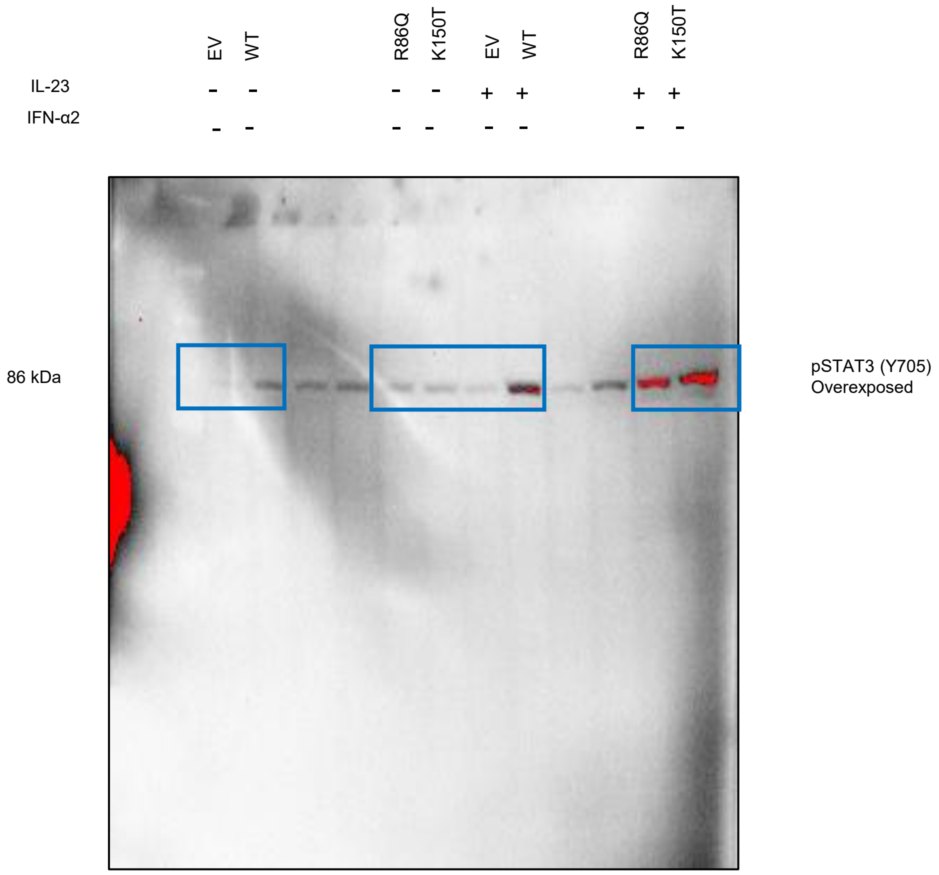

Showed

SourceDataFS1

B

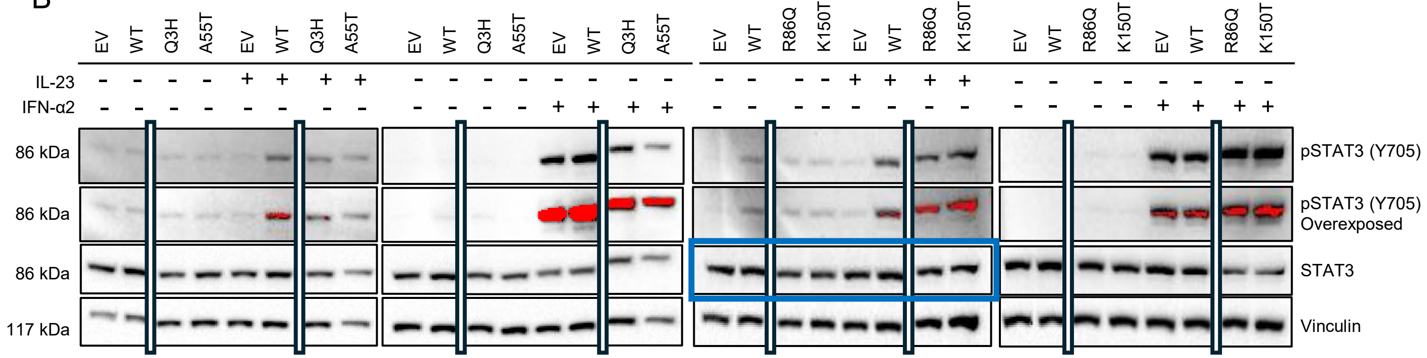

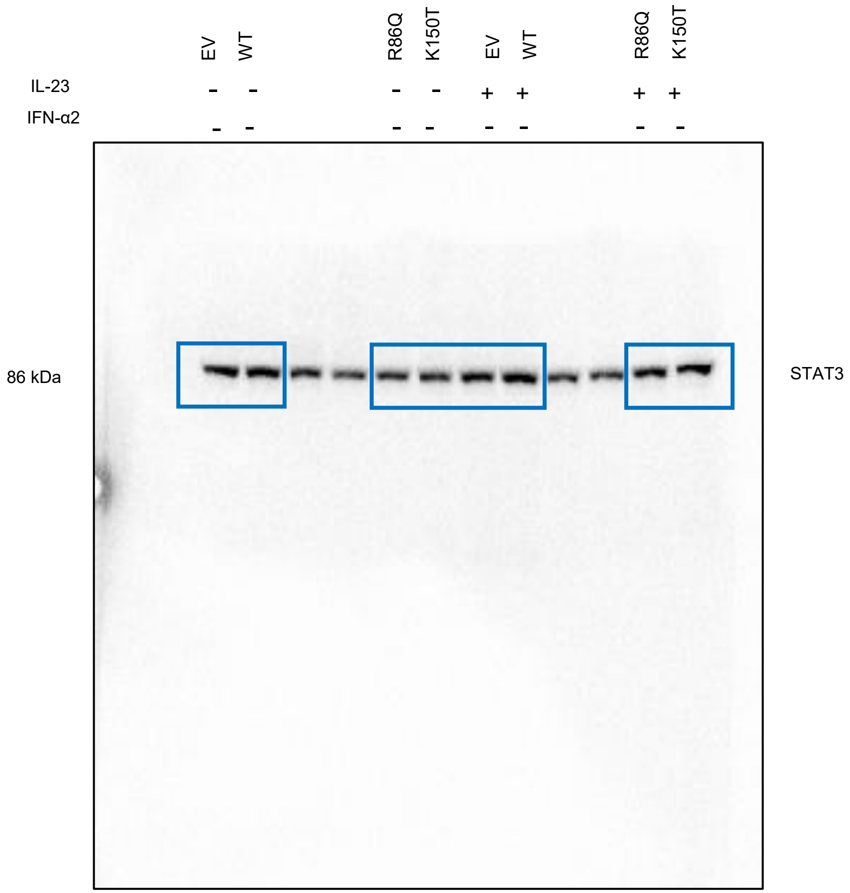

Showed

SourceDataFS1

B

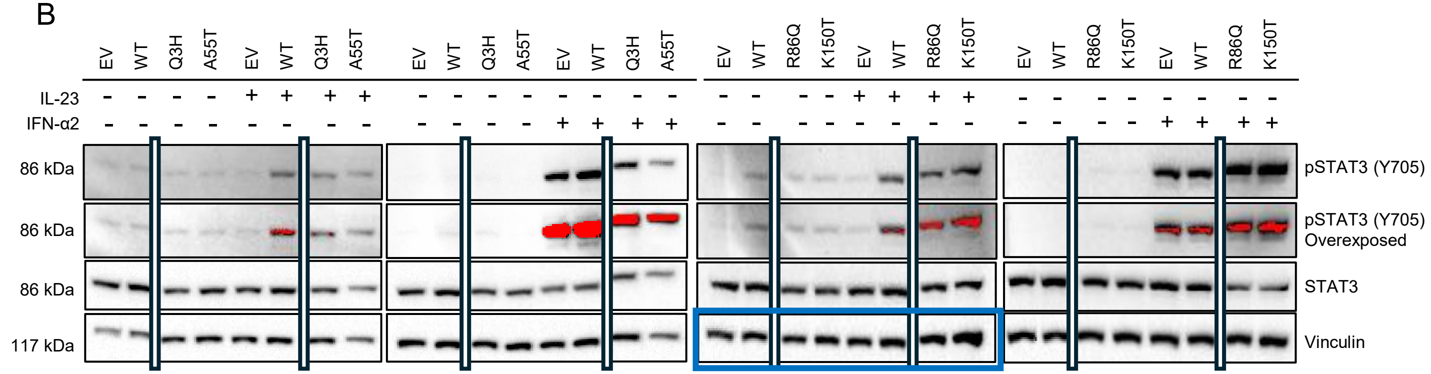

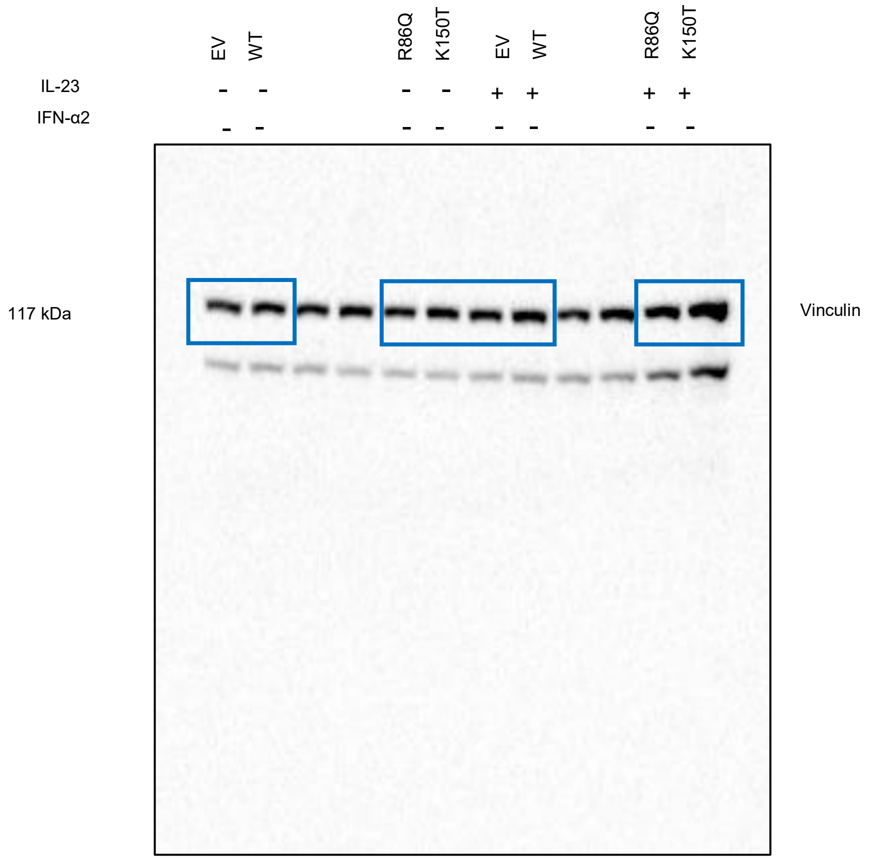

Showed

SourceDataFS1

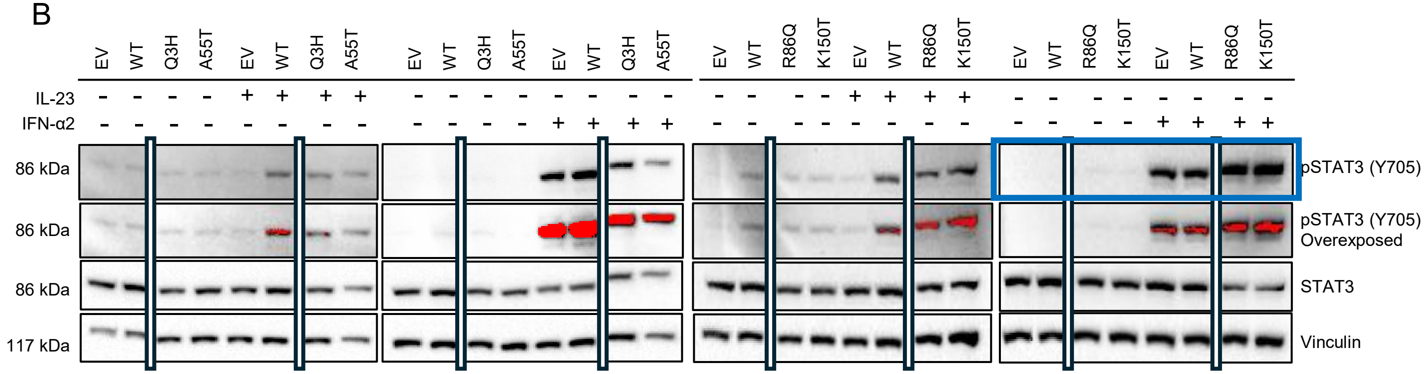

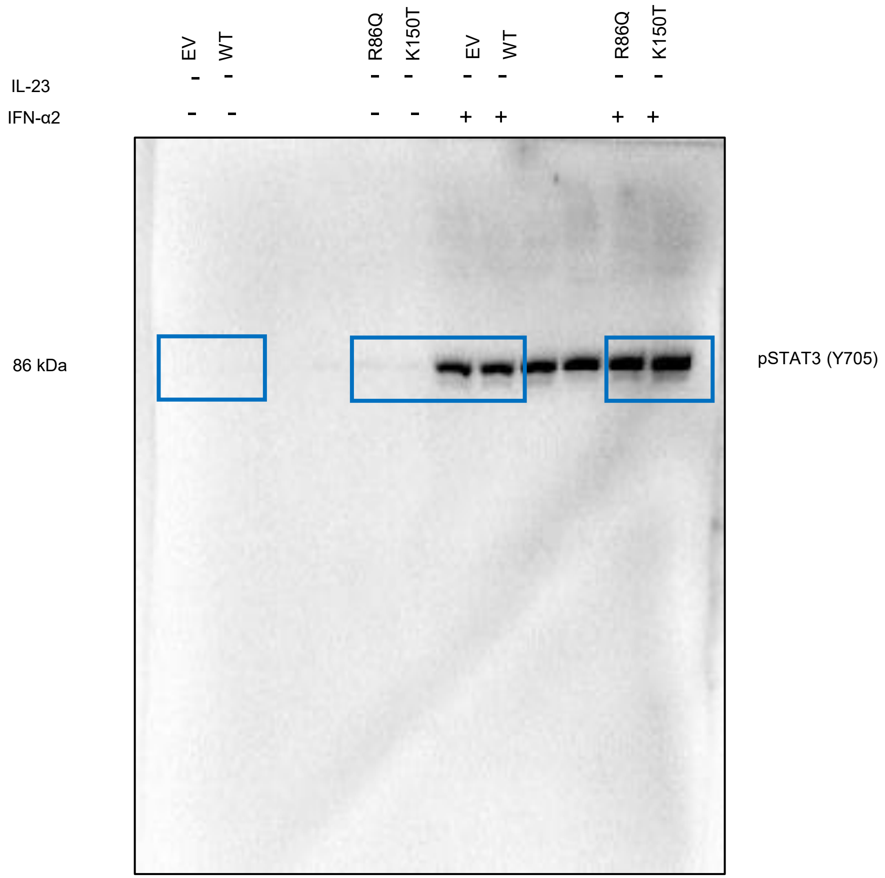

Showed

SourceDataFS1

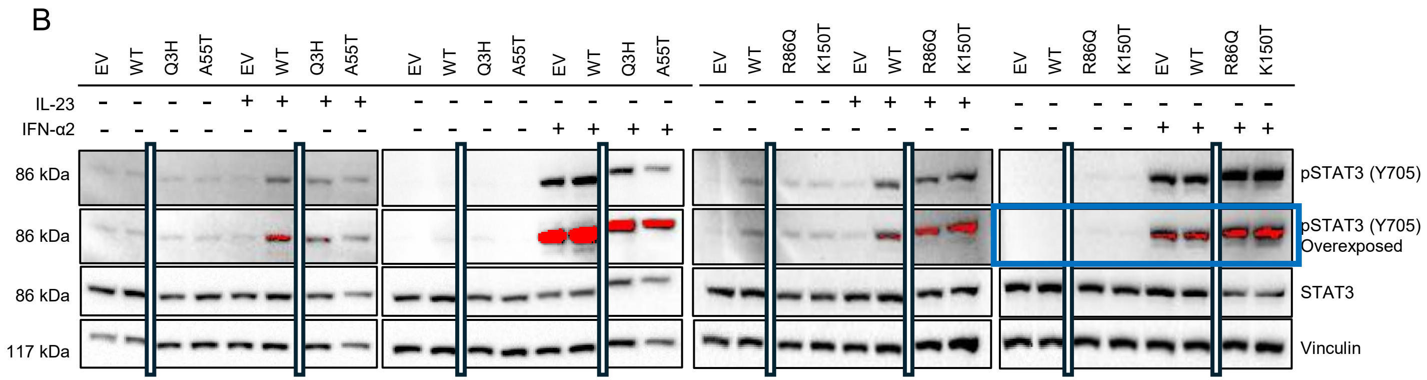

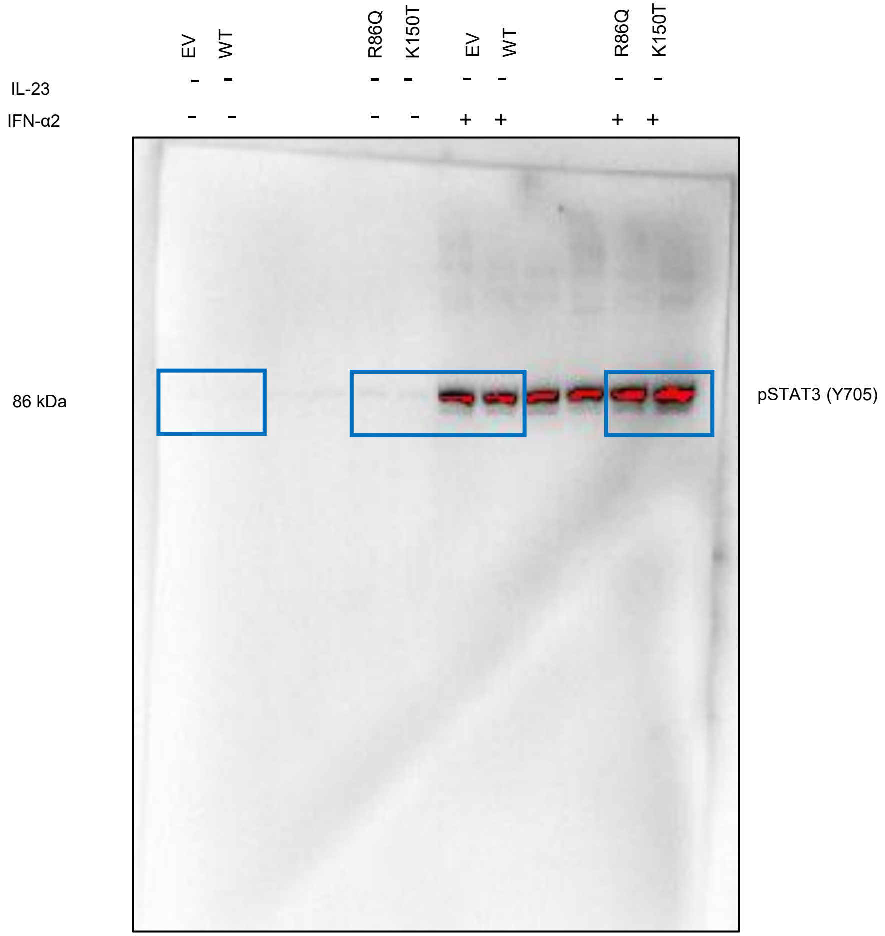

Showed

SourceDataFS1

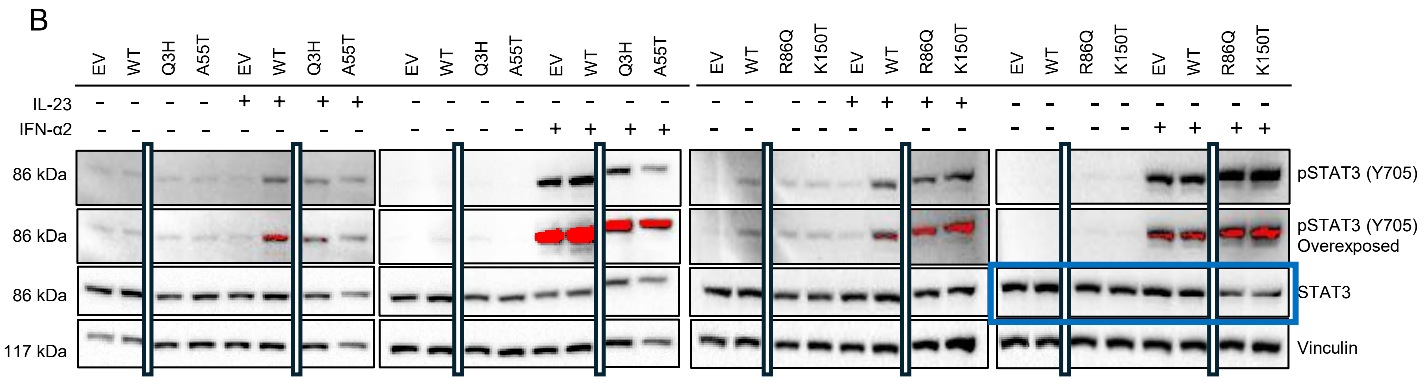

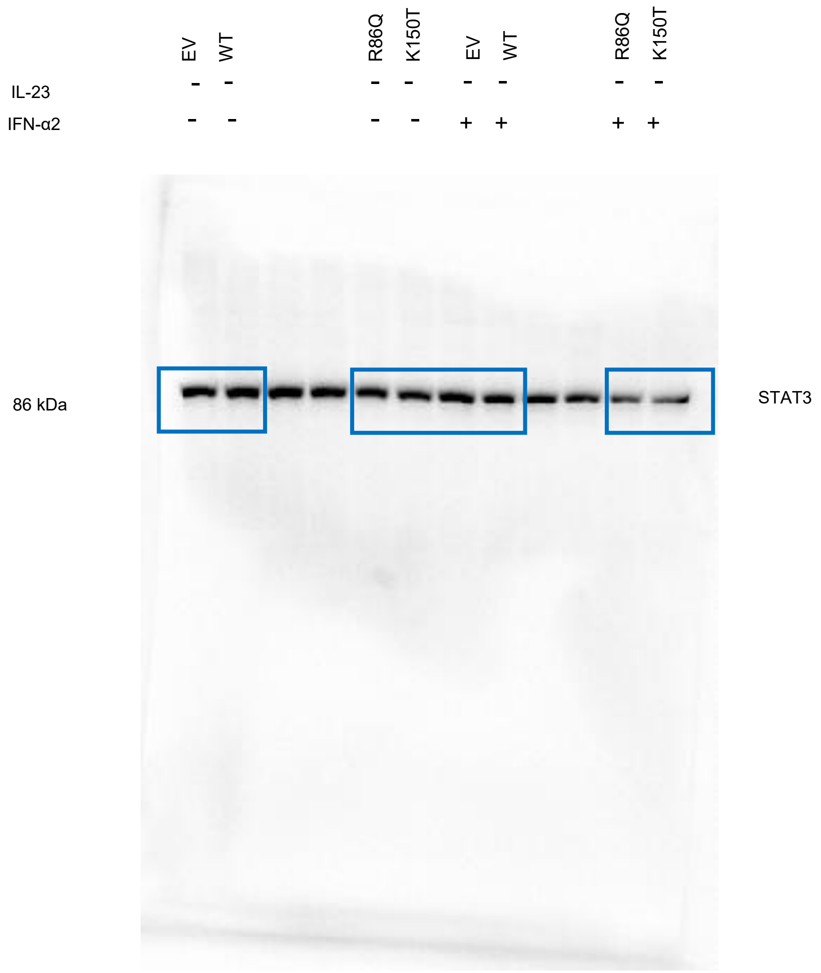

Showed

SourceDataFS1

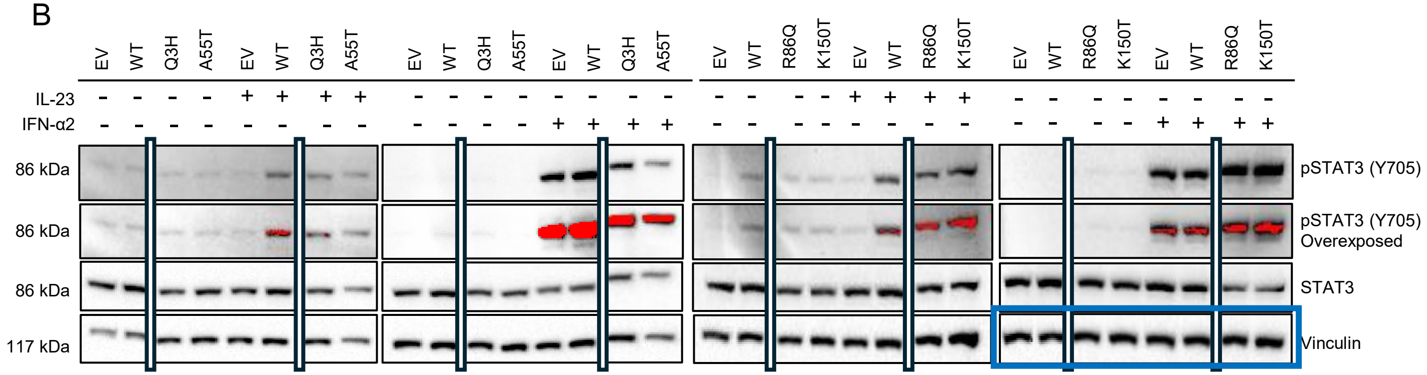

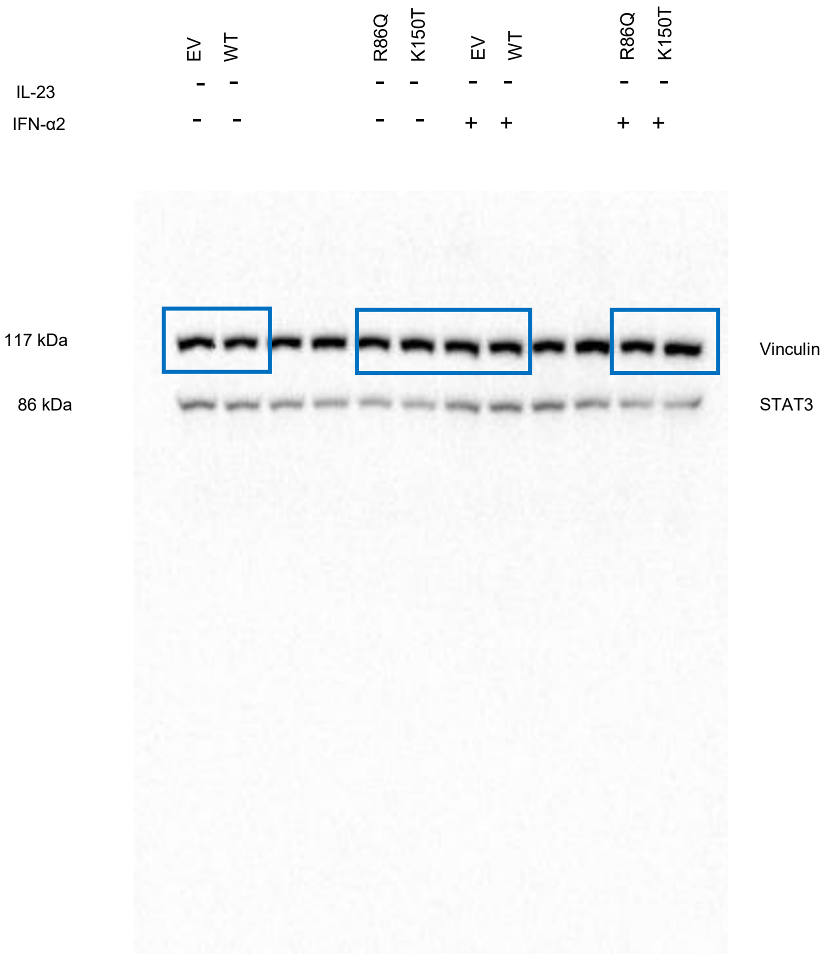

Showed

SourceDataFS1

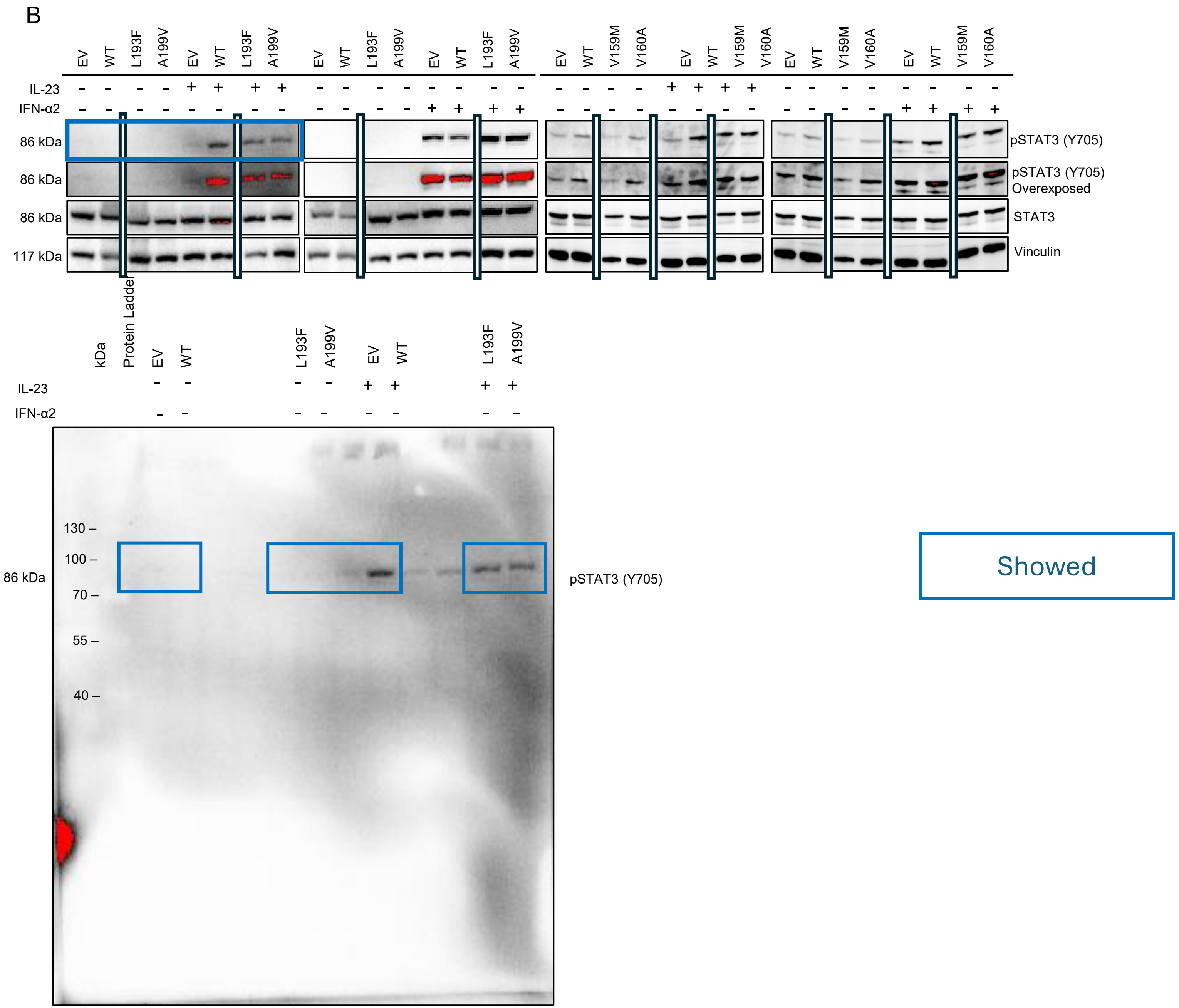

SourceDataFS1

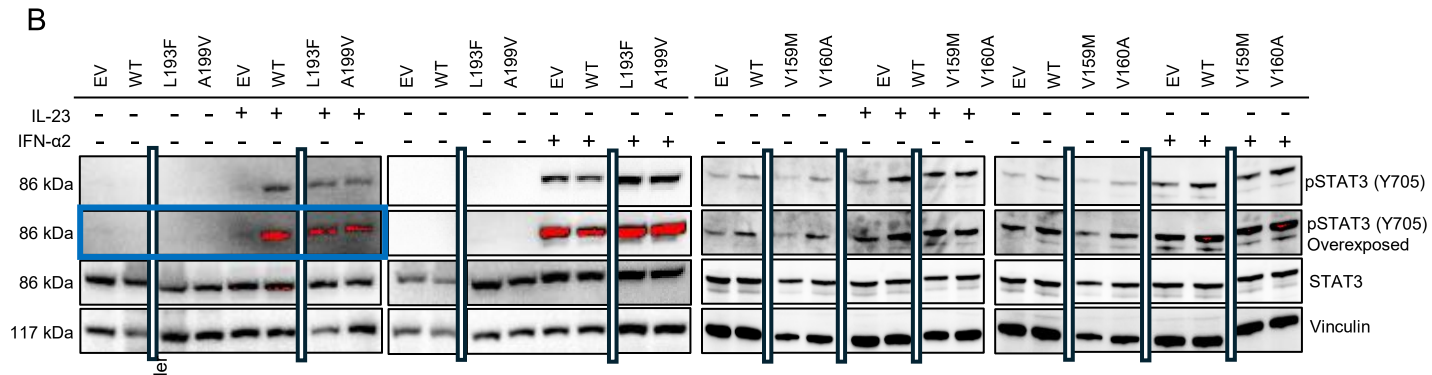

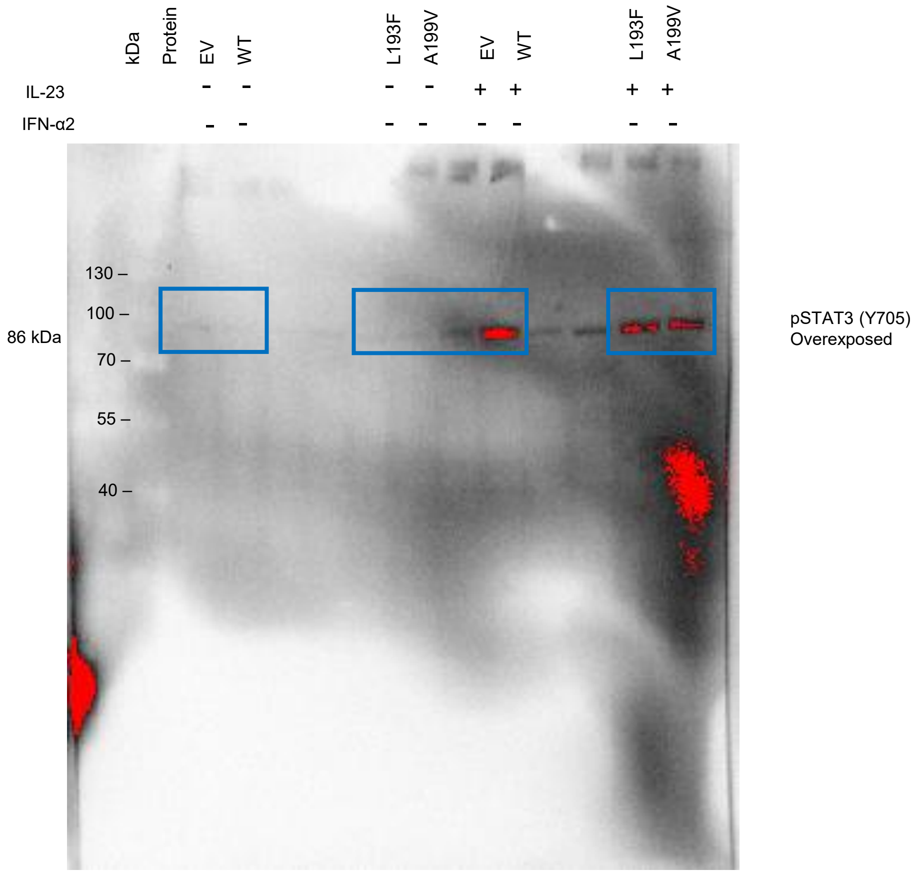

Showed

SourceDataFS1

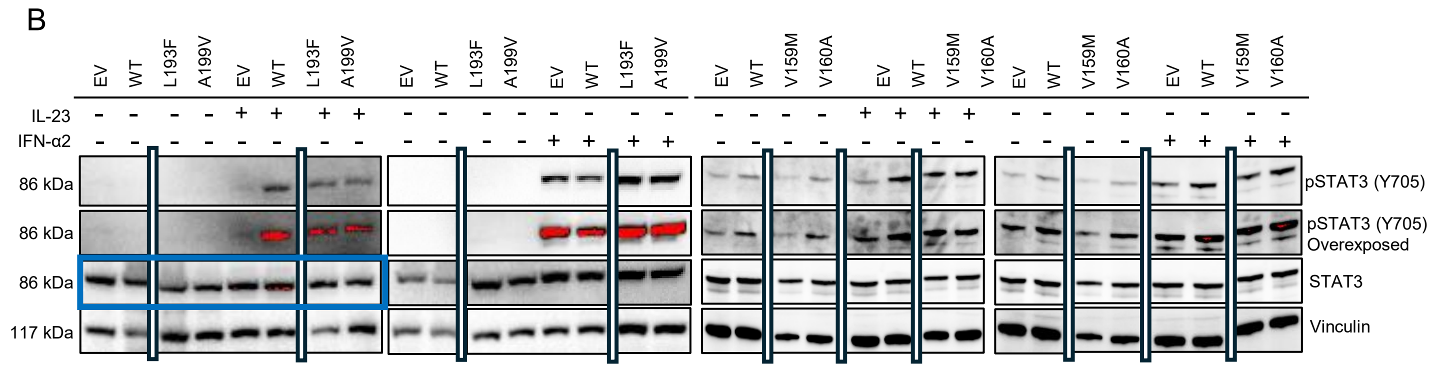

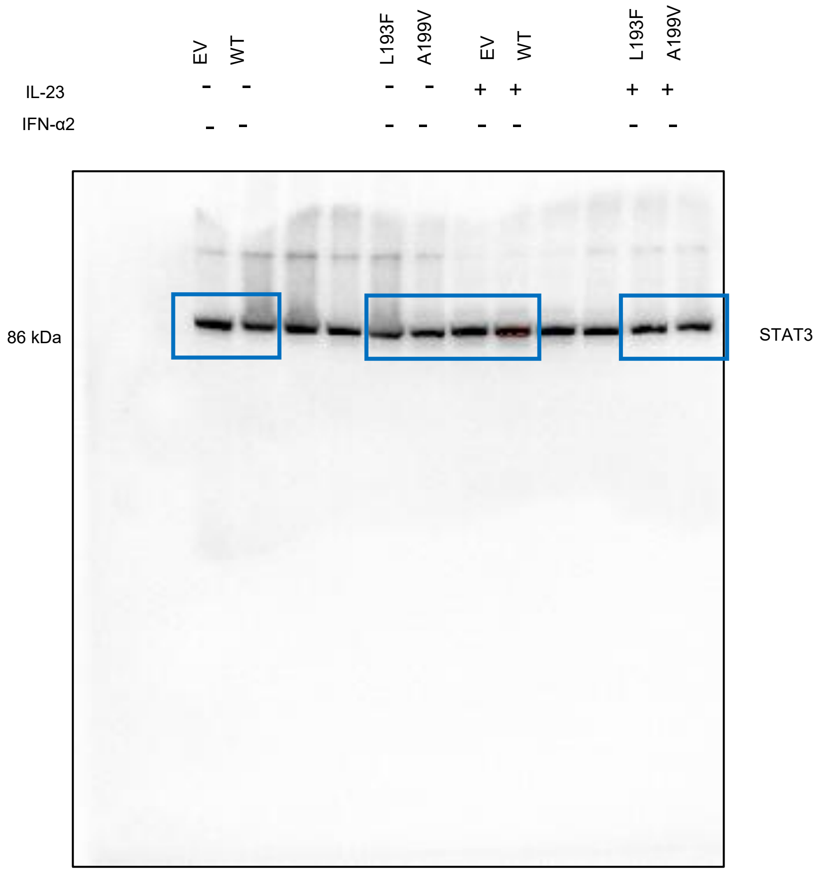

Showed

SourceDataFS1

B

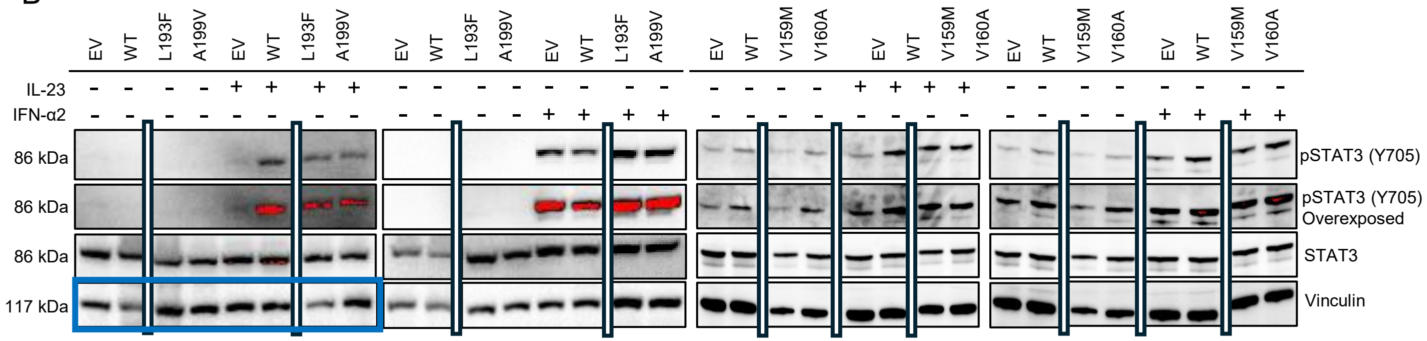

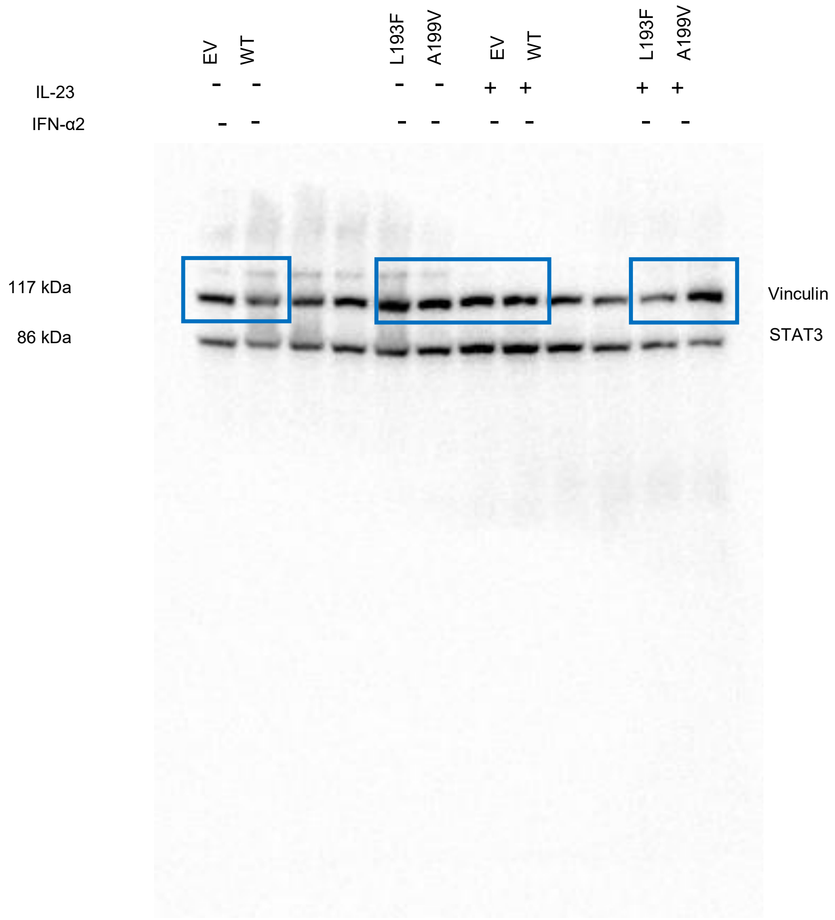

Showed

SourceDataFS1

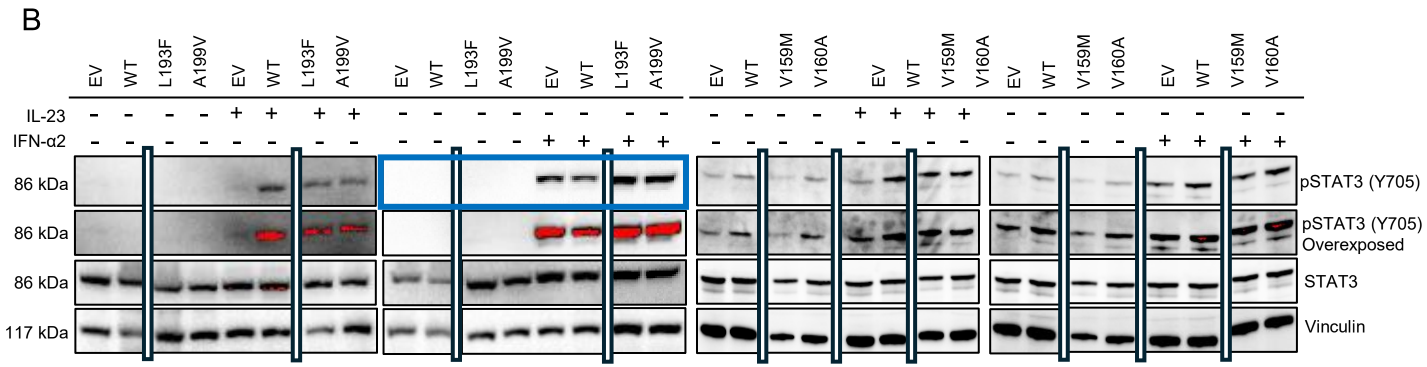

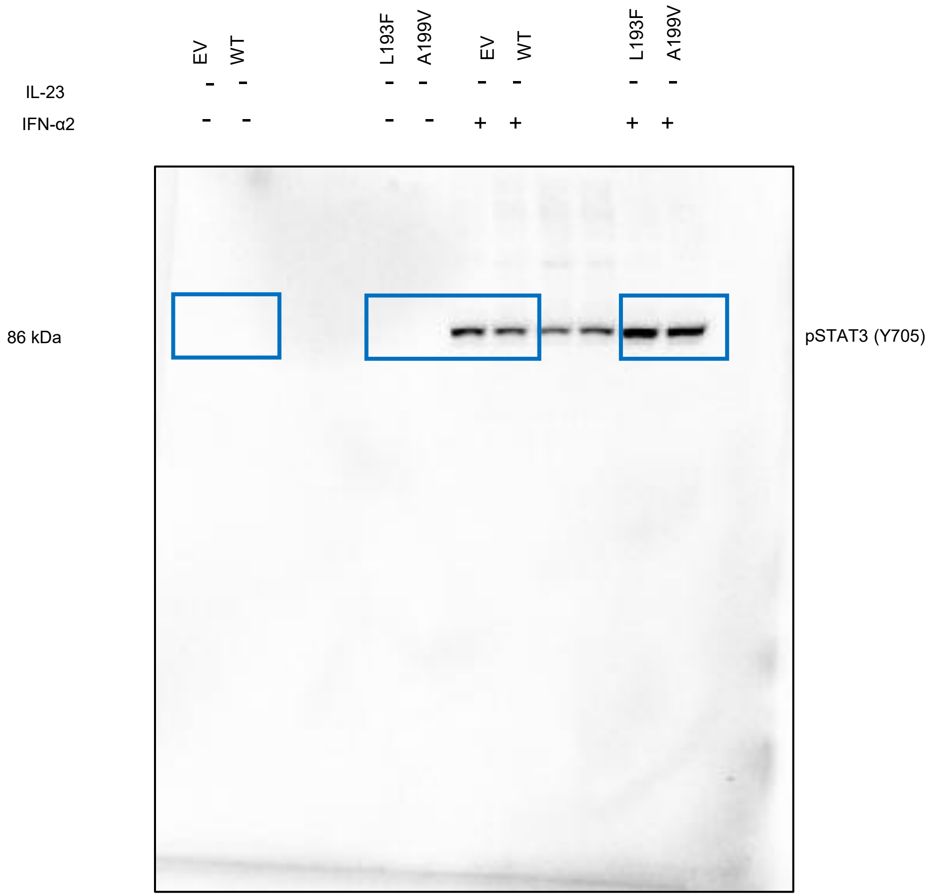

Showed

SourceDataFS1

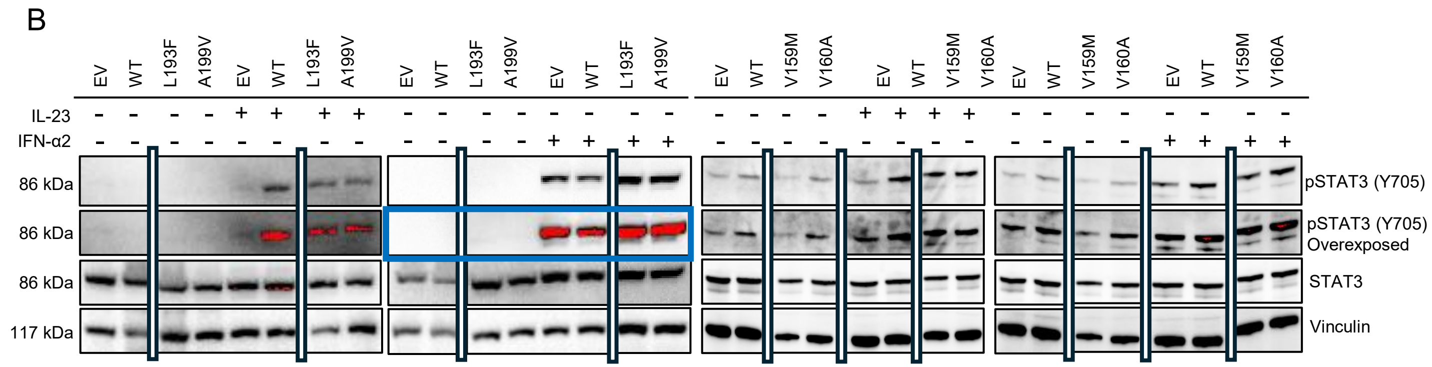

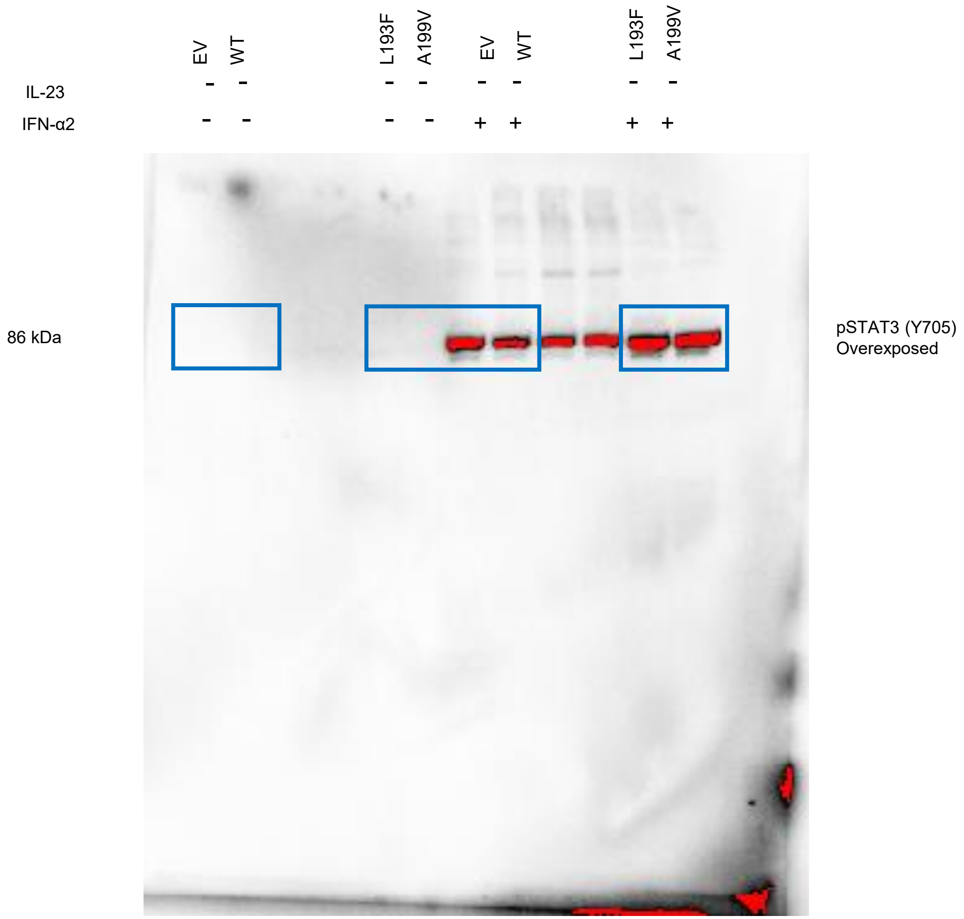

Showed

SourceDataFS1

B

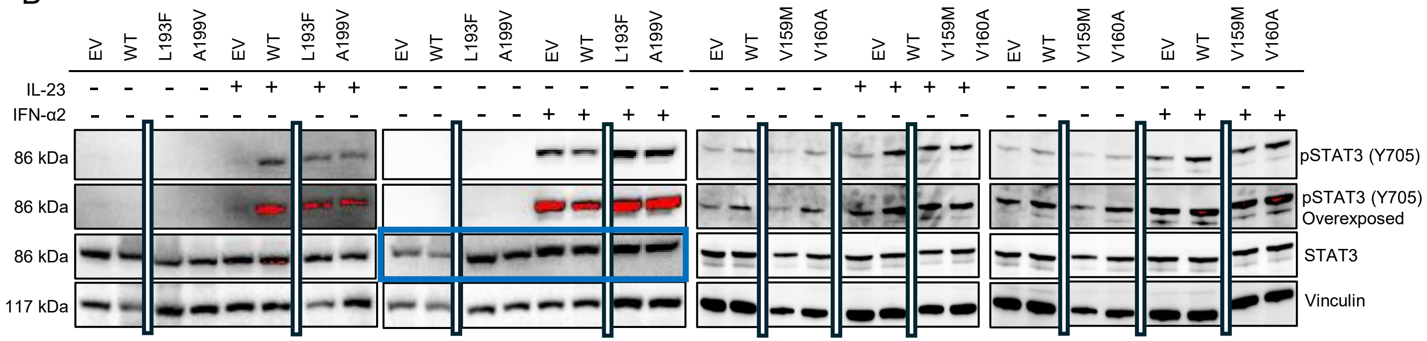

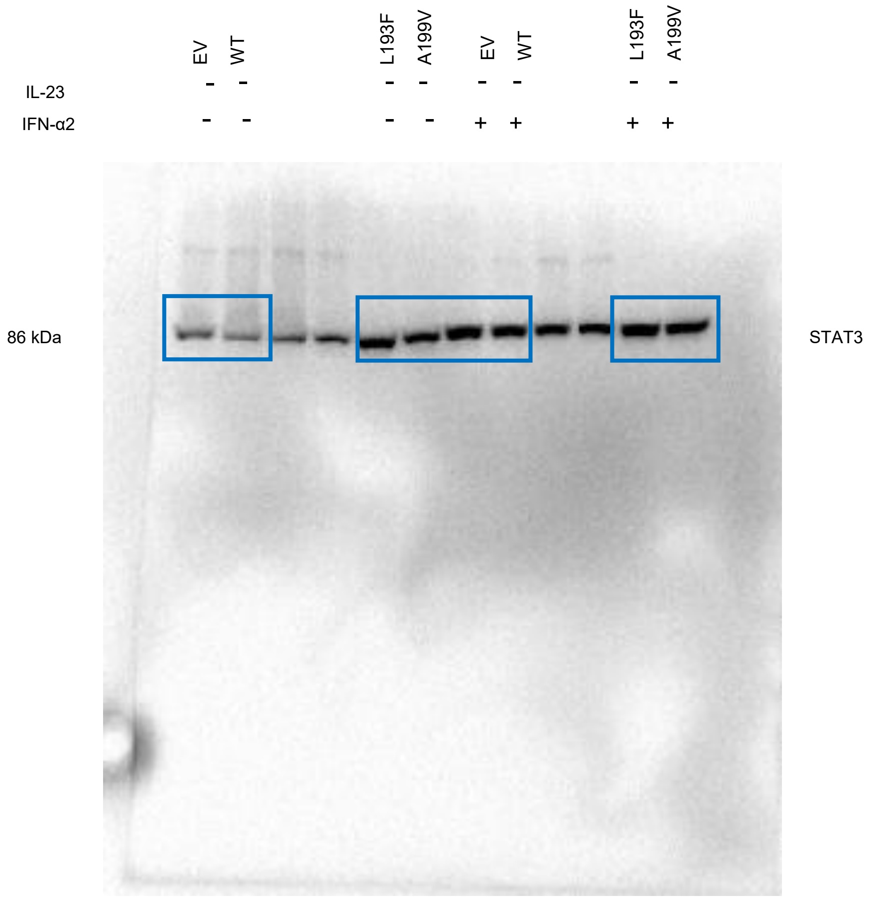

Showed

SourceDataFS1

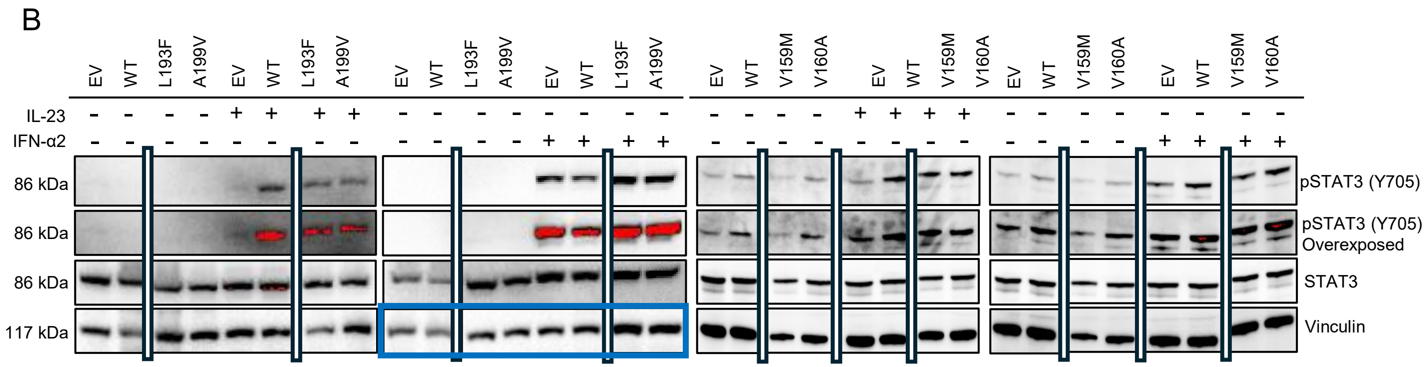

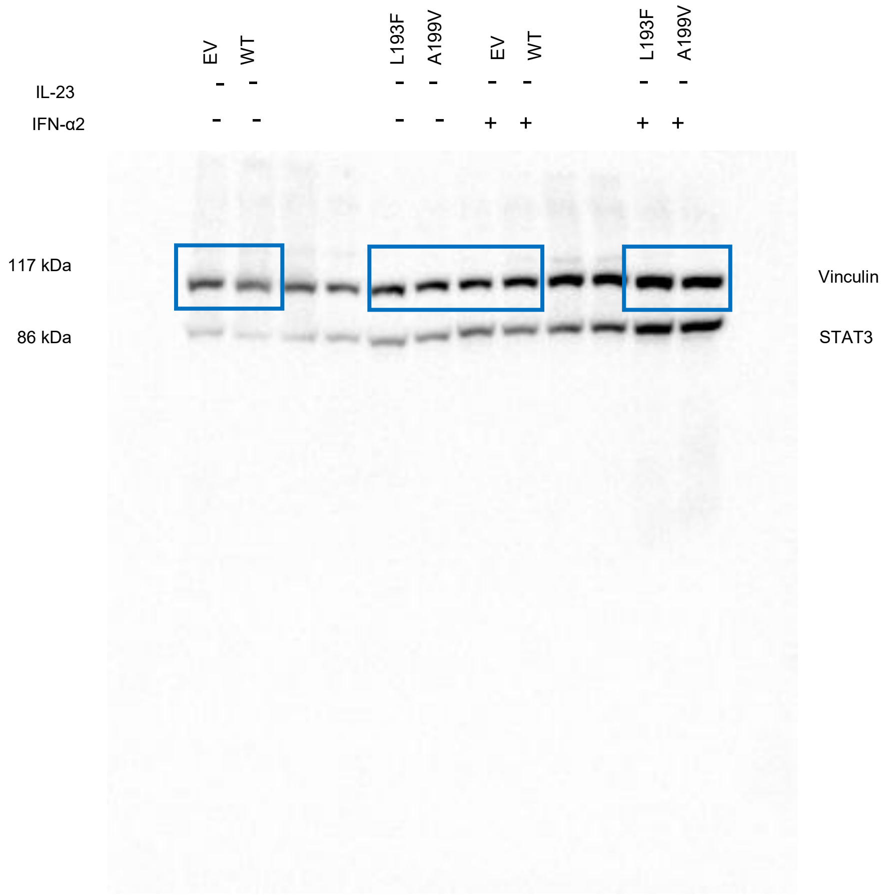

Showed

SourceDataFS1

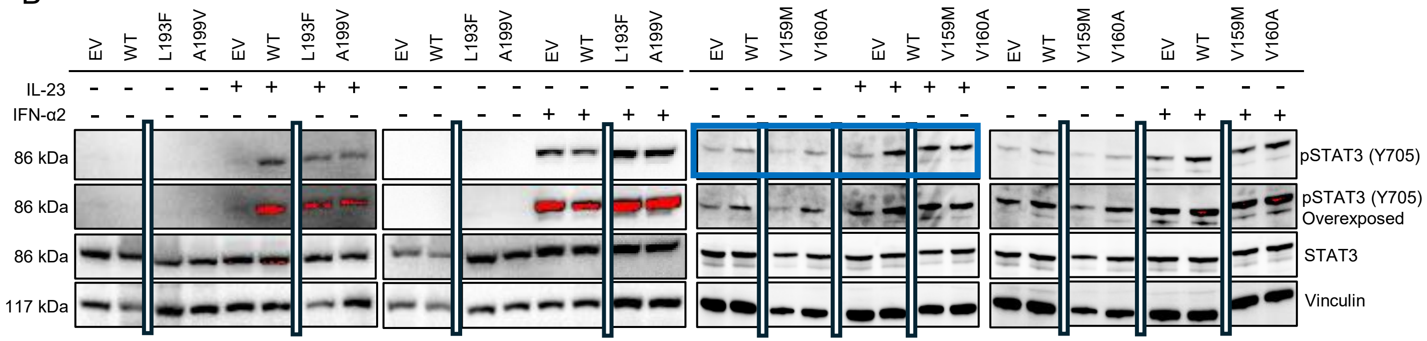

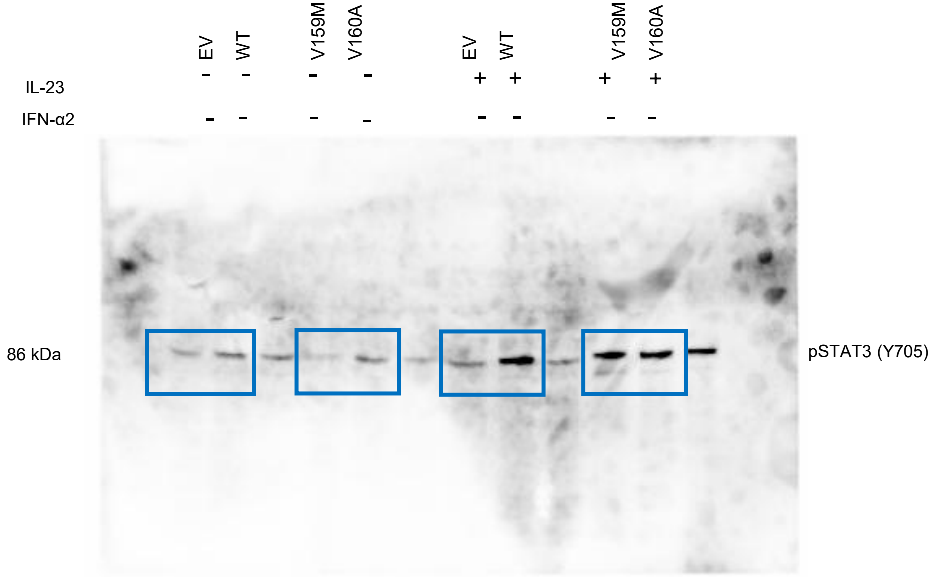

Showed

SourceDataFS1

B

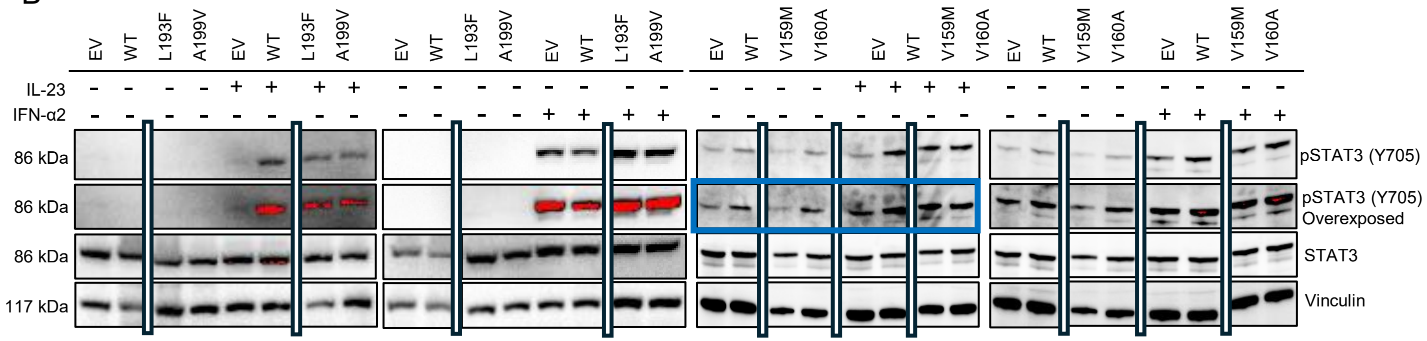

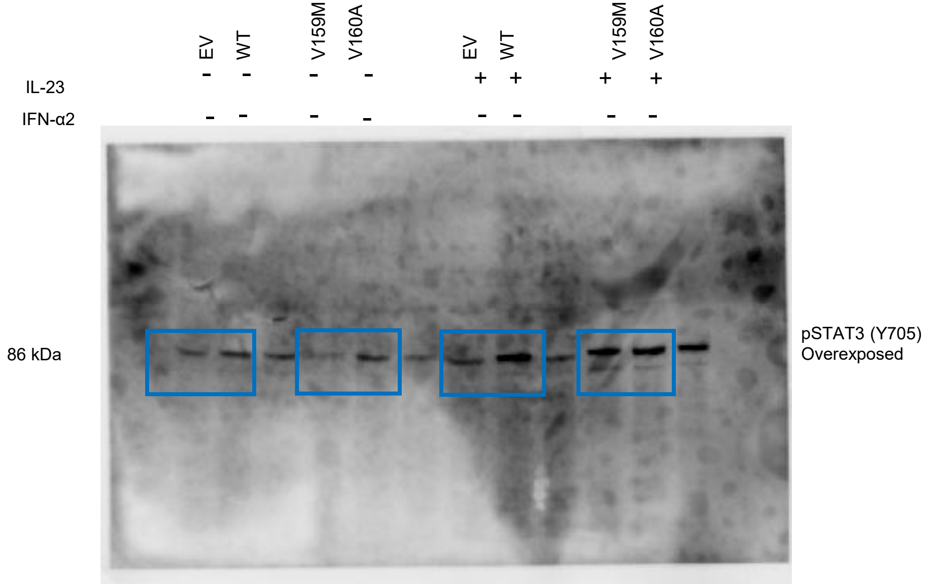

Showed

SourceDataFS1

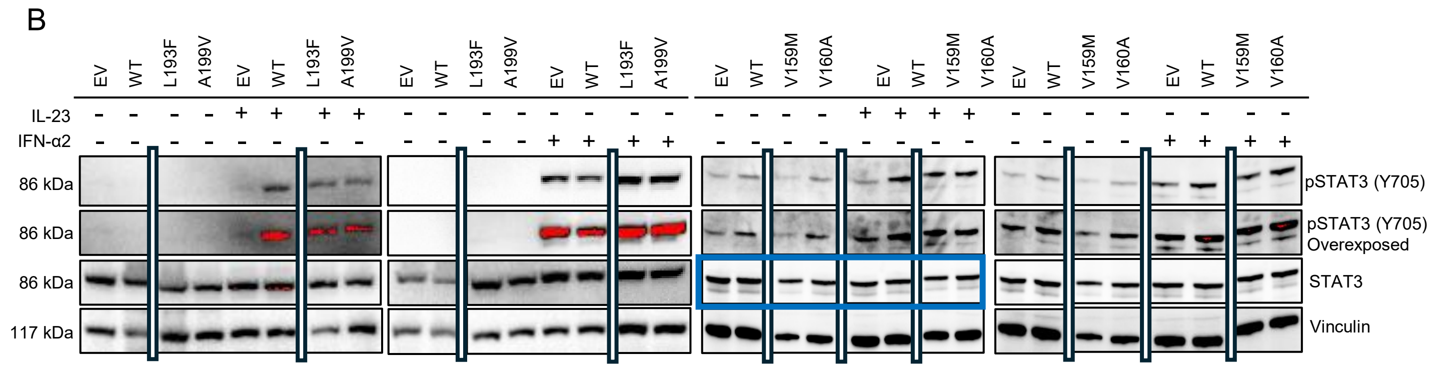

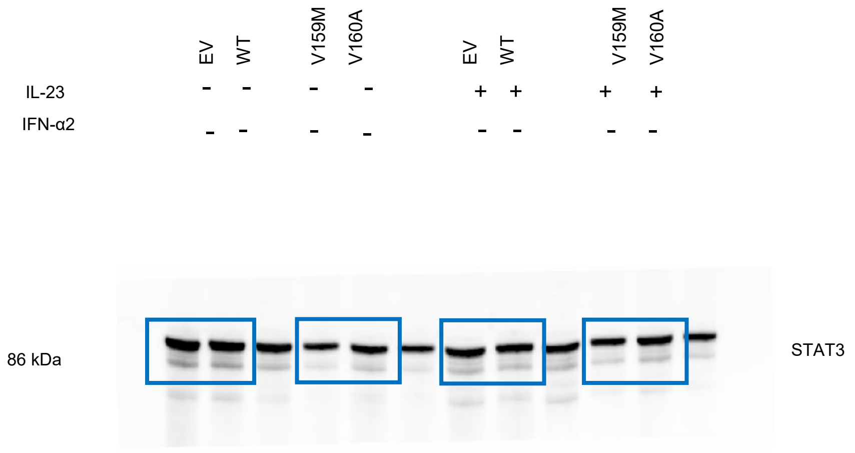

Showed

SourceDataFS1

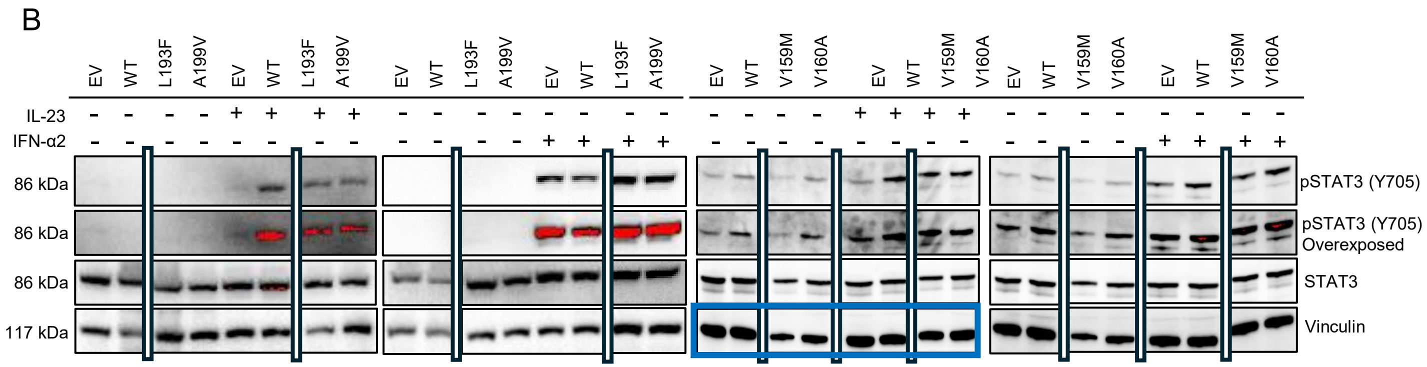

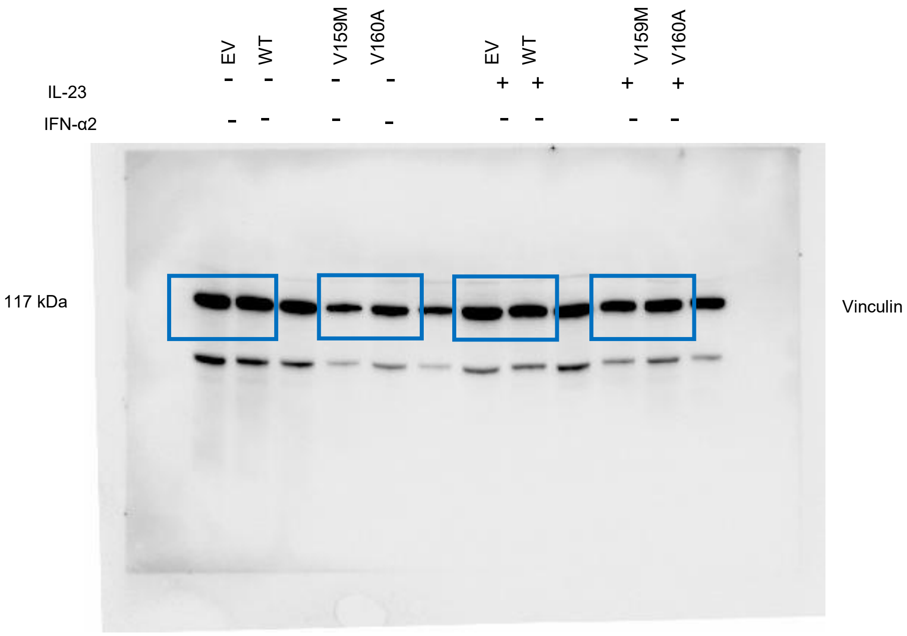

Showed

SourceDataFS1

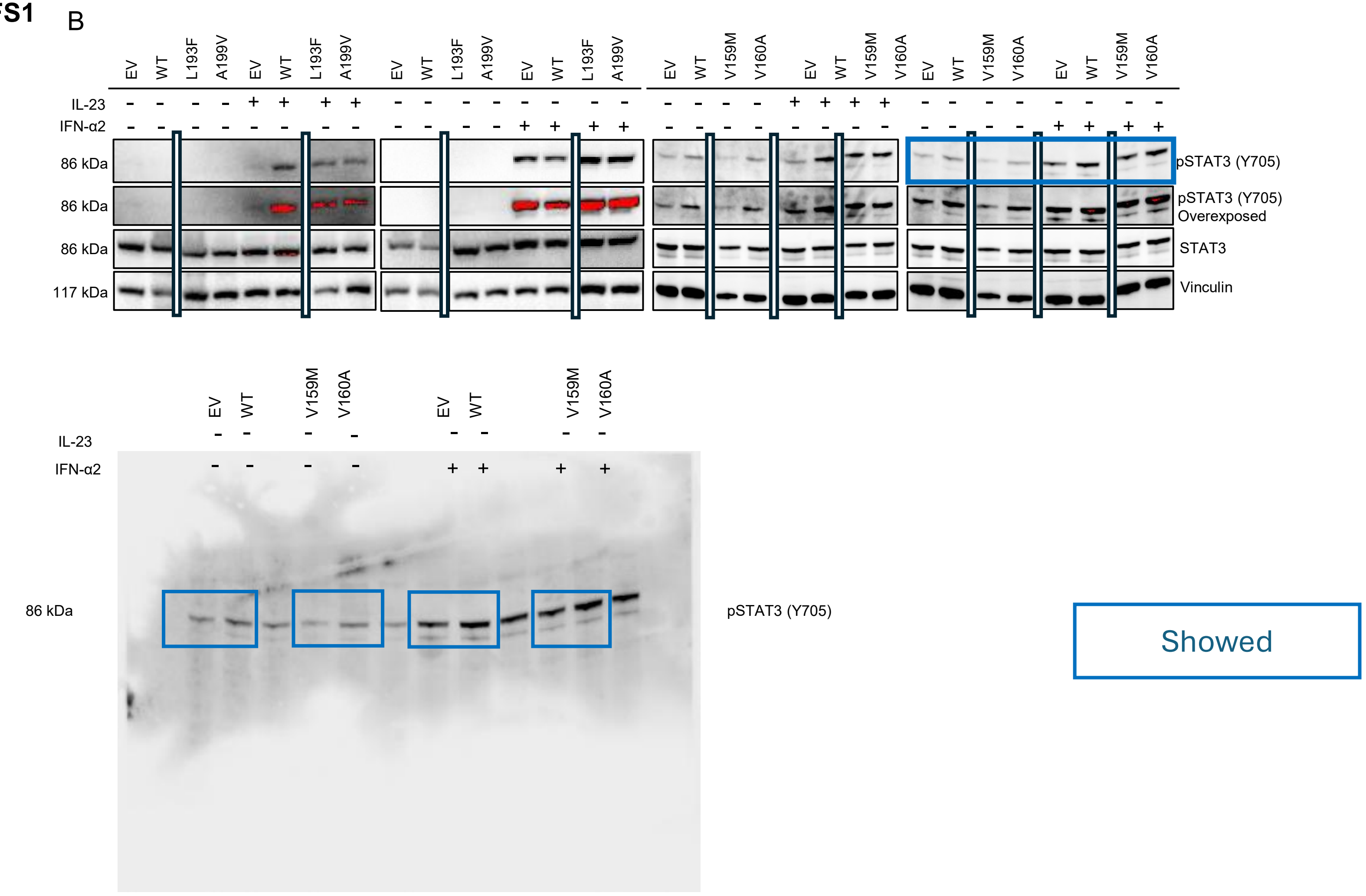

SourceDataFS1

B

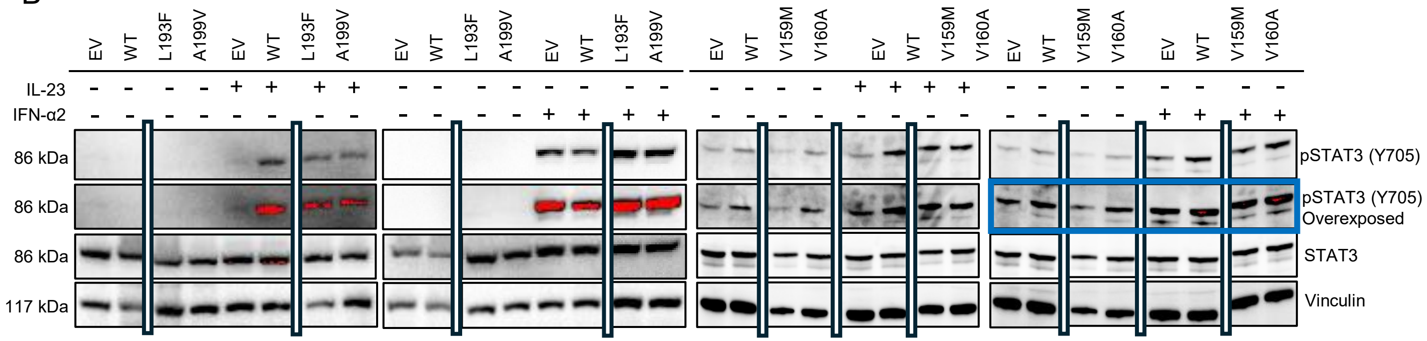

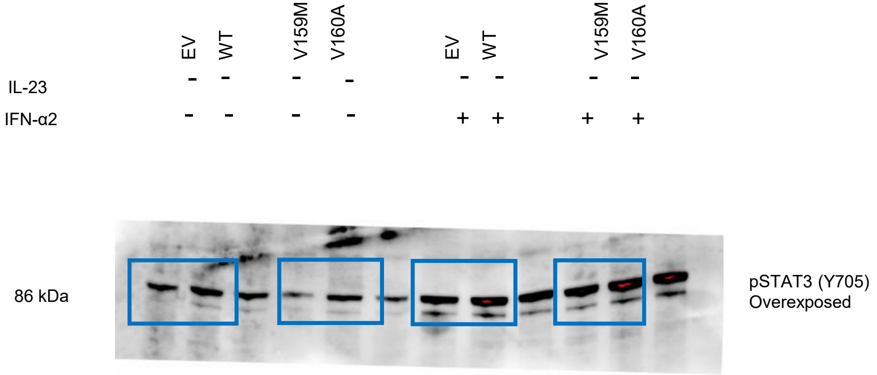

Showed

SourceDataFS1

B

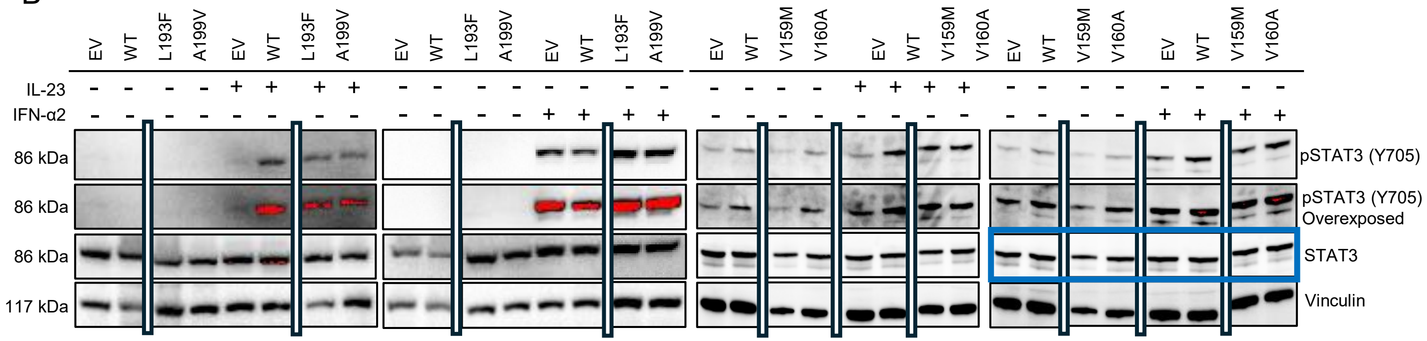

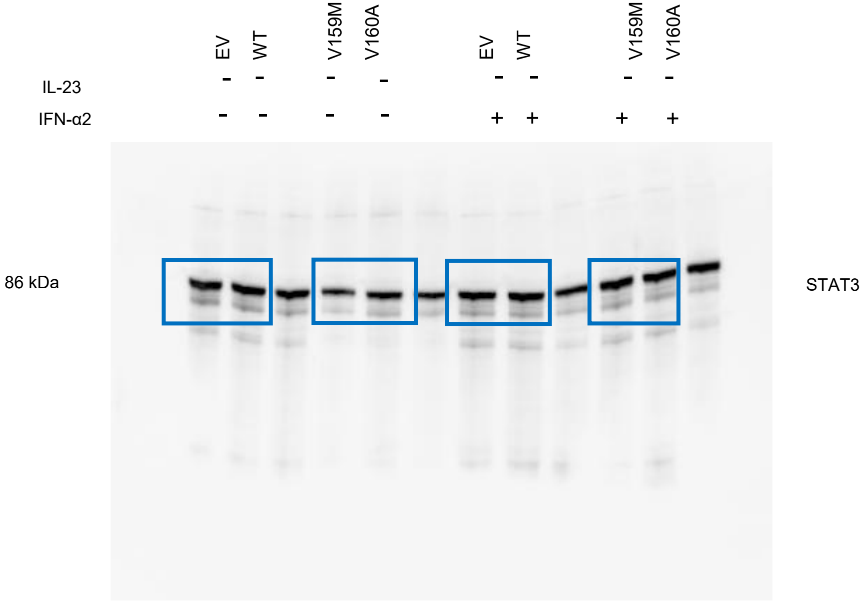

Showed

SourceDataFS1

B

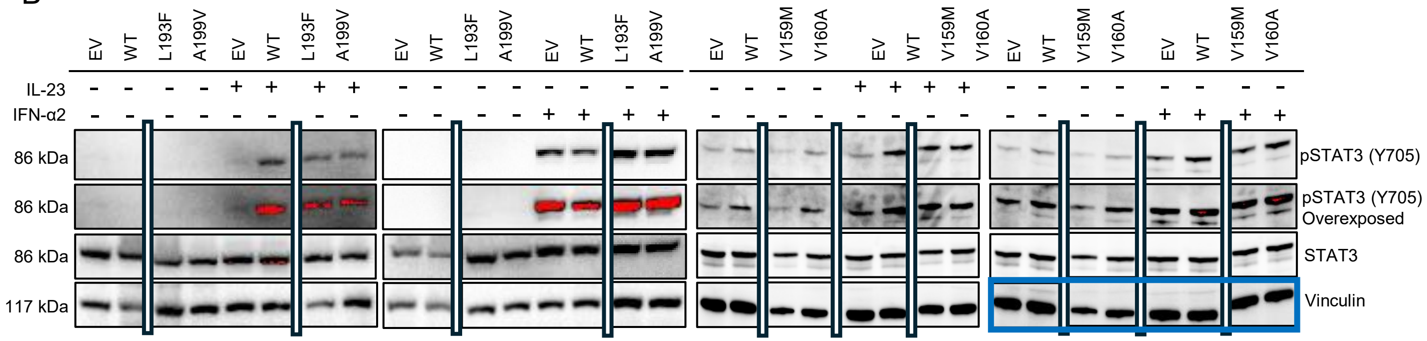

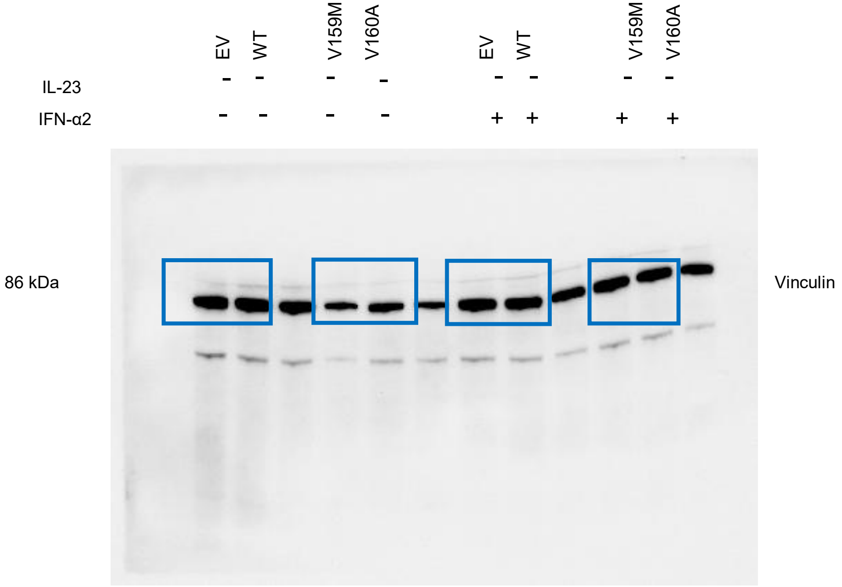

Showed

SourceDataFS1

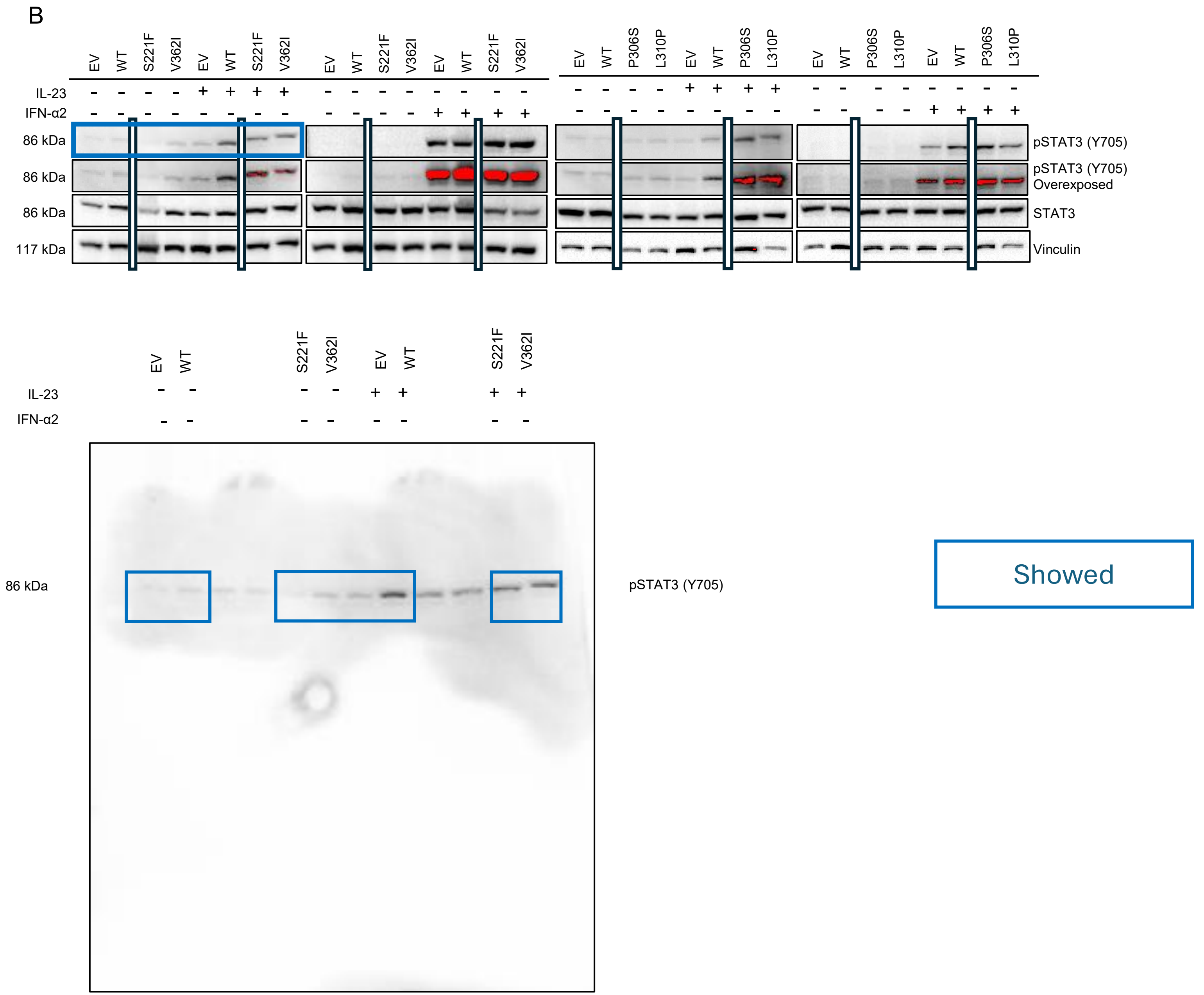

SourceDataFS1

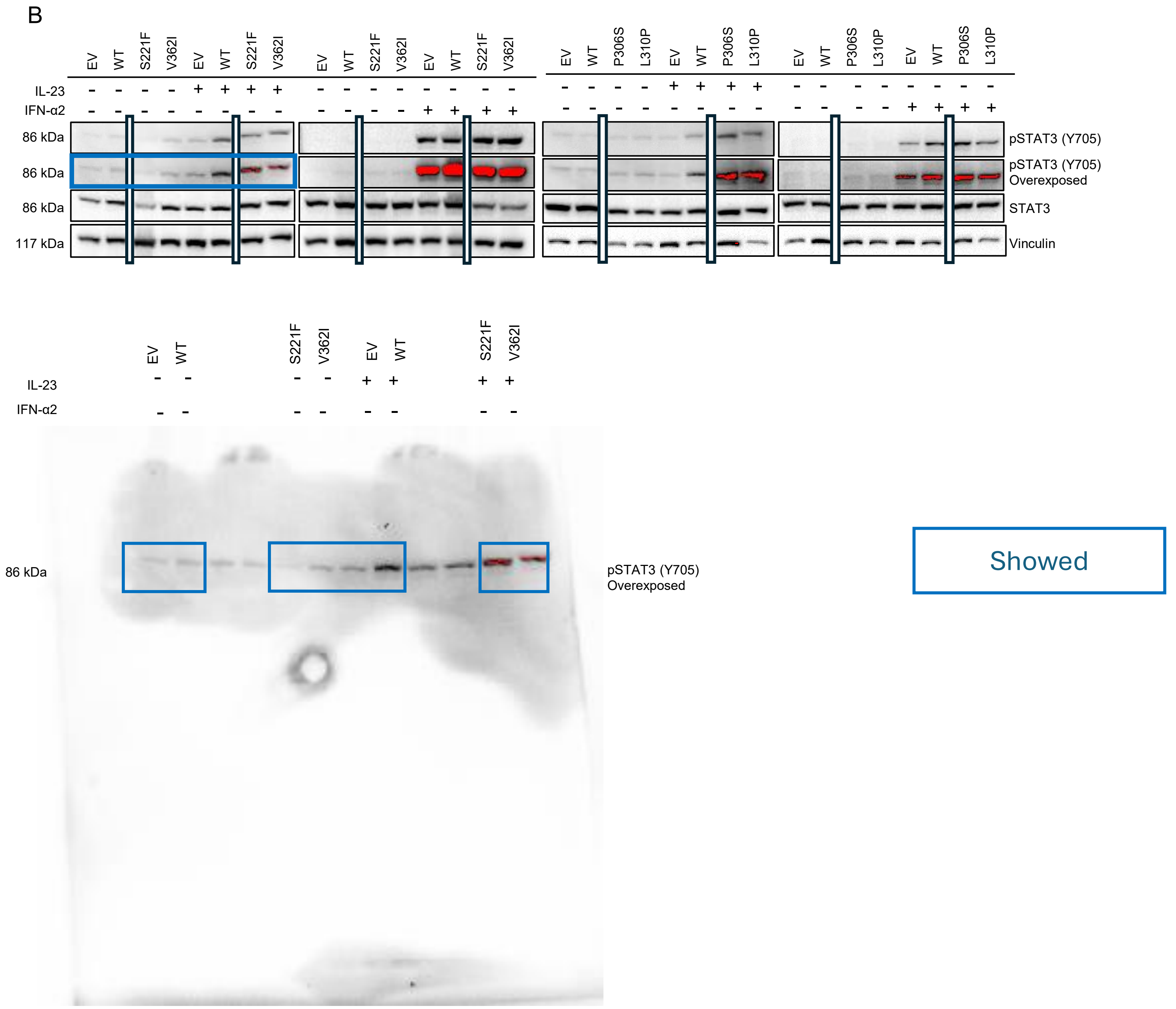

SourceDataFS1

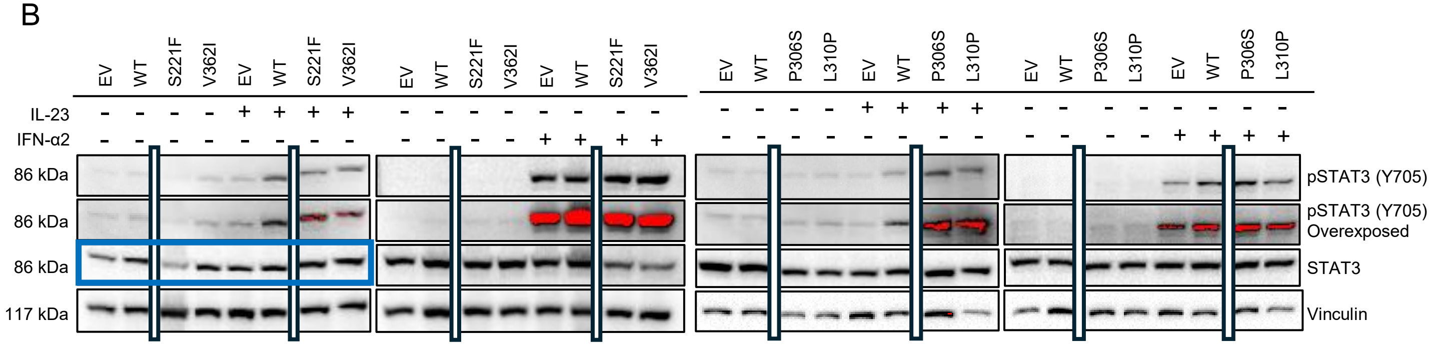

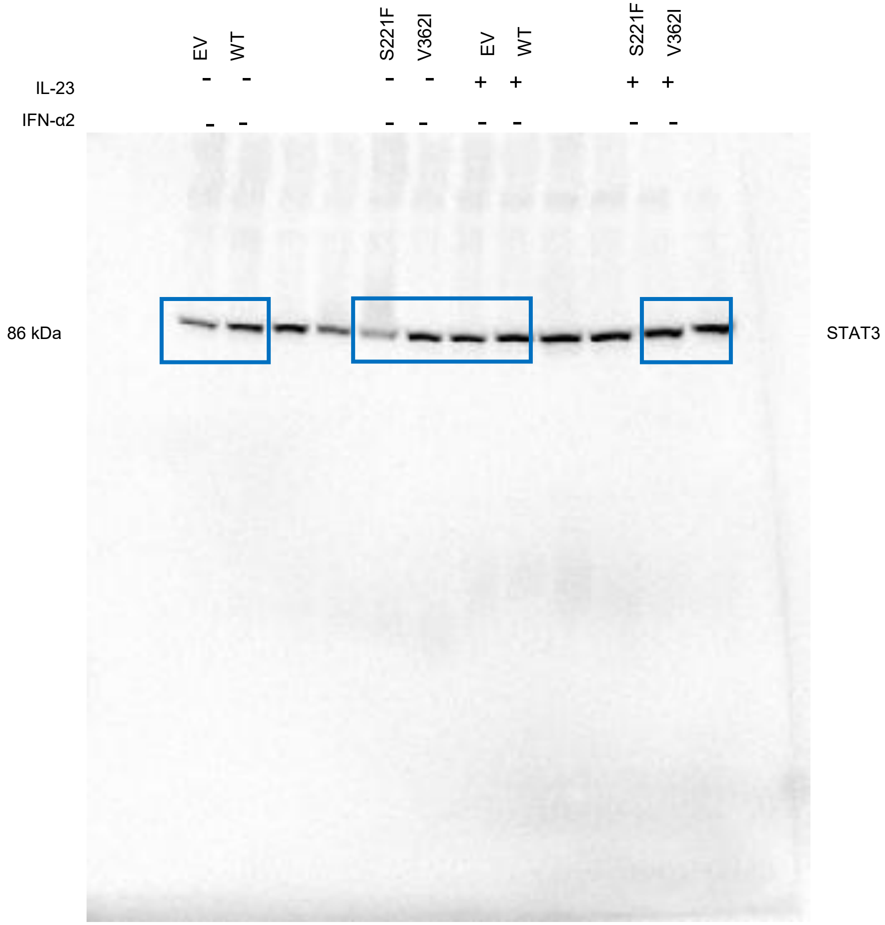

Showed

SourceDataFS1

B

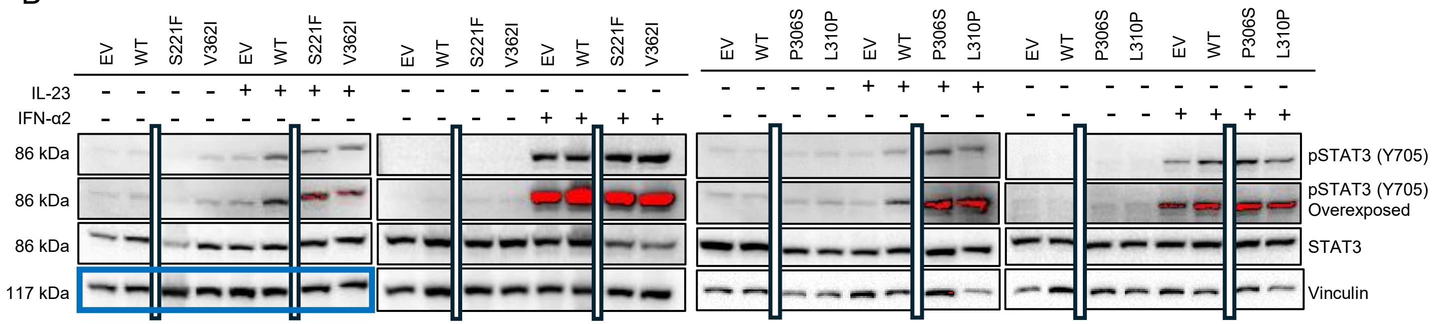

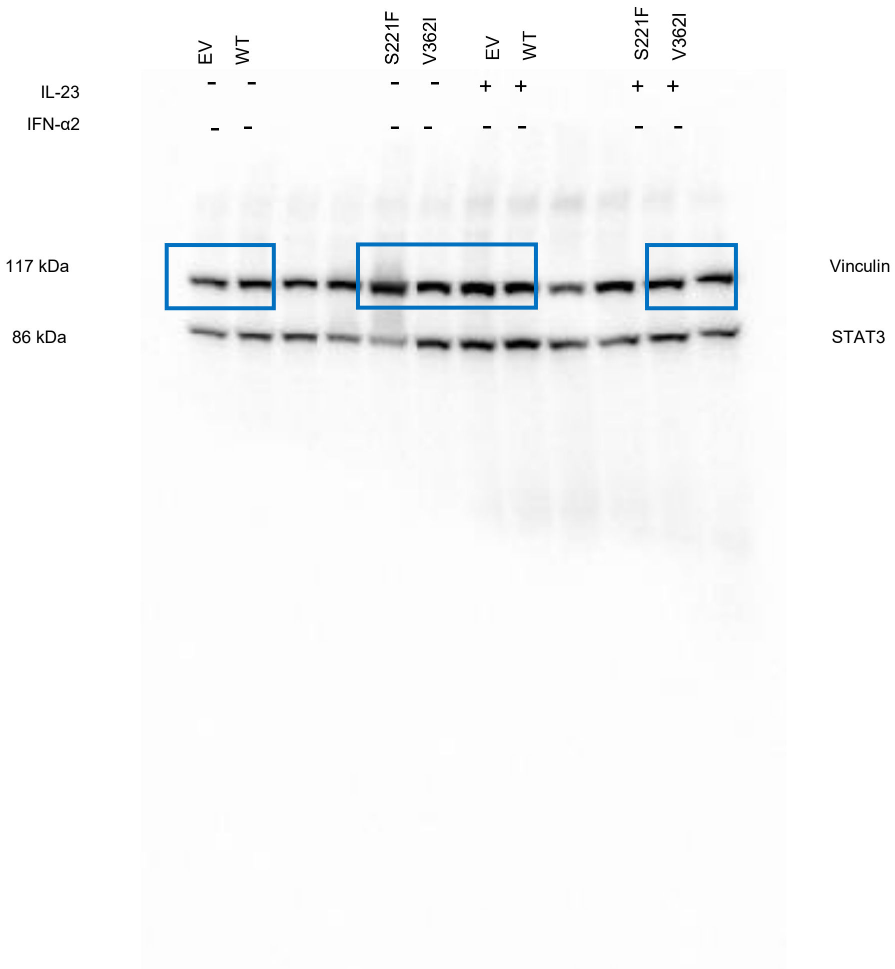

Showed

SourceDataFS1

B

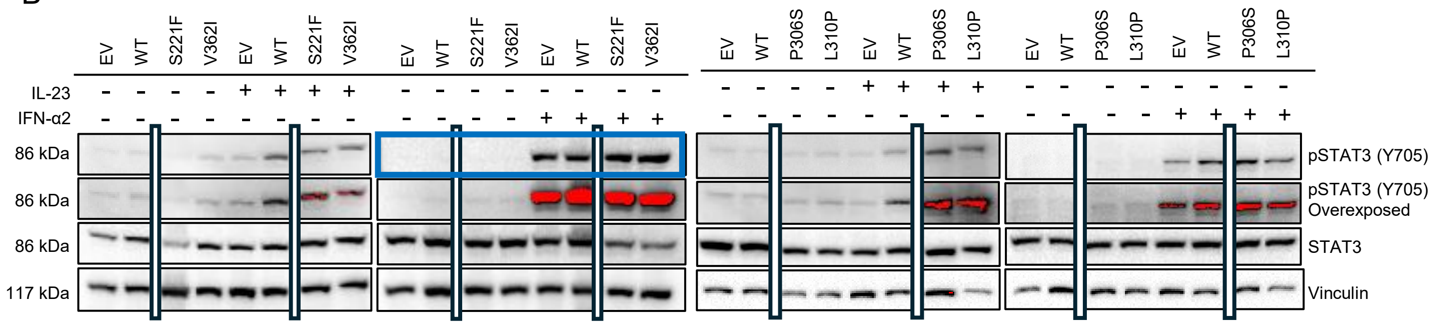

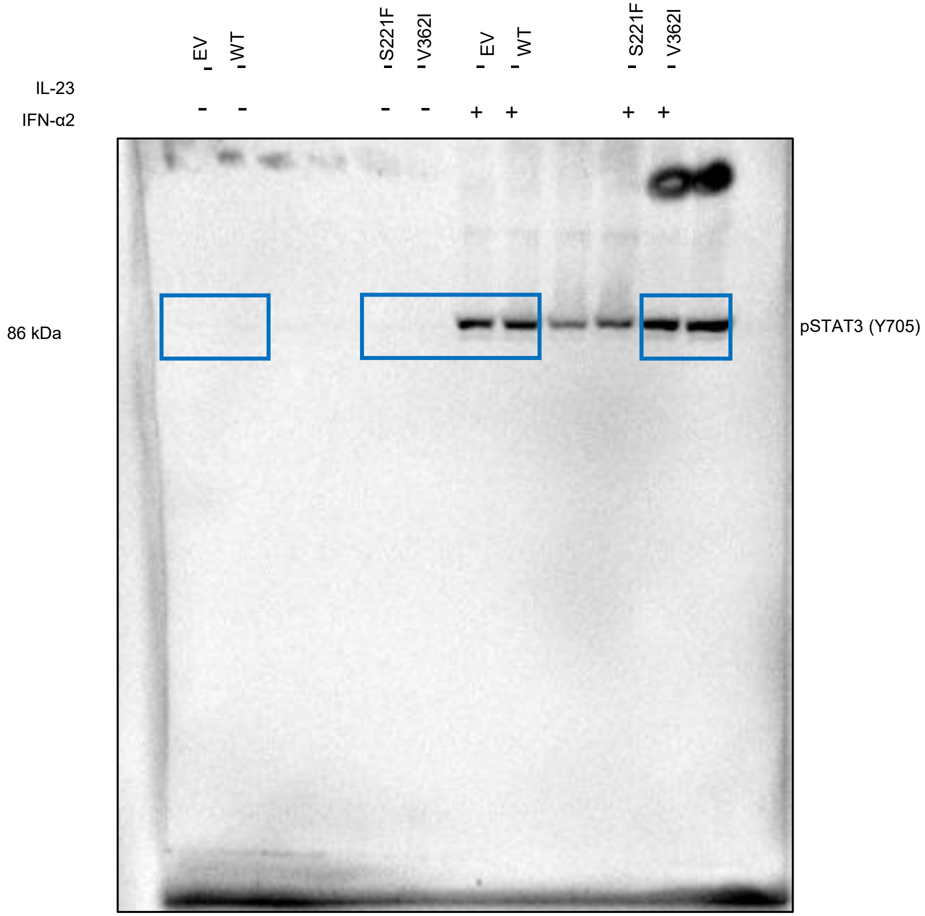

Showed

SourceDataFS1

B

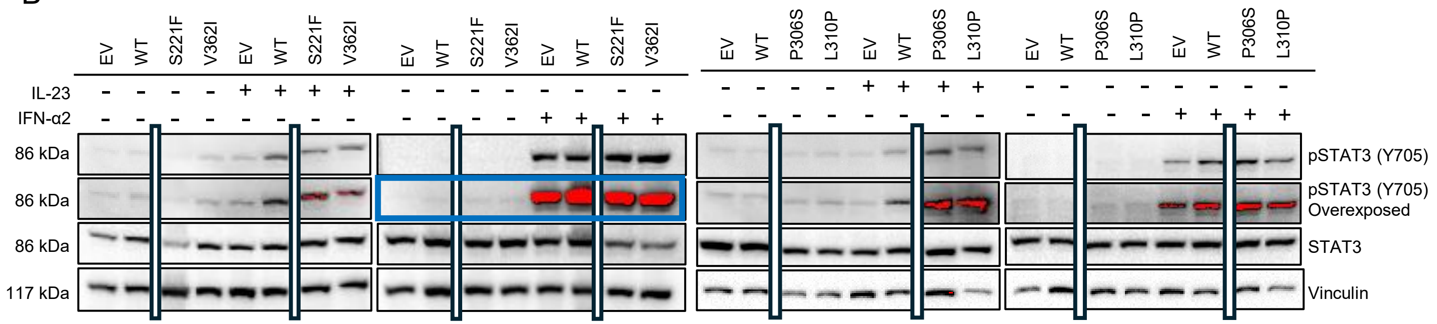

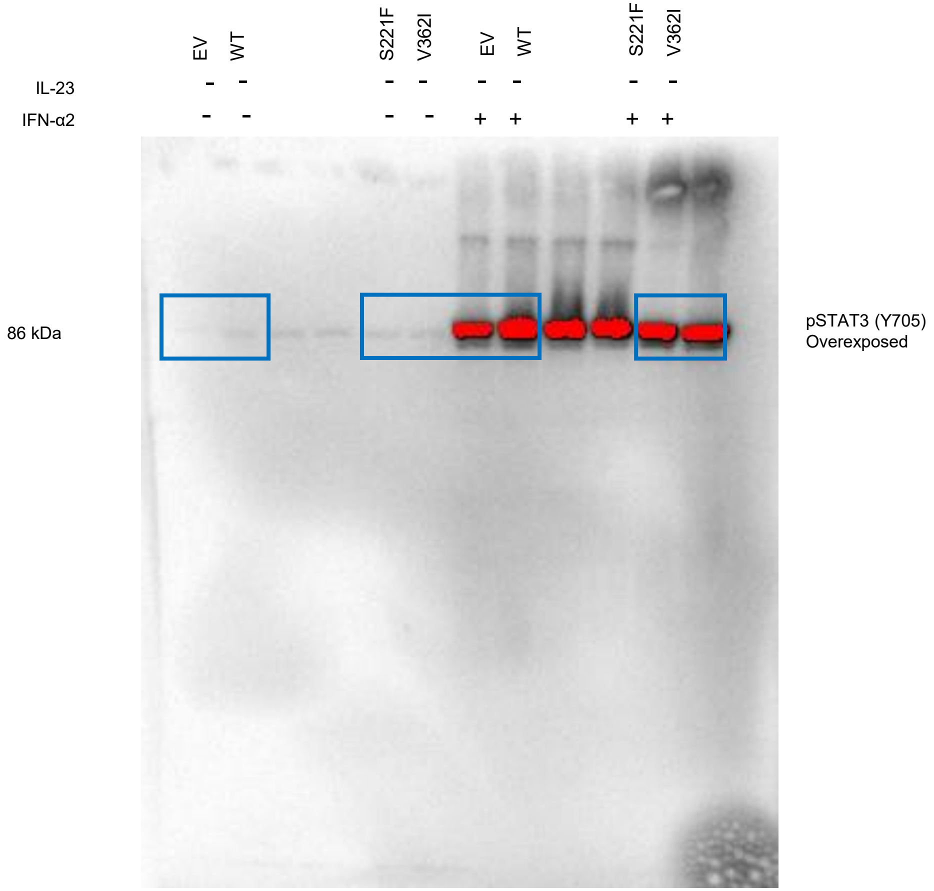

Showed

SourceDataFS1

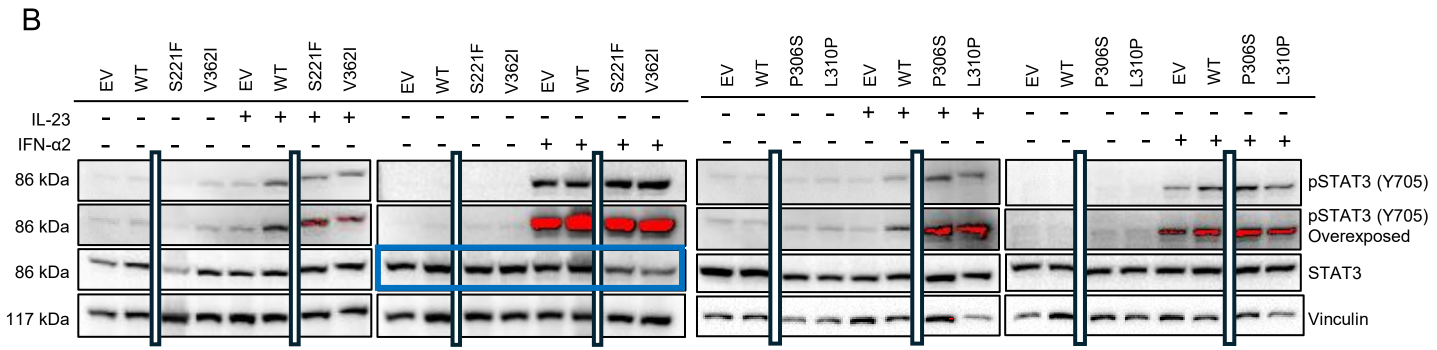

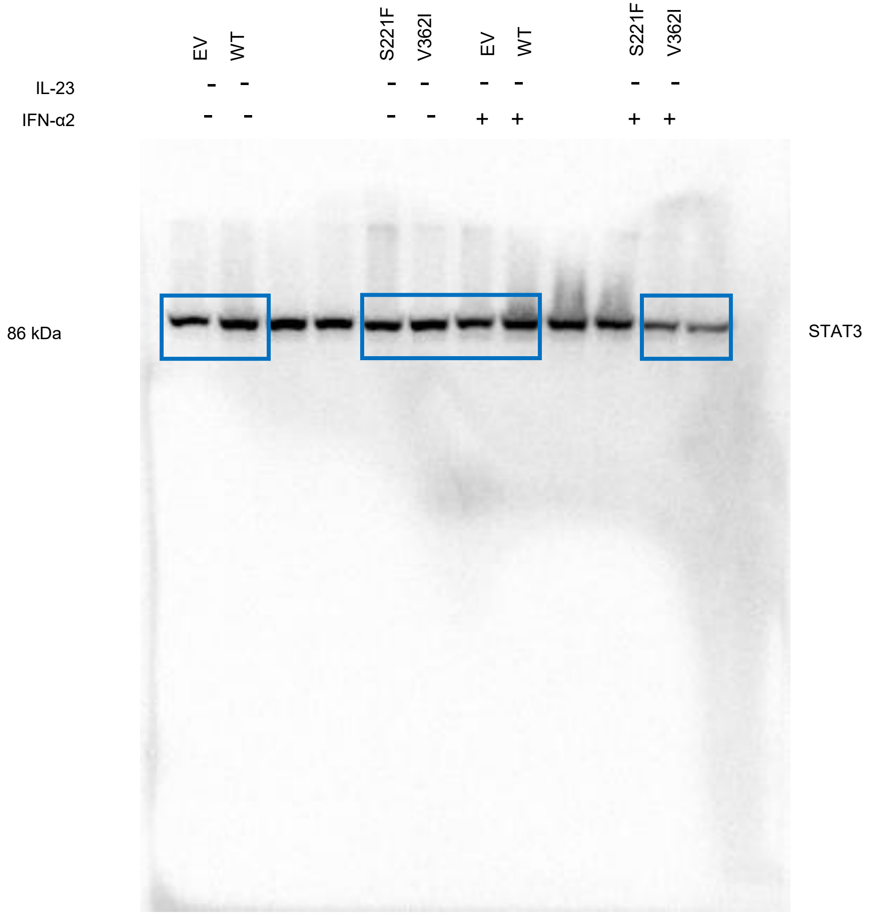

Showed

SourceDataFS1

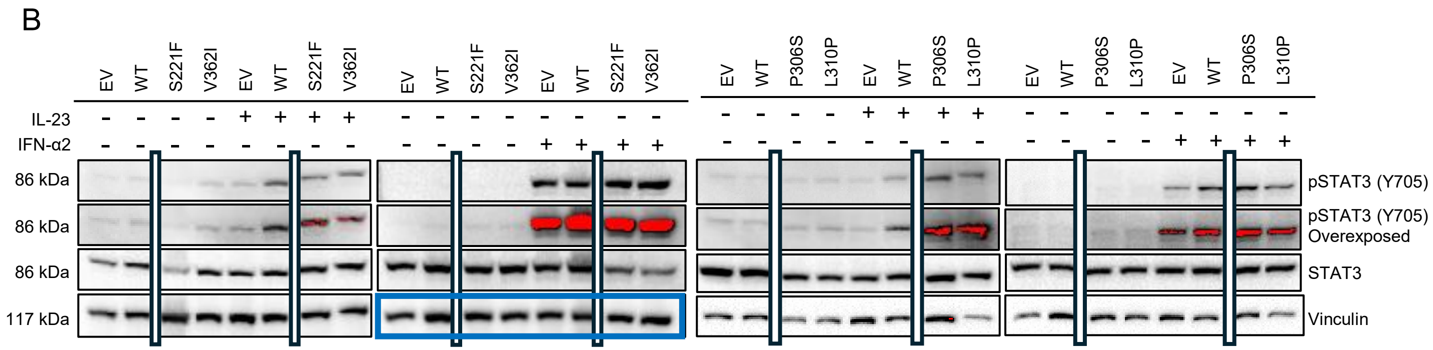

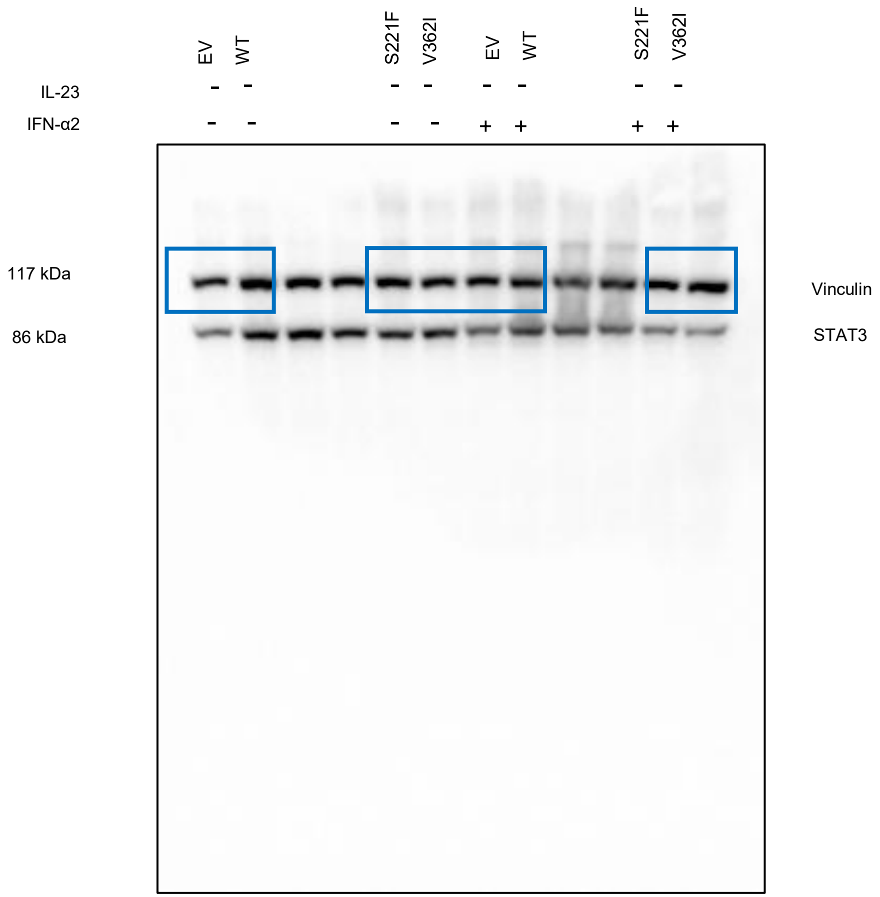

Showed

SourceDataFS1

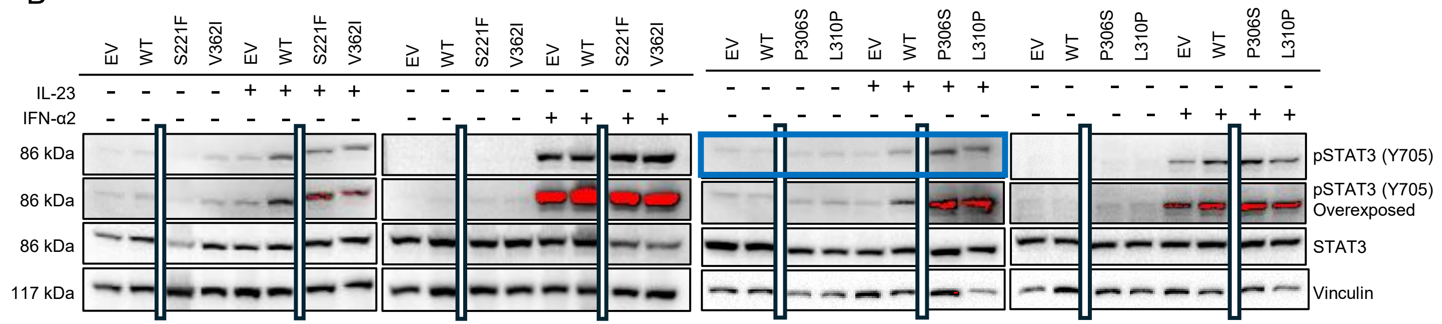

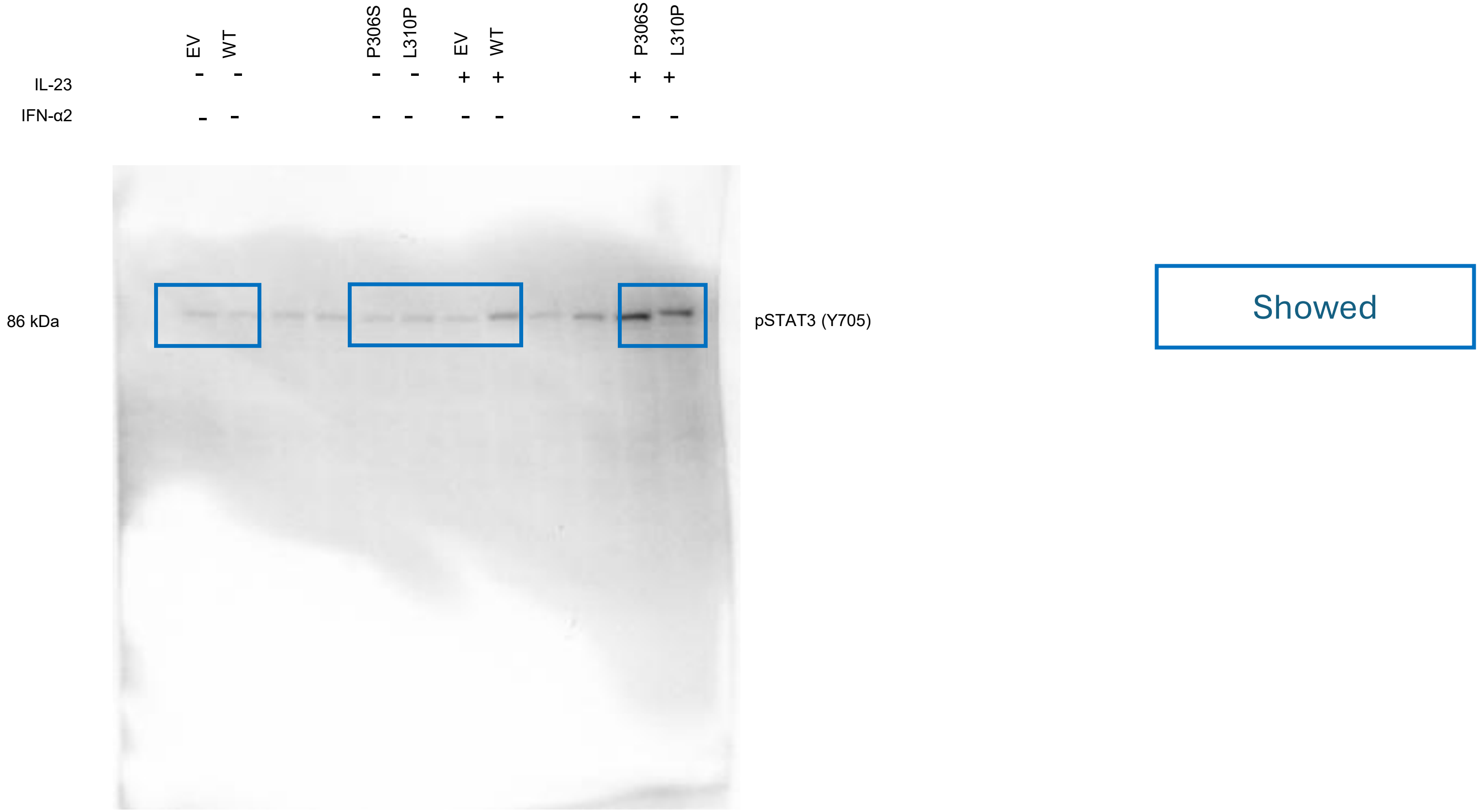

SourceDataFS1

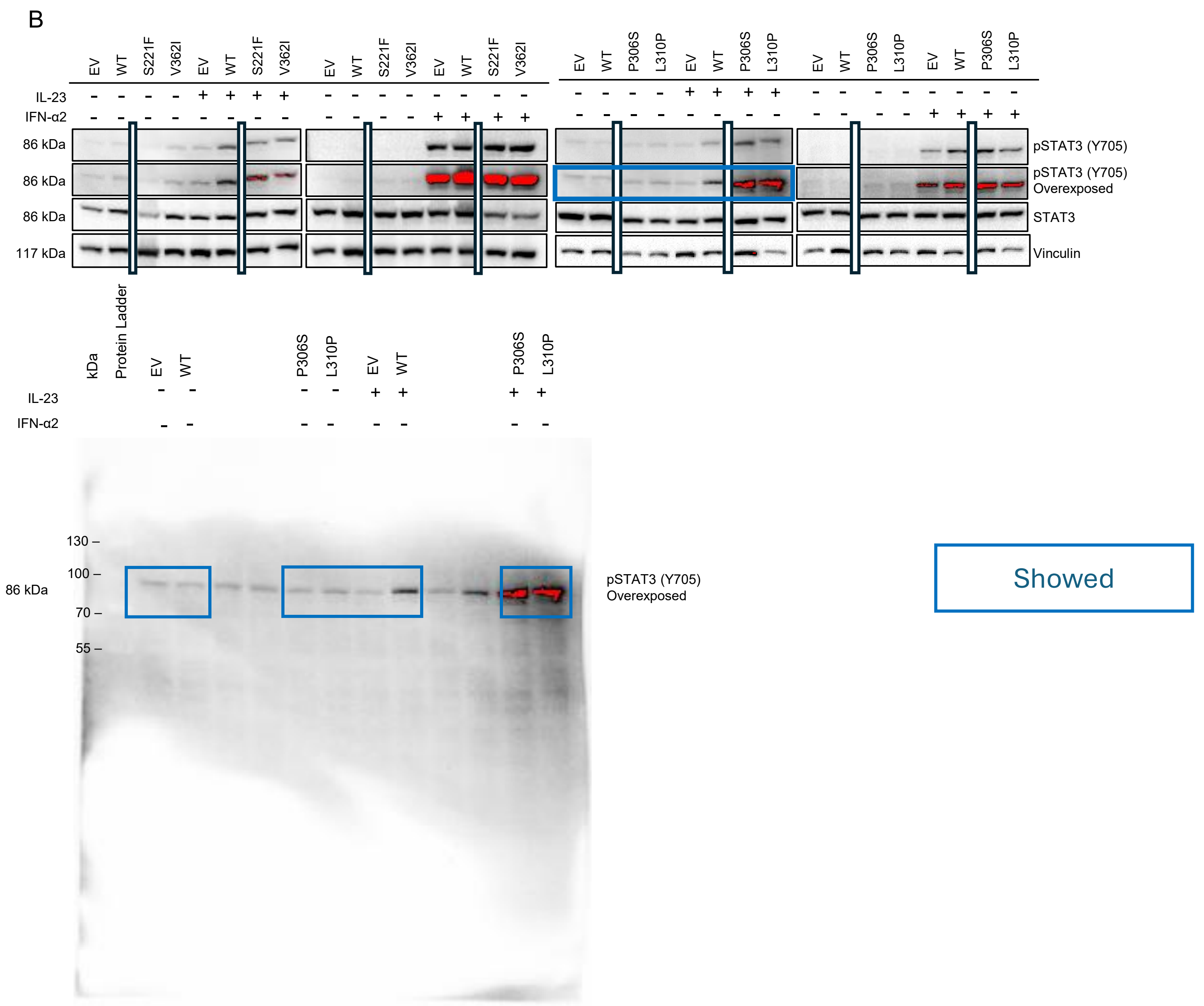

SourceDataFS1

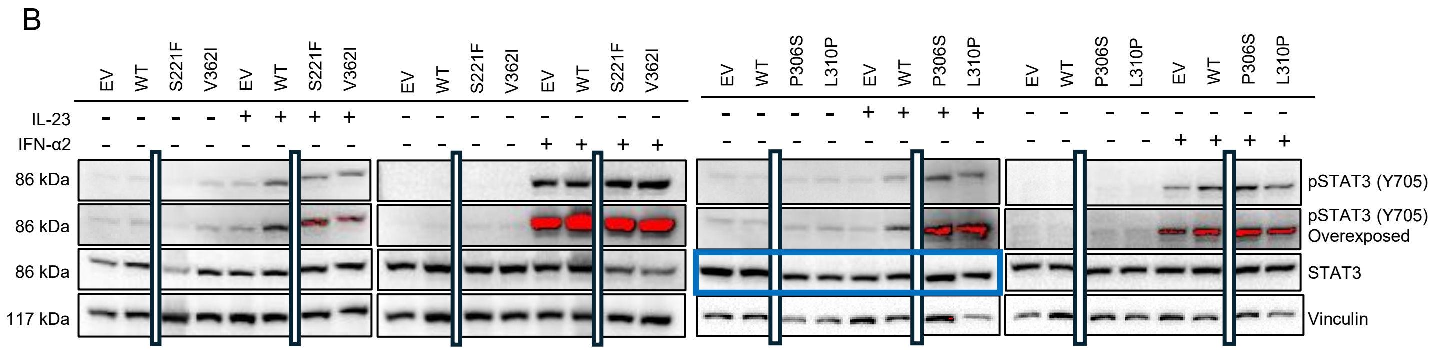

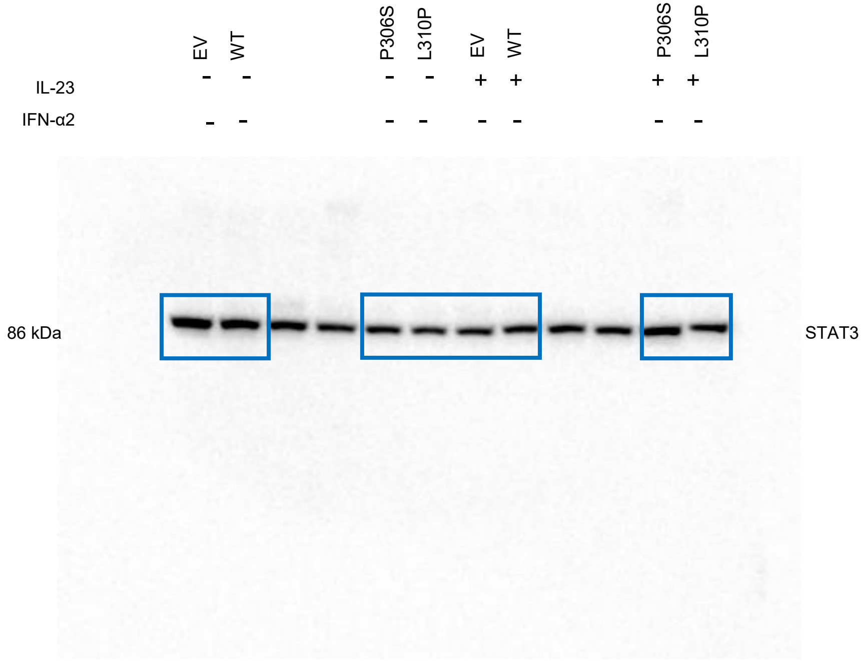

Showed

SourceDataFS1

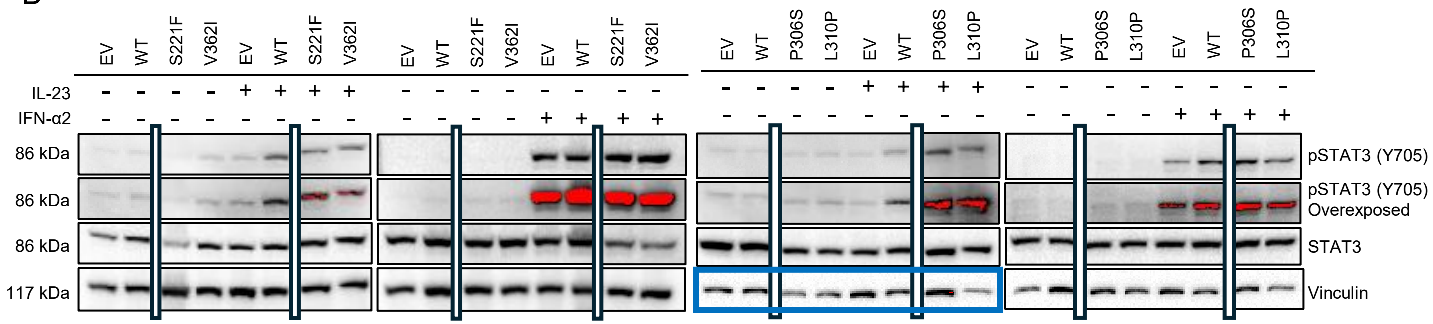

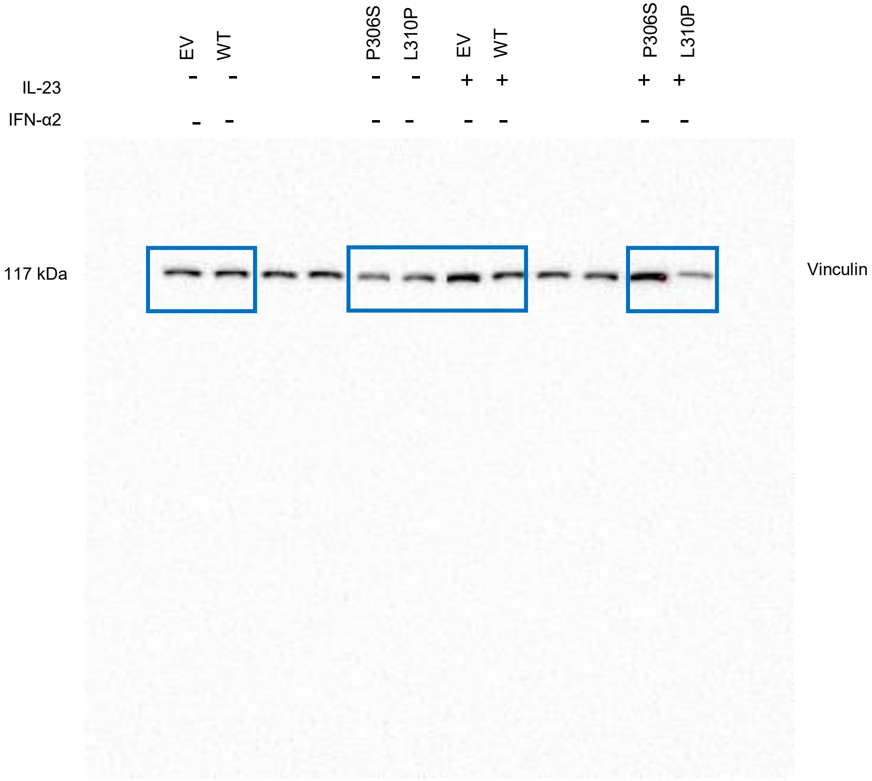

Showed

SourceDataFS1

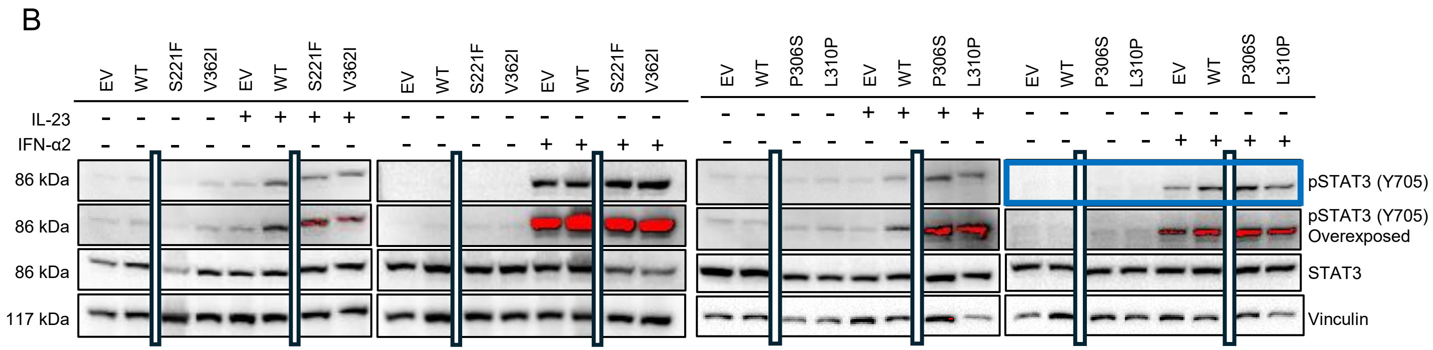

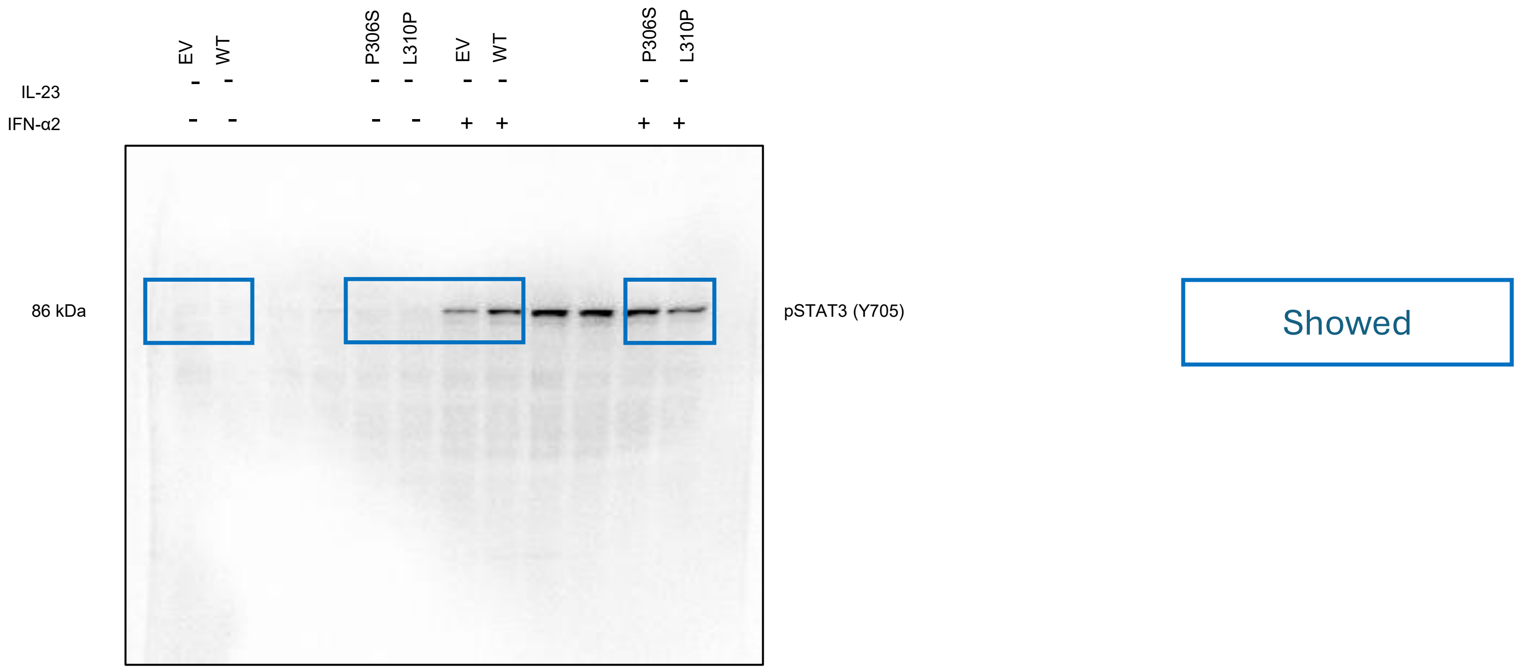

SourceDataFS1

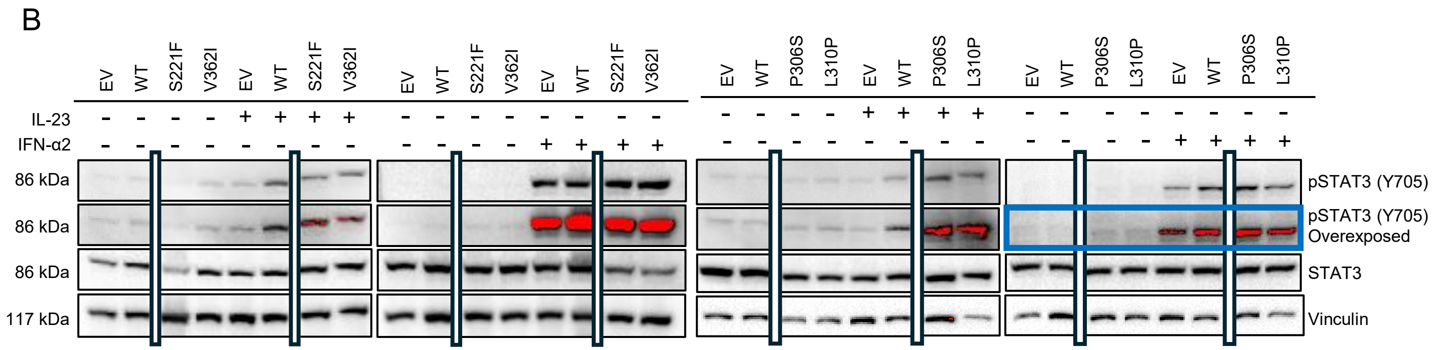

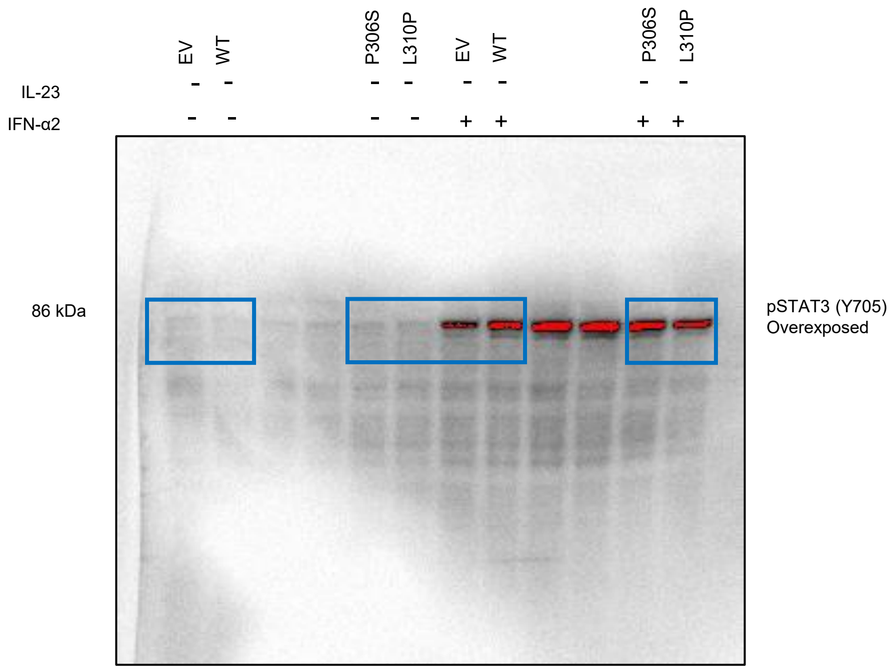

Showed

SourceDataFS1

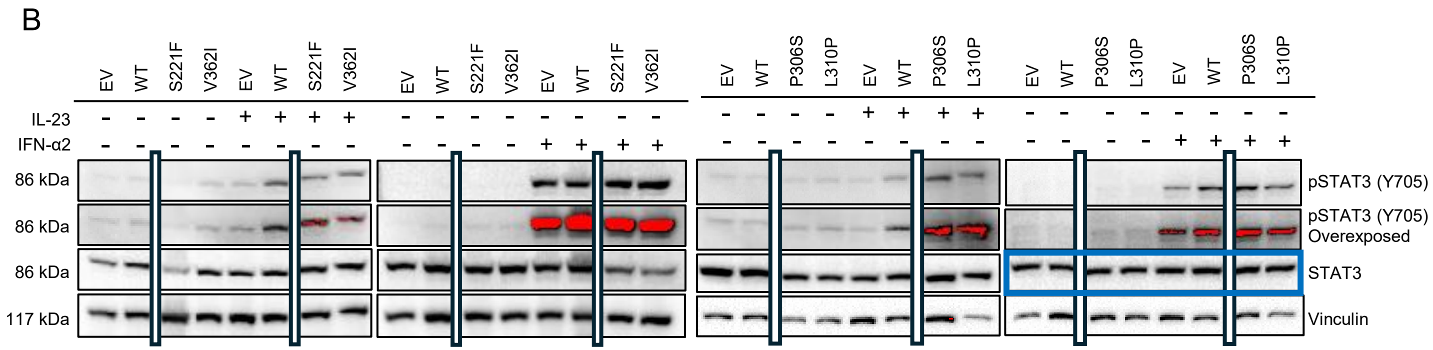

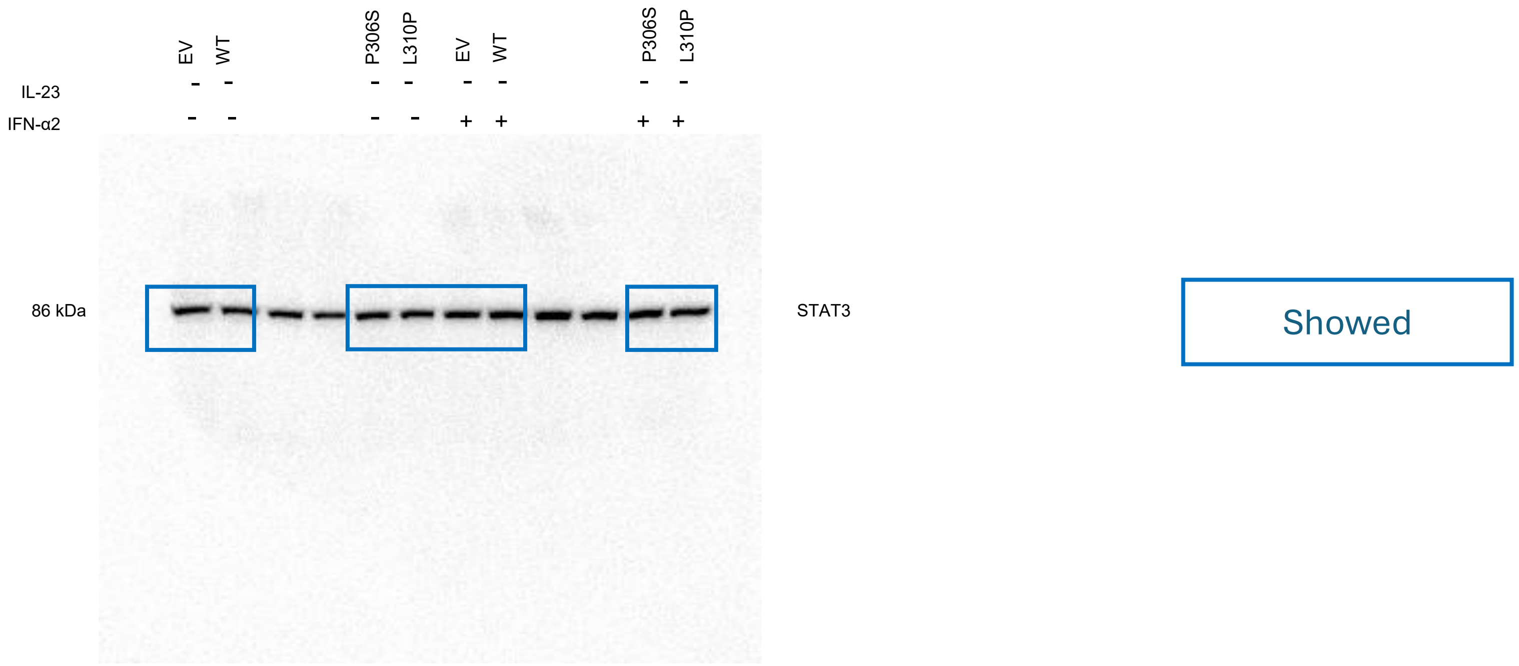

SourceDataFS1

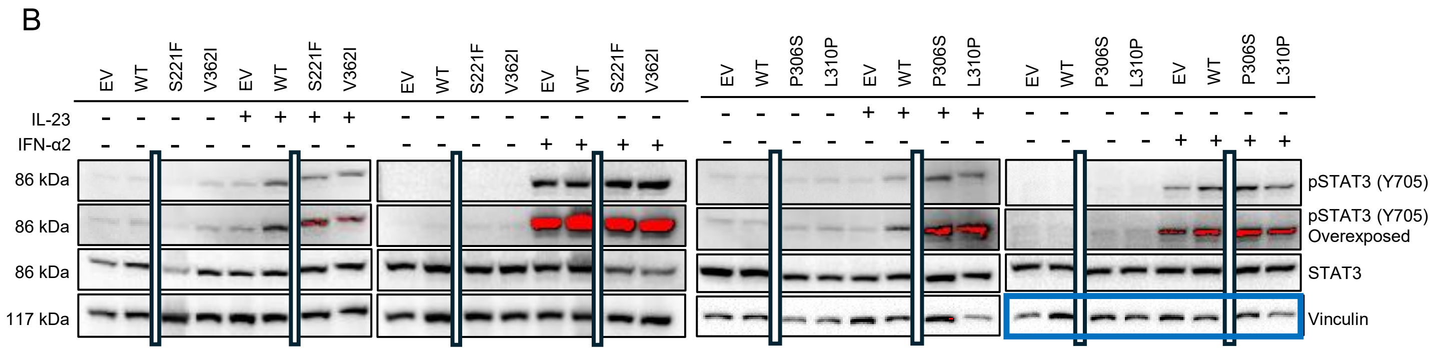

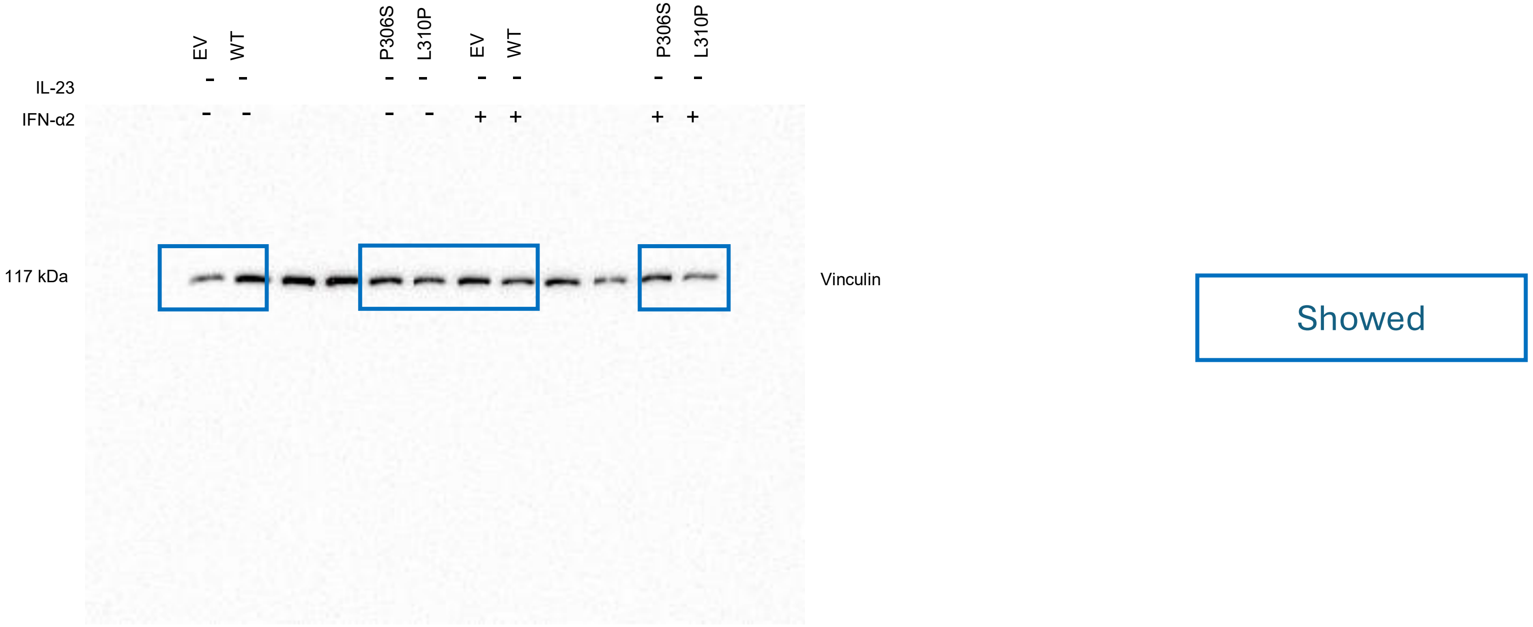

SourceDataFS1

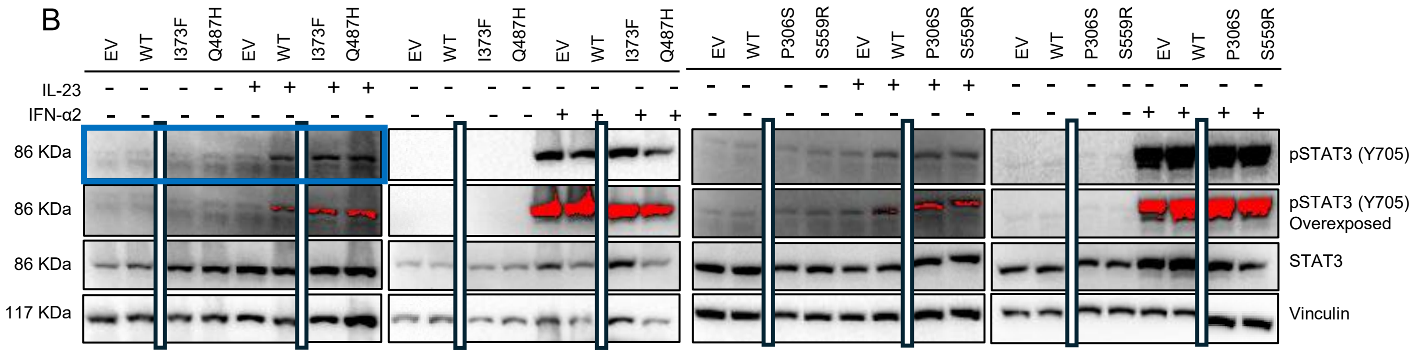

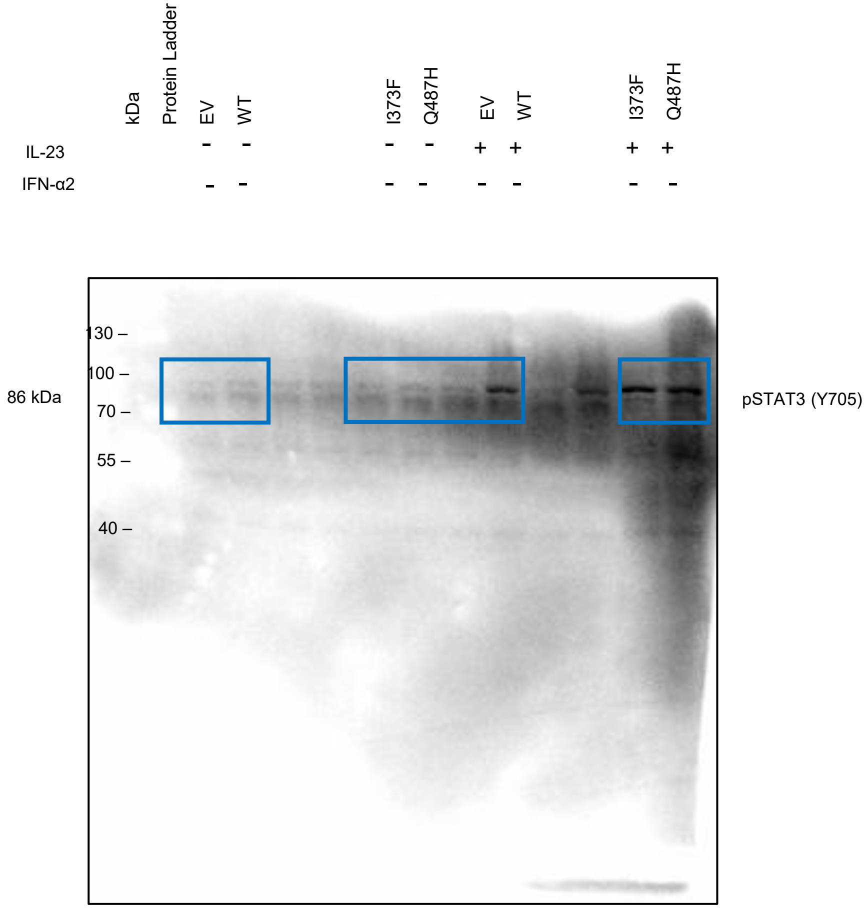

Showed

SourceDataFS1

B

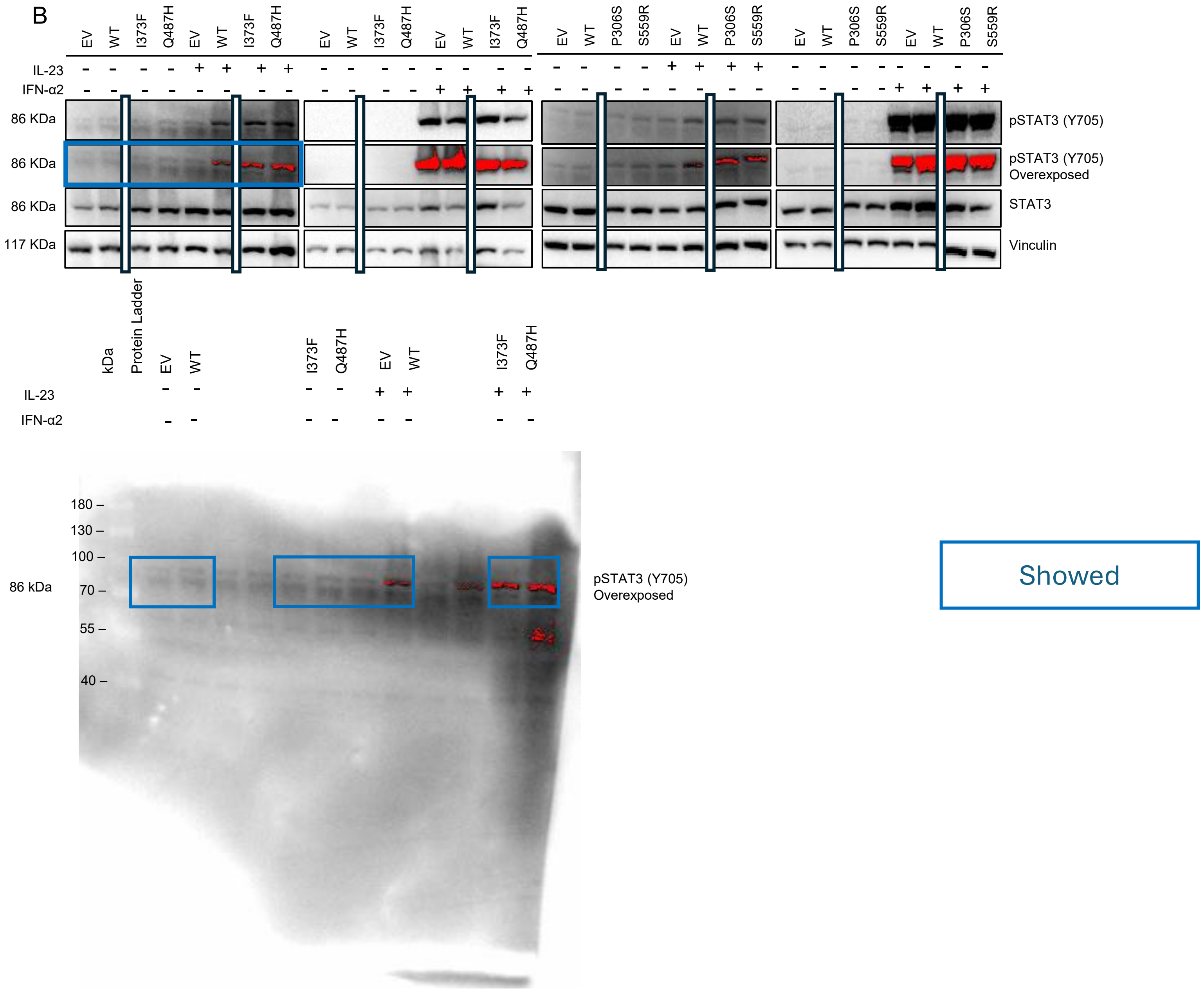

SourceDataFS1

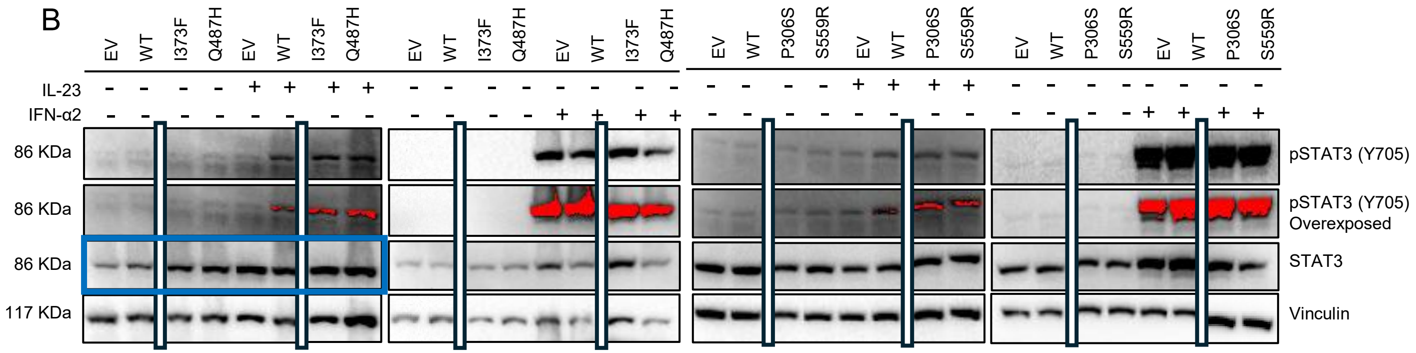

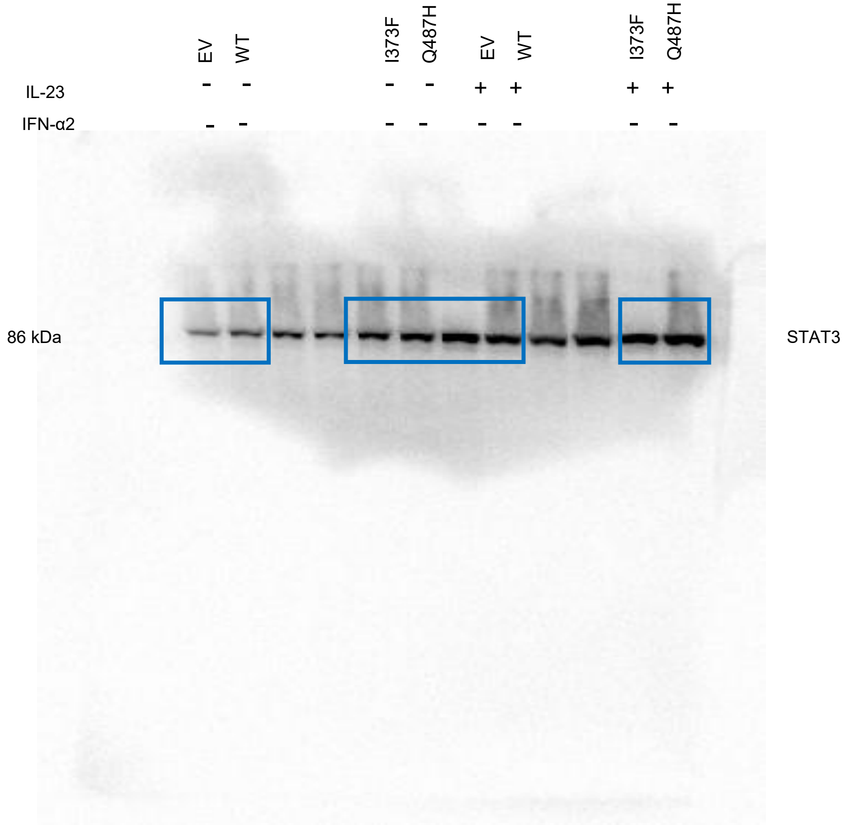

Showed

SourceDataFS1

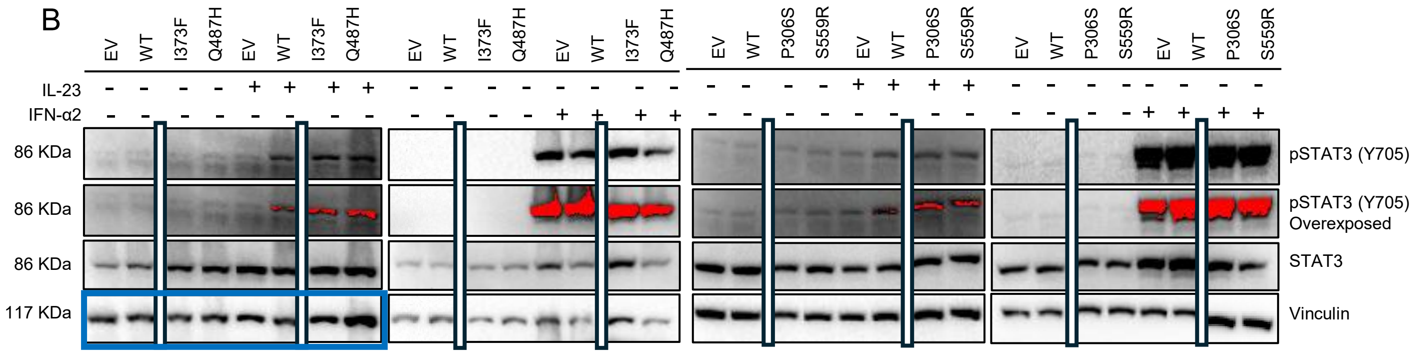

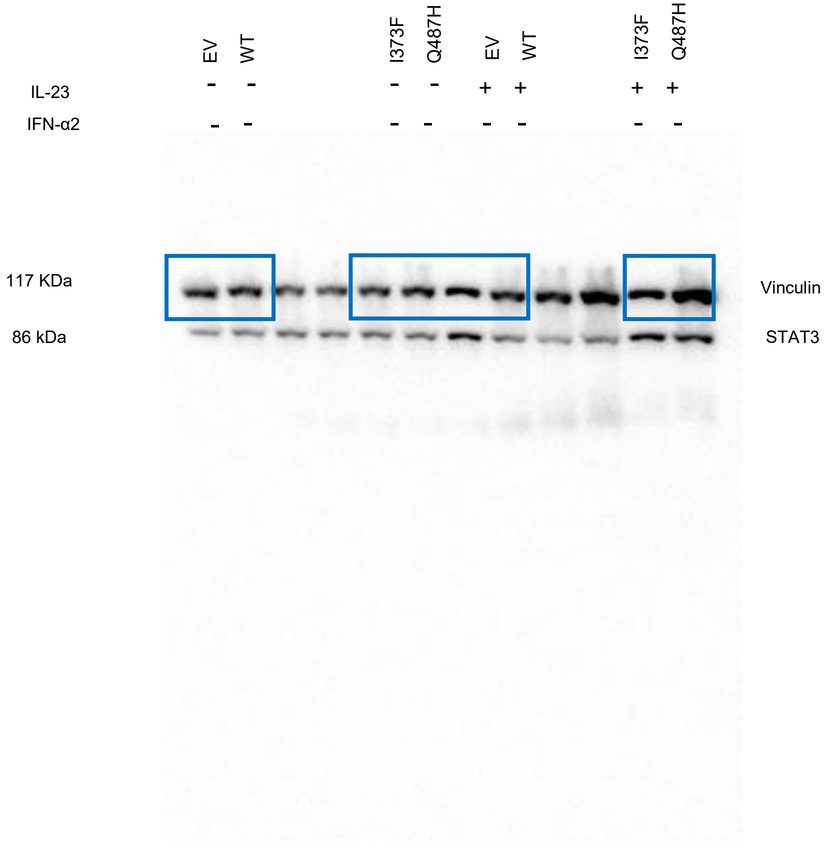

Showed

SourceDataFS1

B

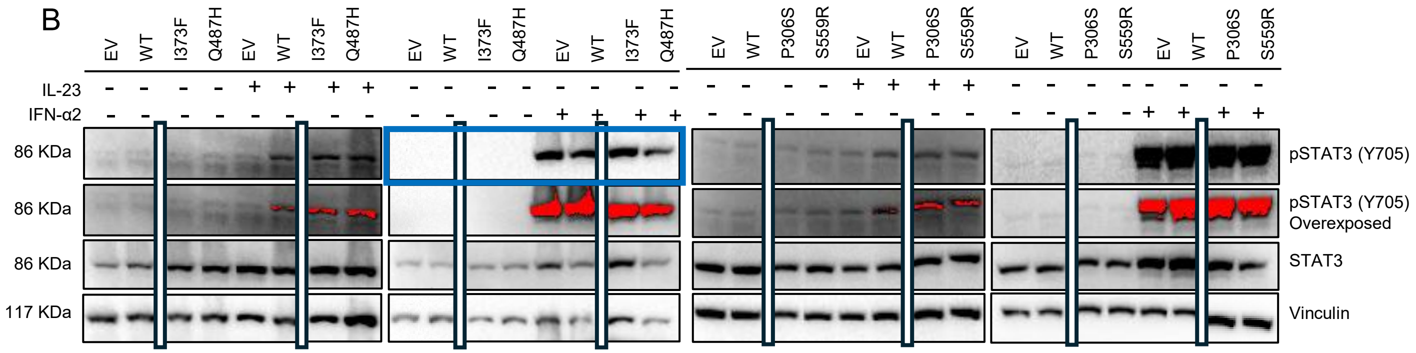

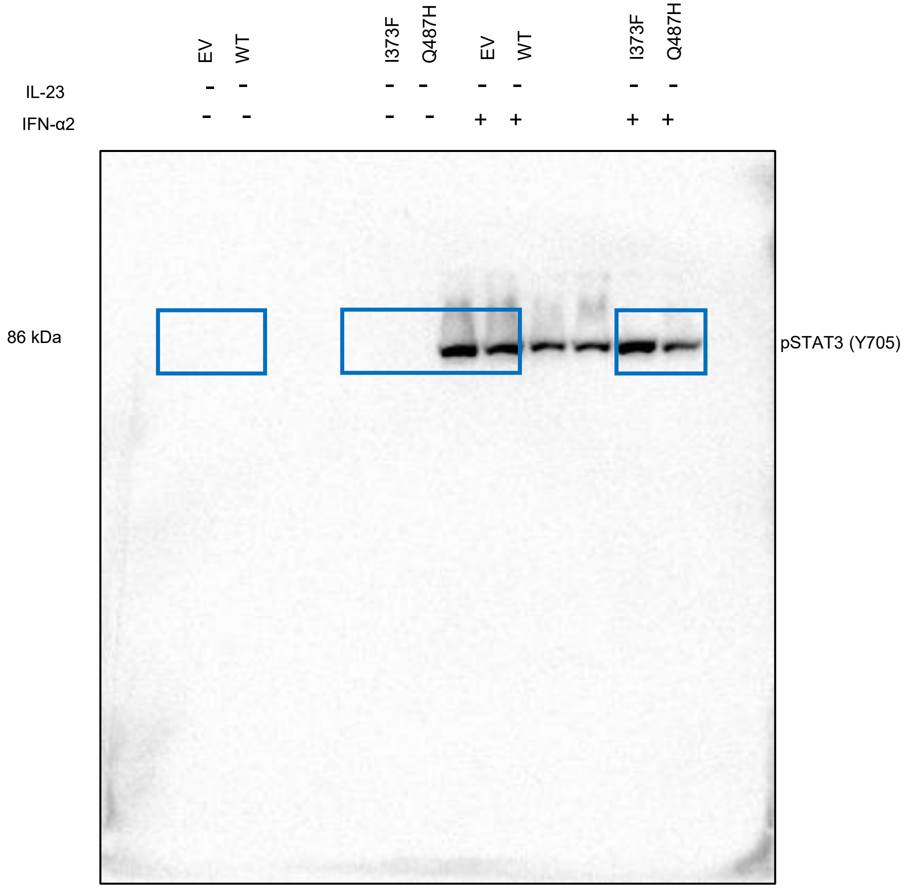

Showed

SourceDataFS1

B

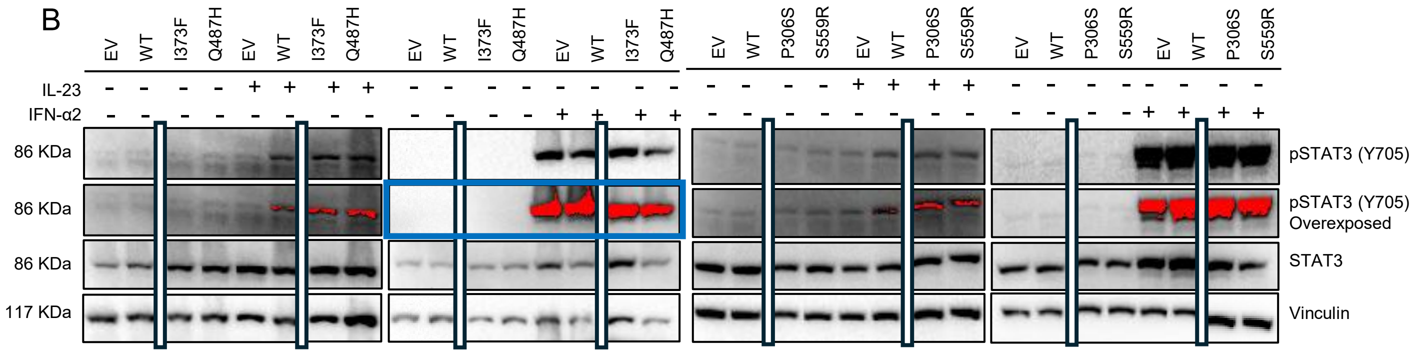

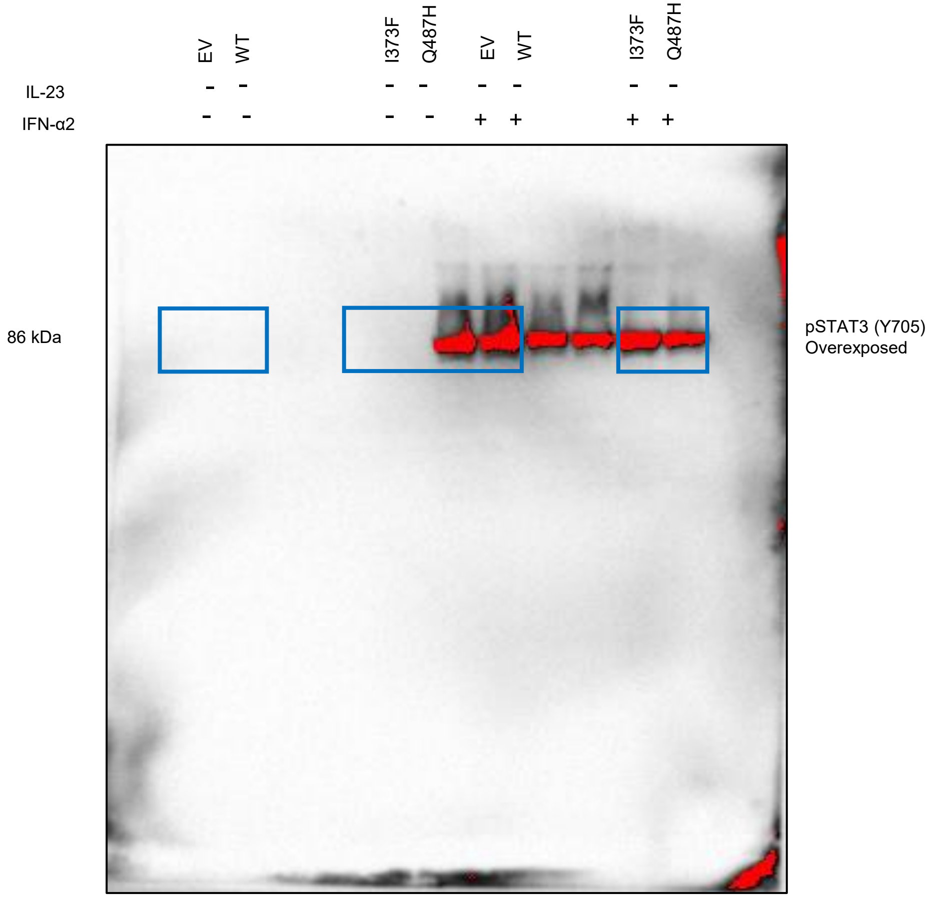

Showed

SourceDataFS1

B

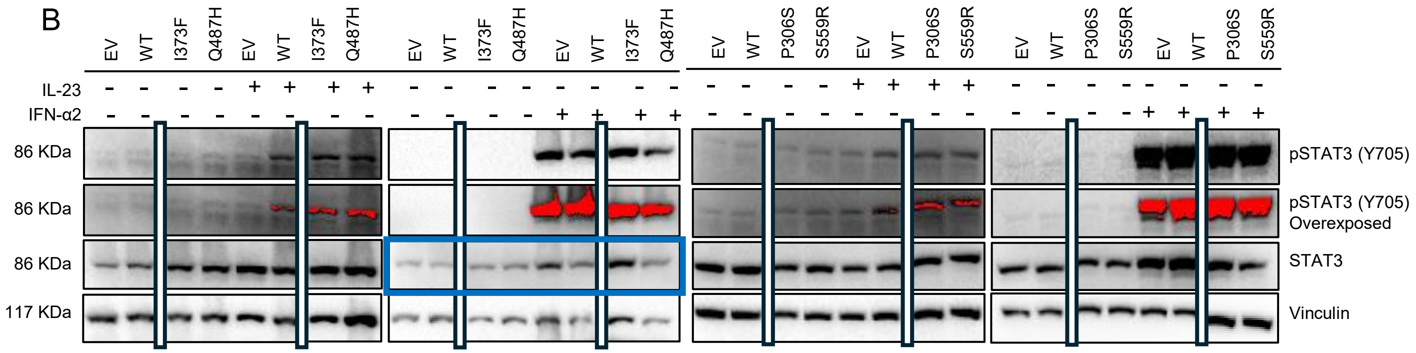

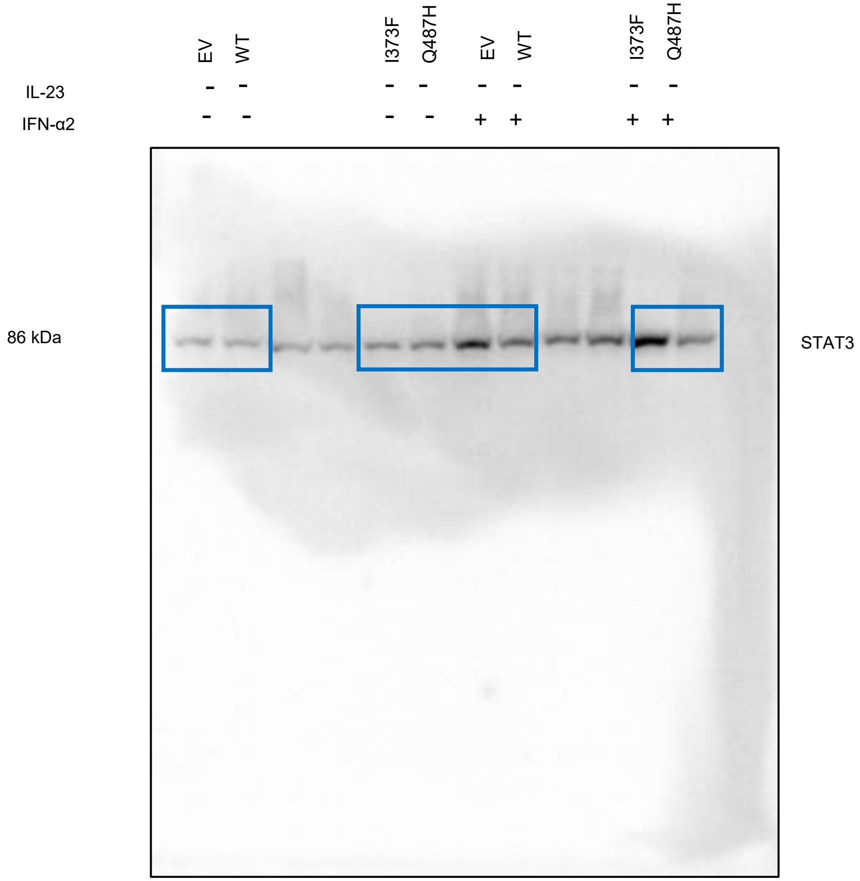

Showed

SourceDataFS1

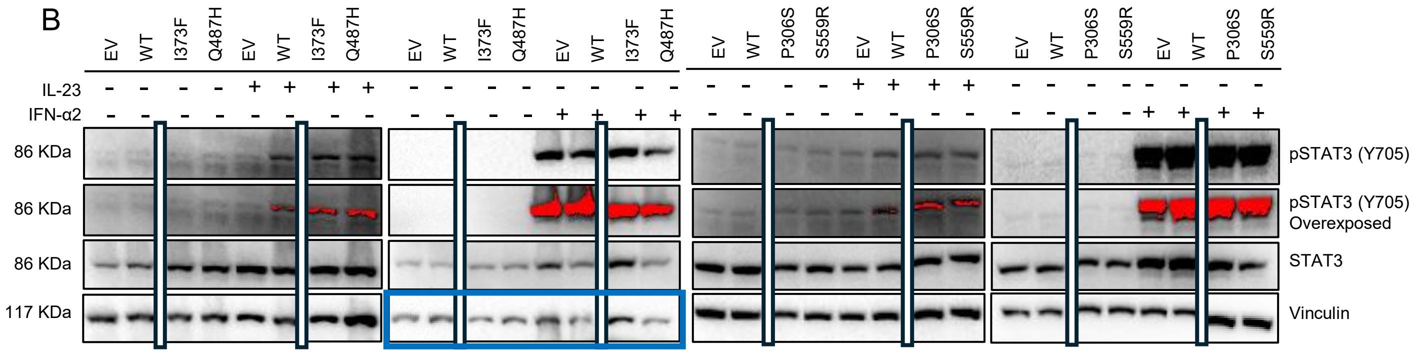

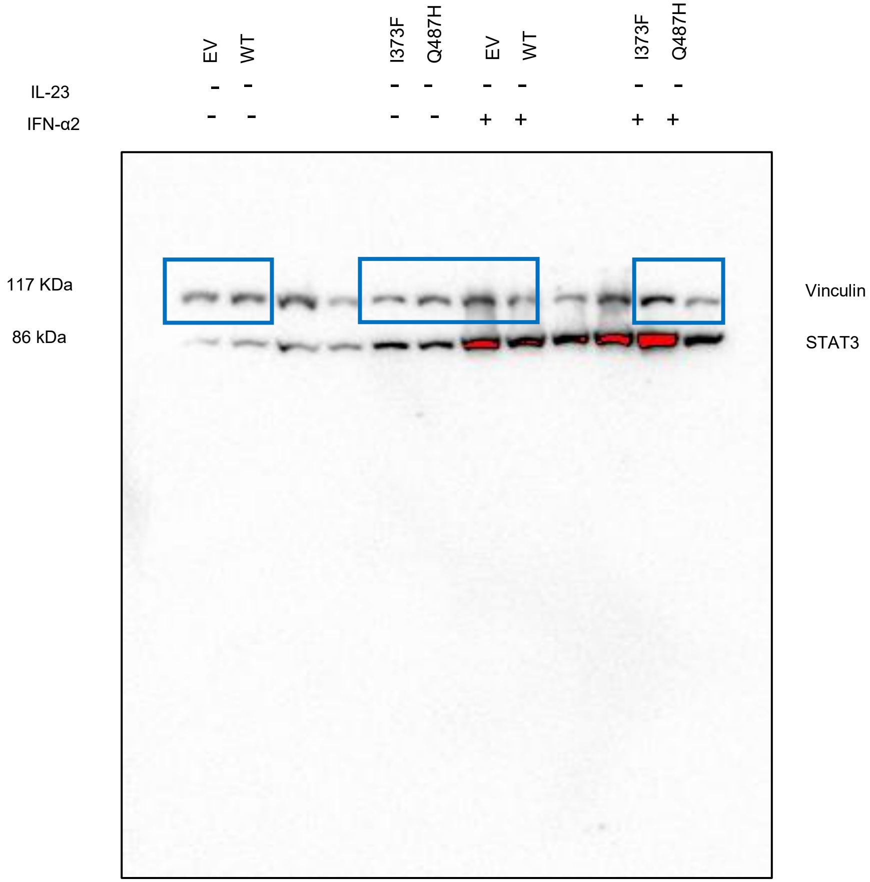

Showed

SourceDataFS1

B

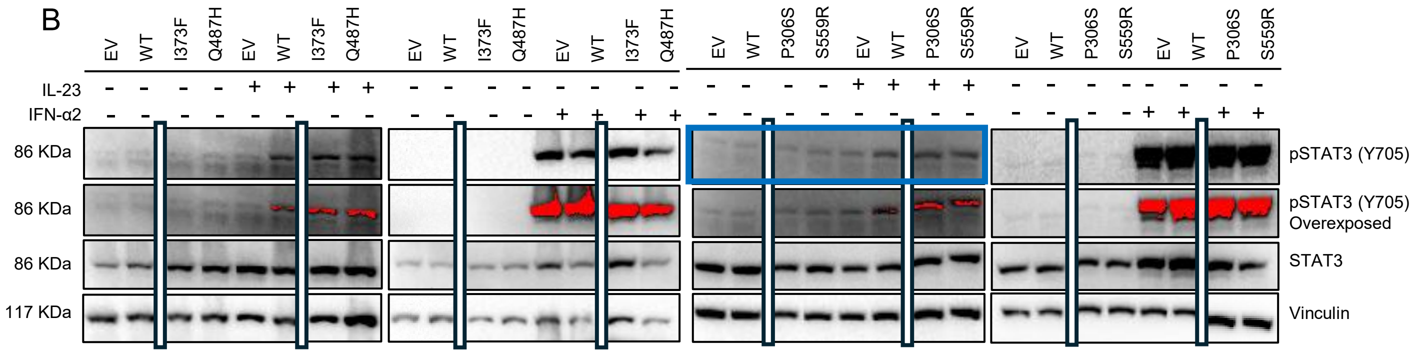

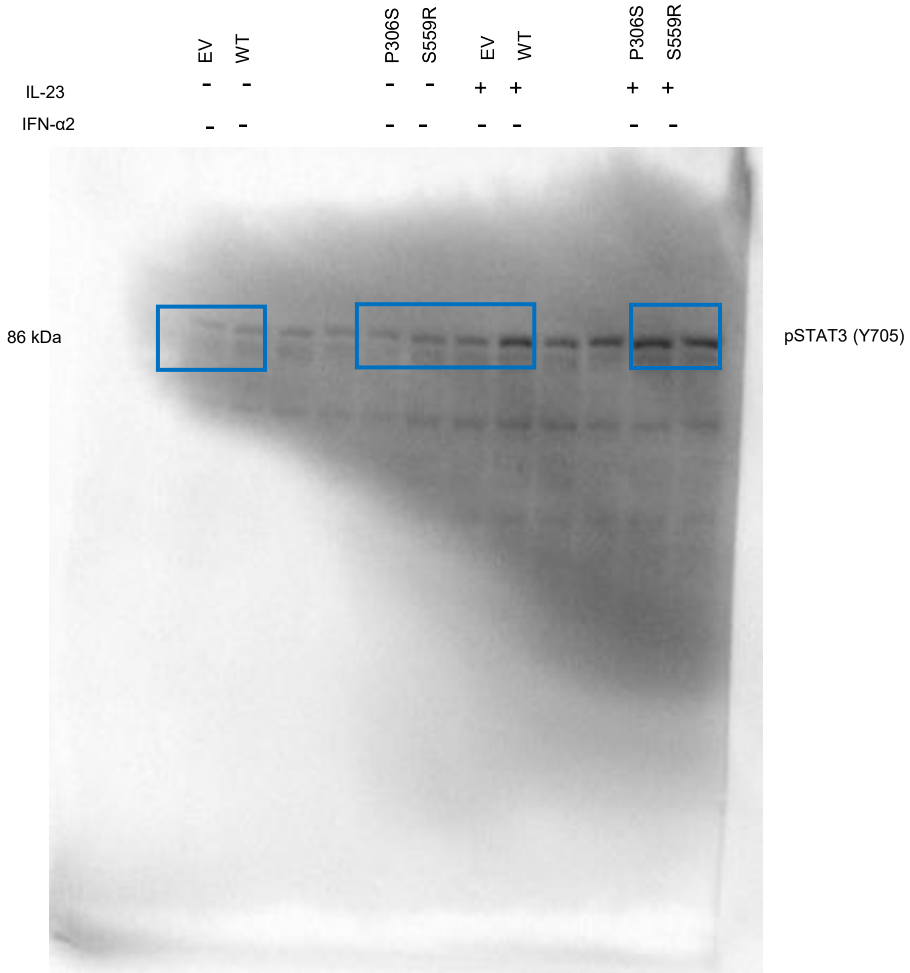

Showed

SourceDataFS1

B

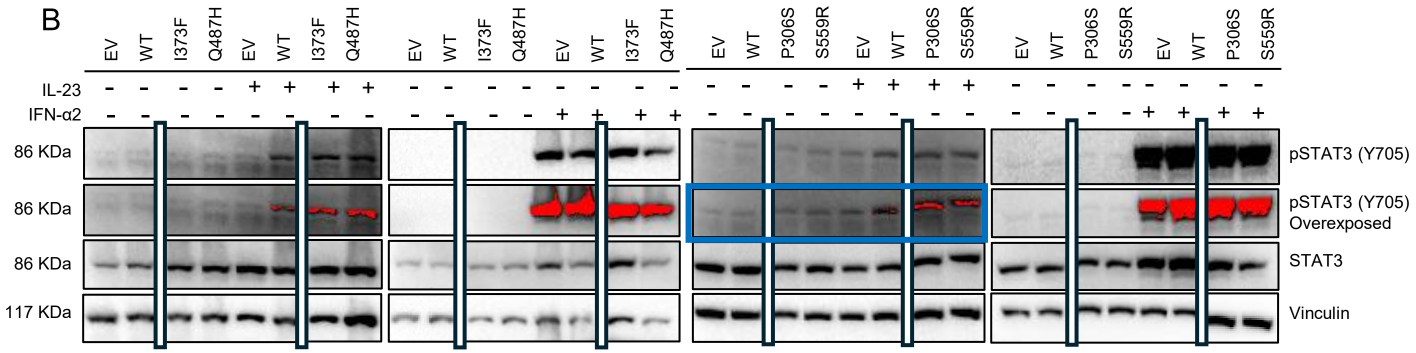

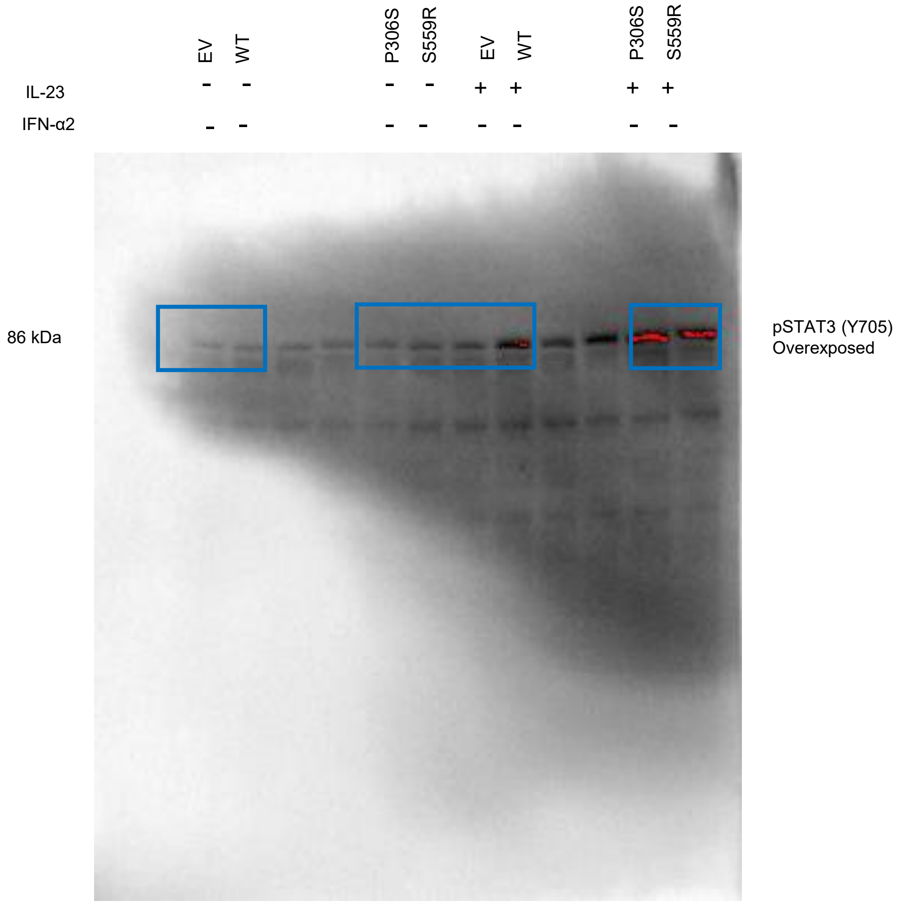

Showed

SourceDataFS1

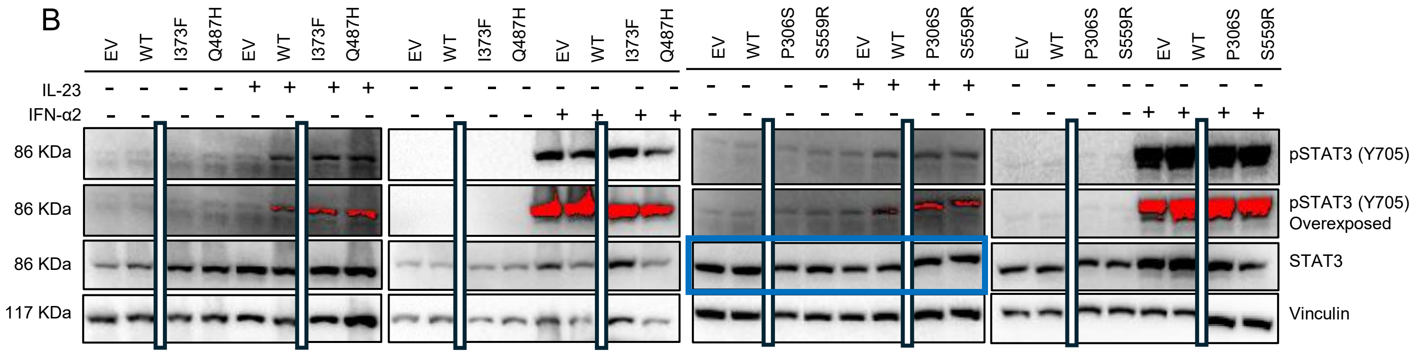

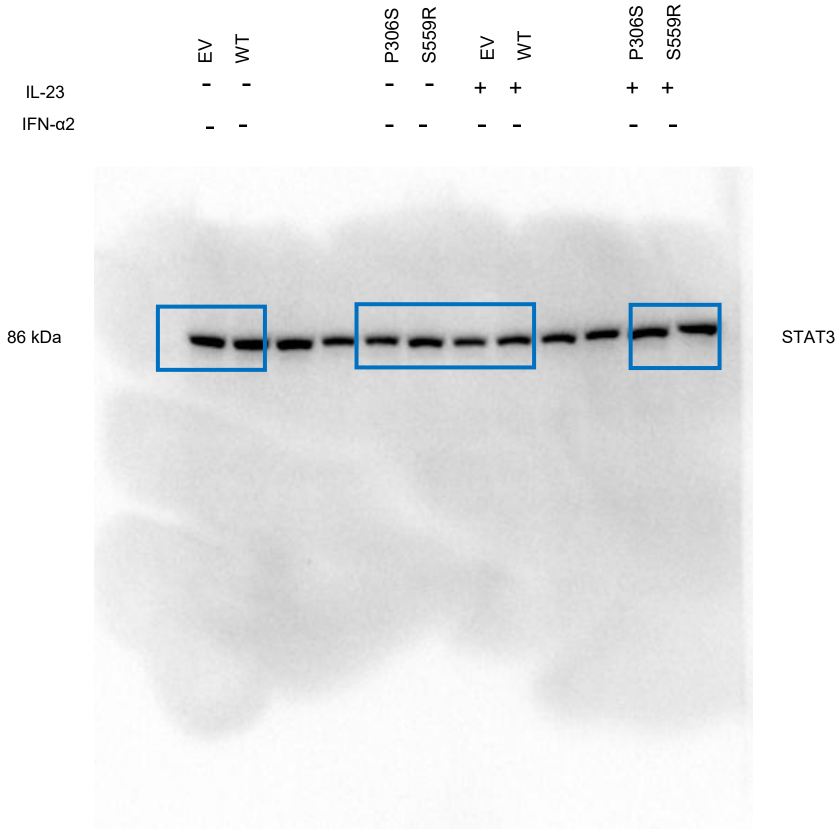

Showed

SourceDataFS1

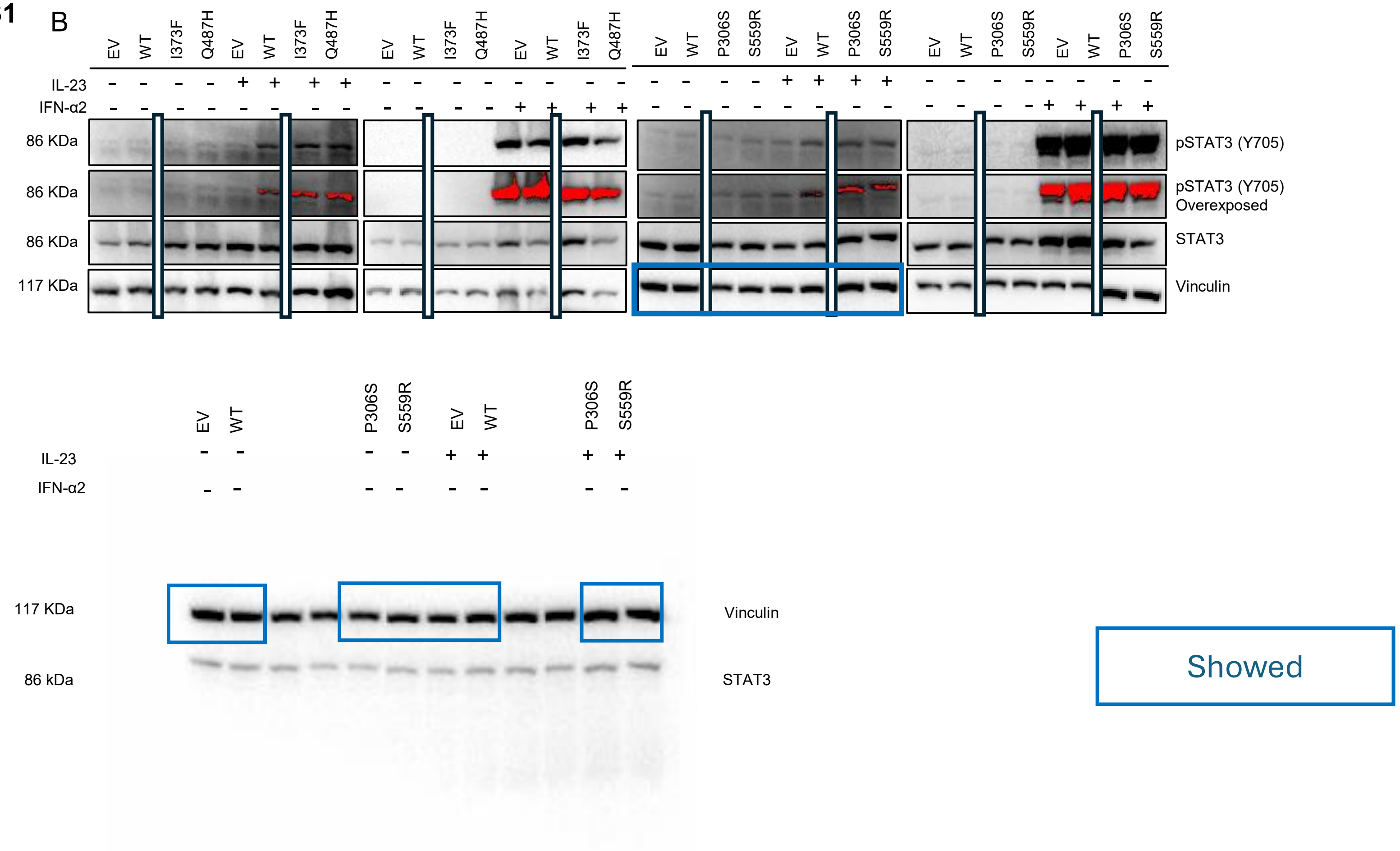

SourceDataFS1

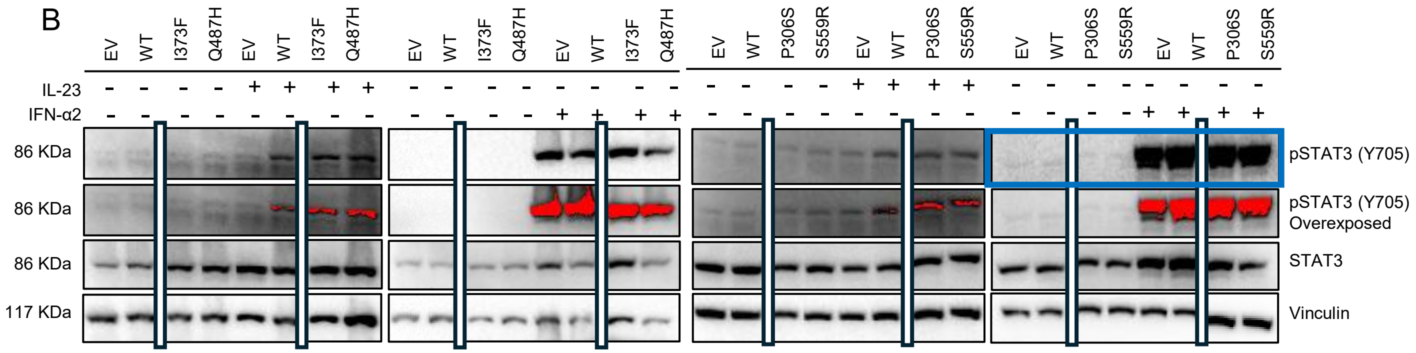

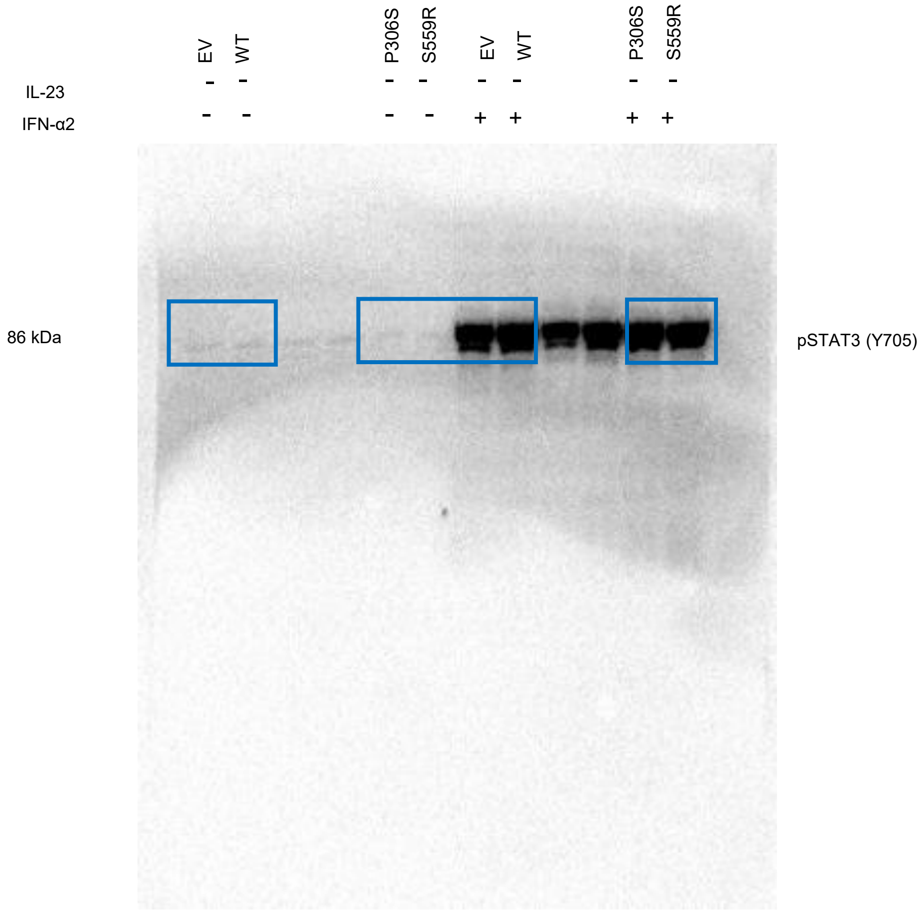

Showed

SourceDataFS1

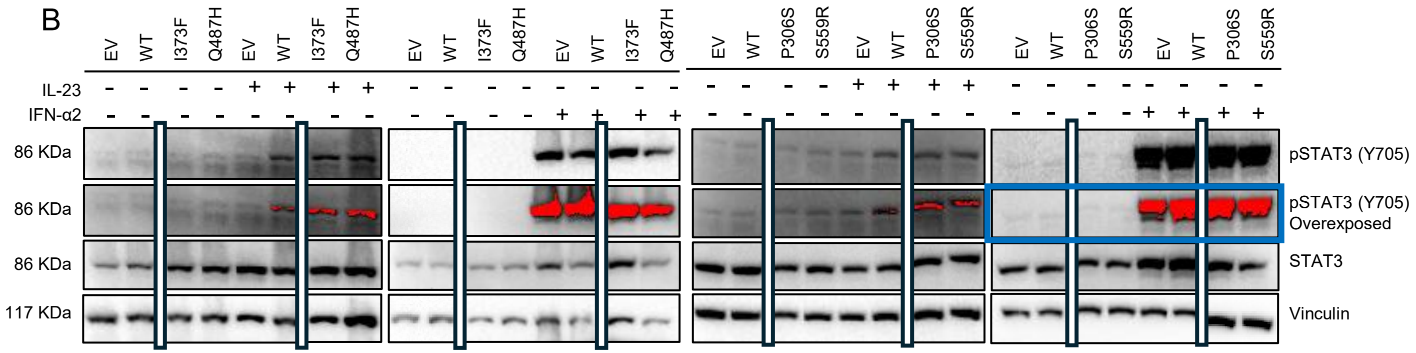

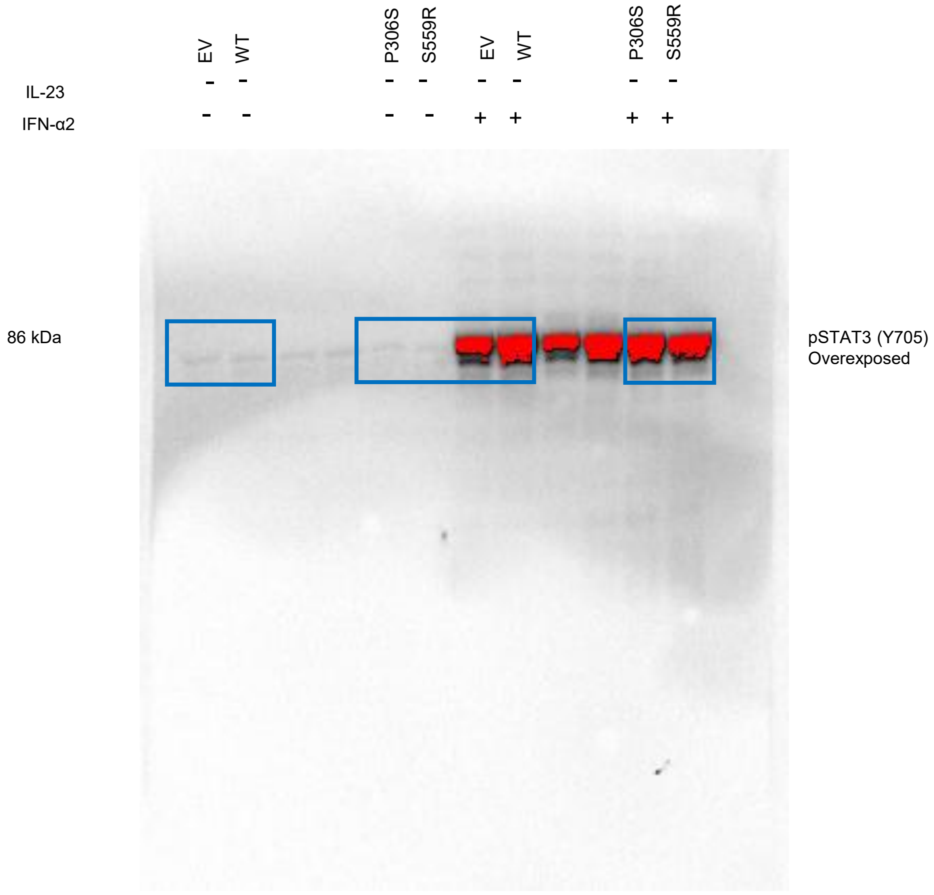

Showed

SourceDataFS1

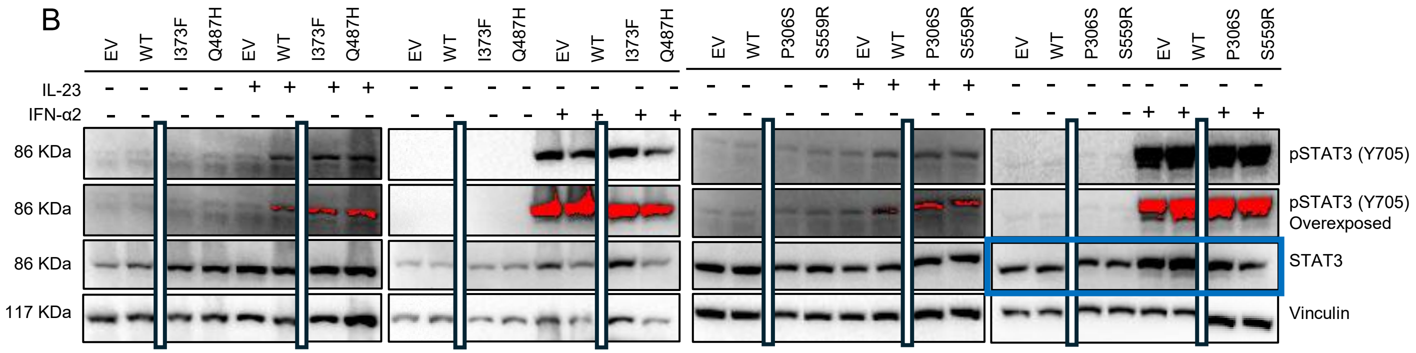

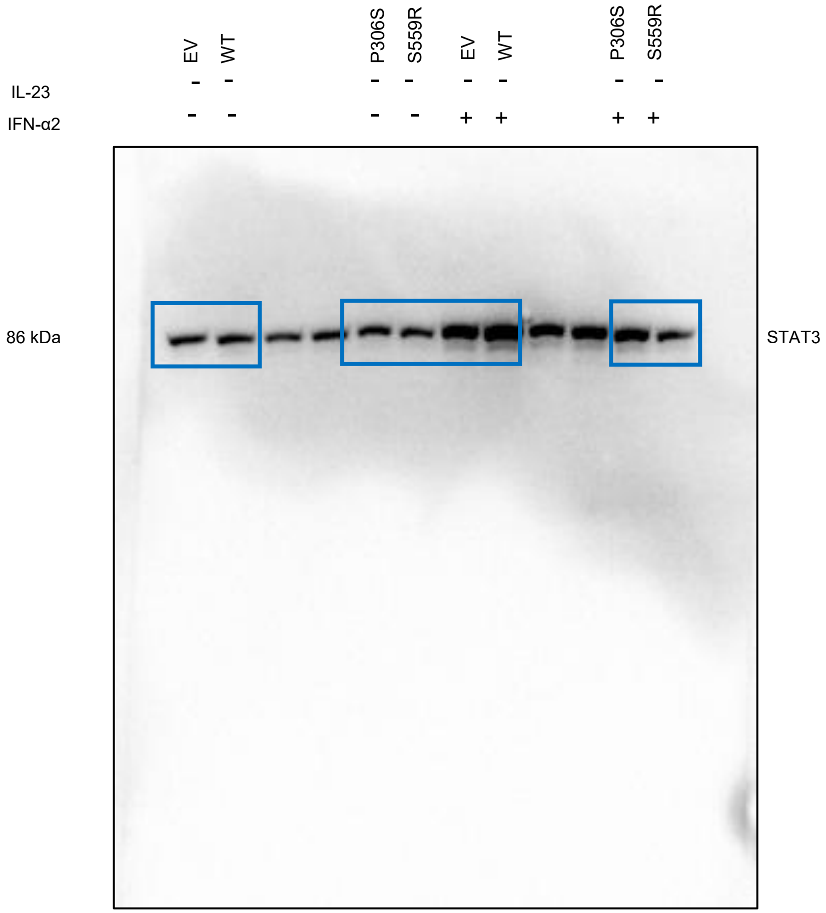

Showed

SourceDataFS1

B

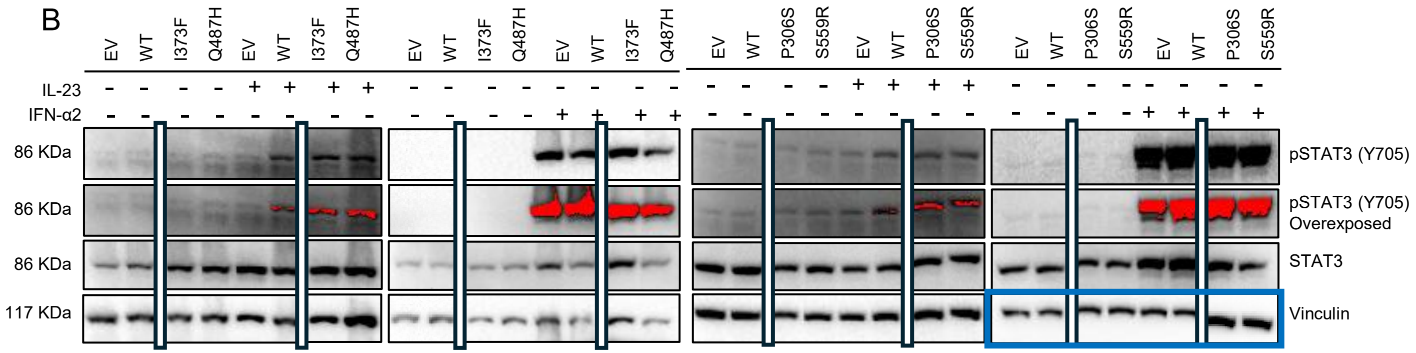

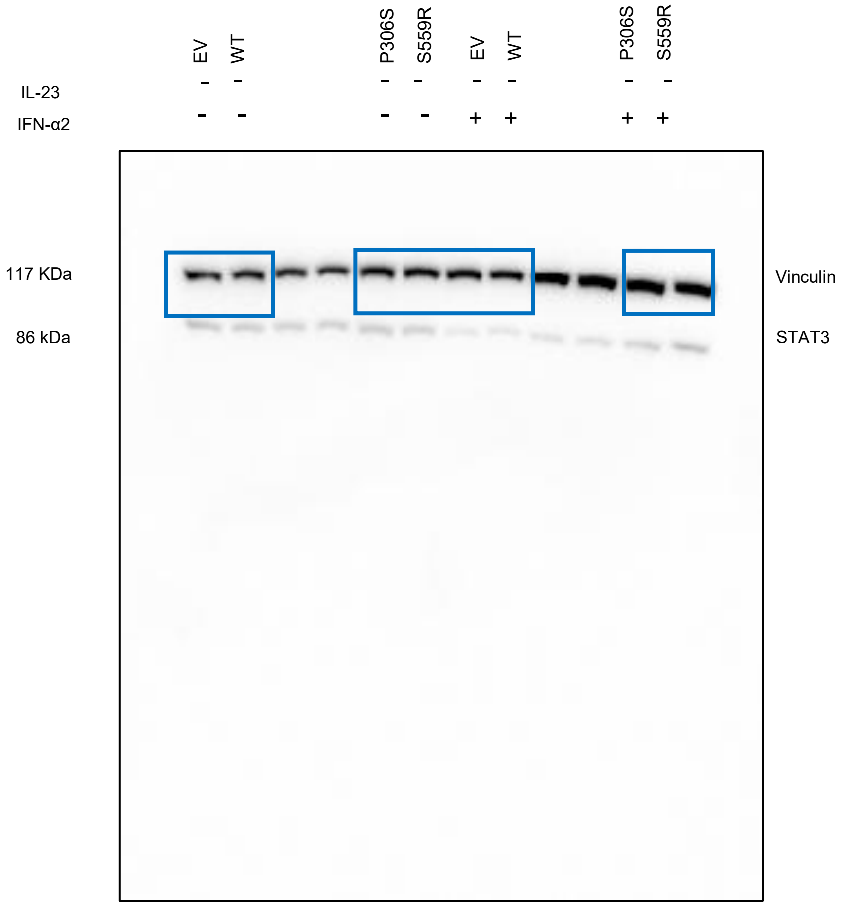

Showed
