# Supplementary material for: Humans homozygous for rare or common hypomorphic IL23R variants are prone to tuberculosis
Source: J Exp Med. 2026 Jul 9;223(8):e20252236. doi: 10.1084/jem.20252236 (PMC13348821; doi:10.1084/jem.20252236)
Supplement: SourceData FS2 — is the source file for Fig. S2. [file jem_20252236_sourcedatafs2.pdf]

**SourceDataFS2**

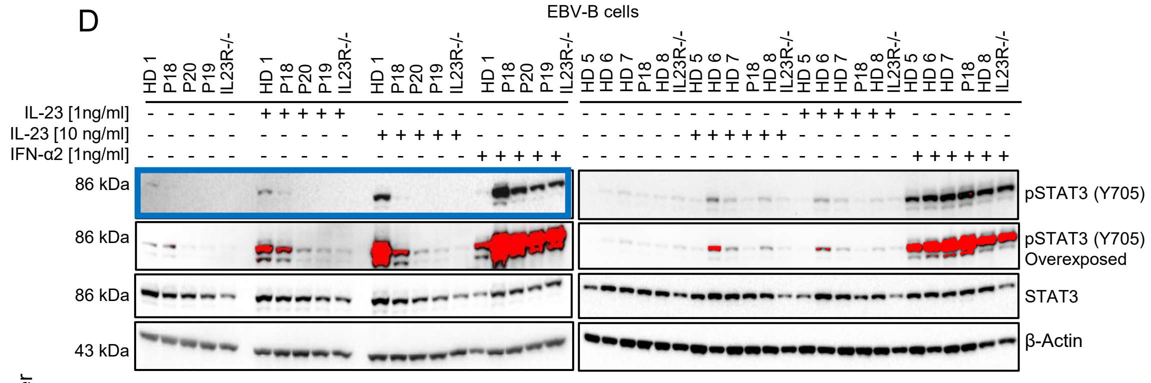


Showed

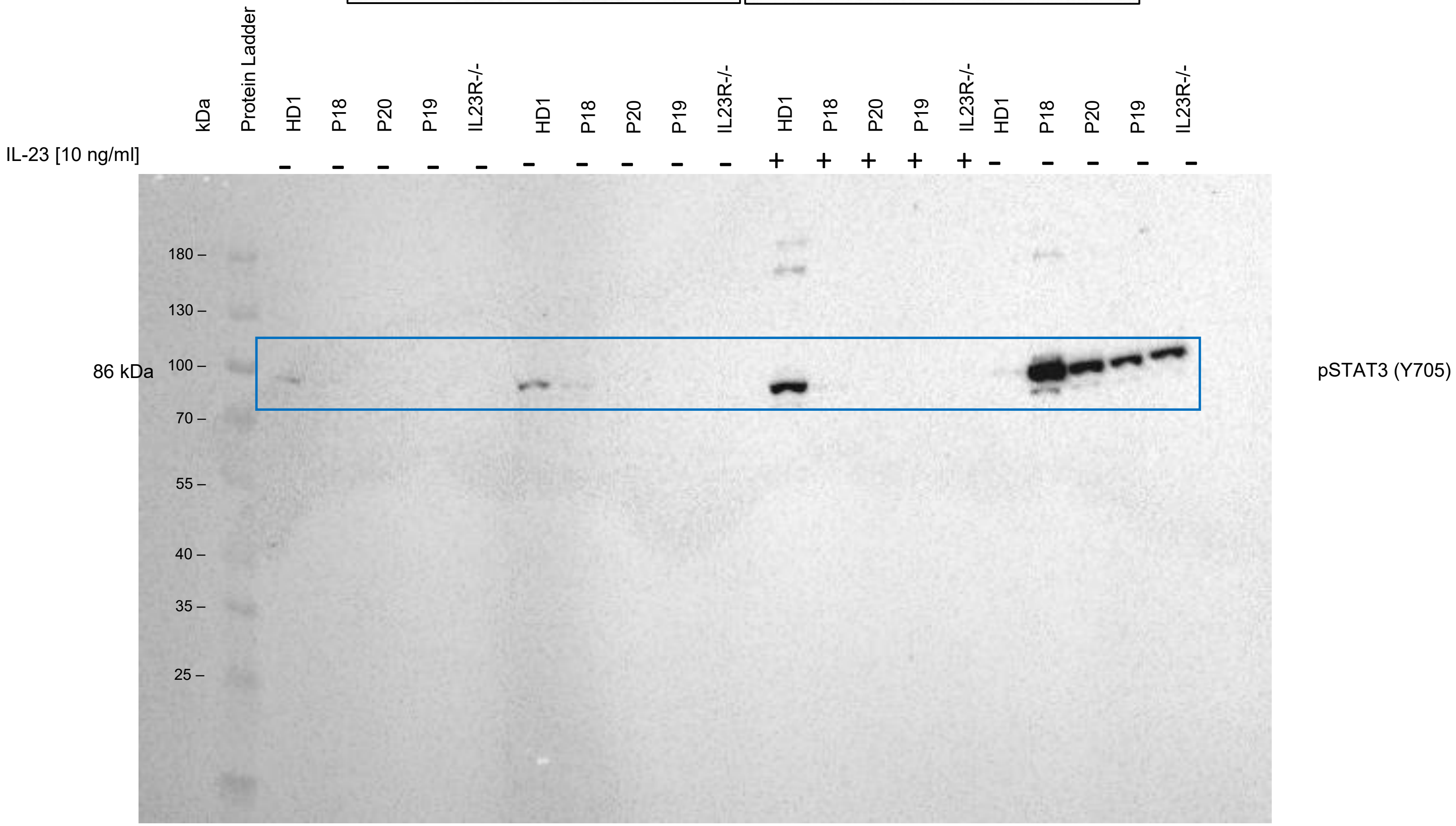


**The ladder image was merged with the original image from the figure to visualize protein size**

**SourceDataFS2**

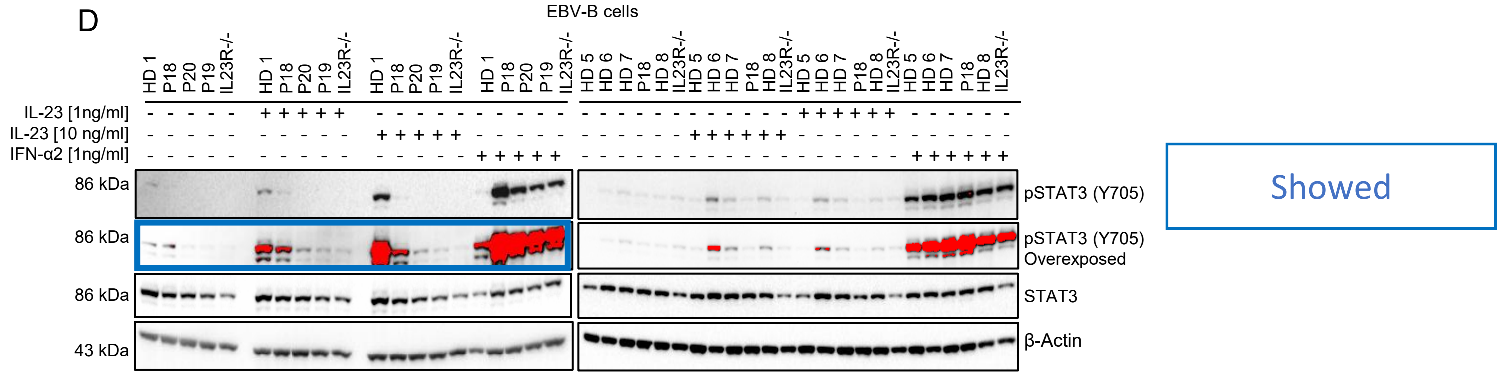


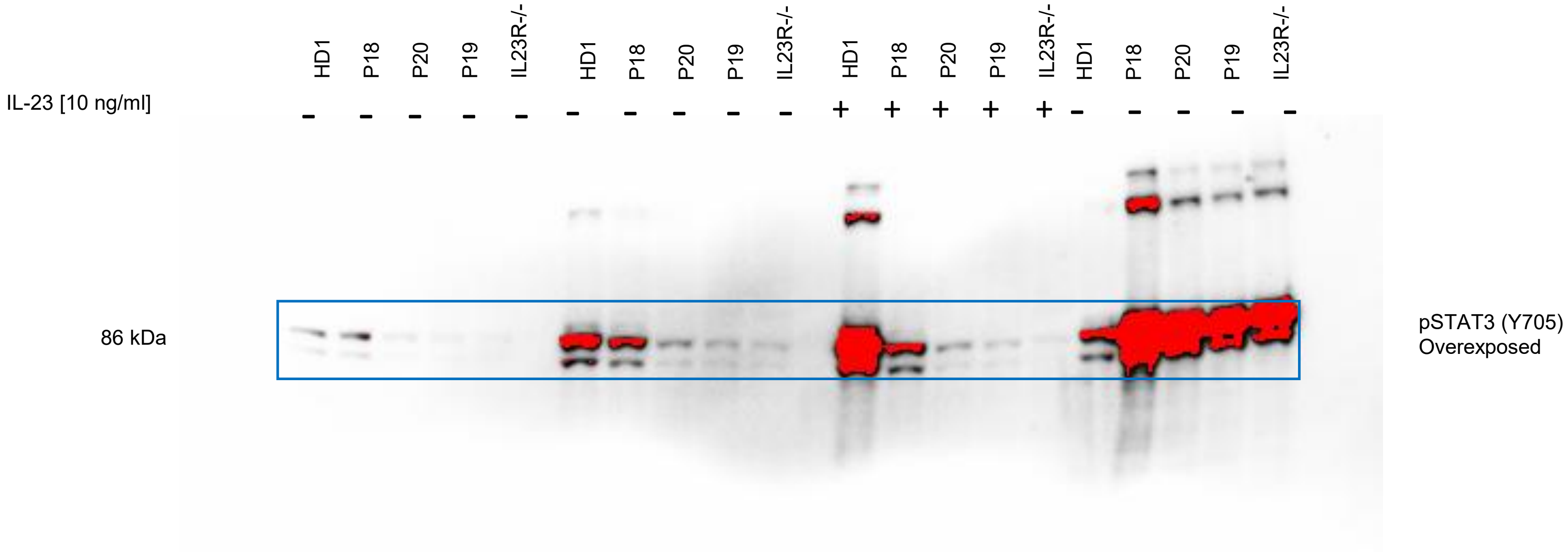

SourceDataFS2

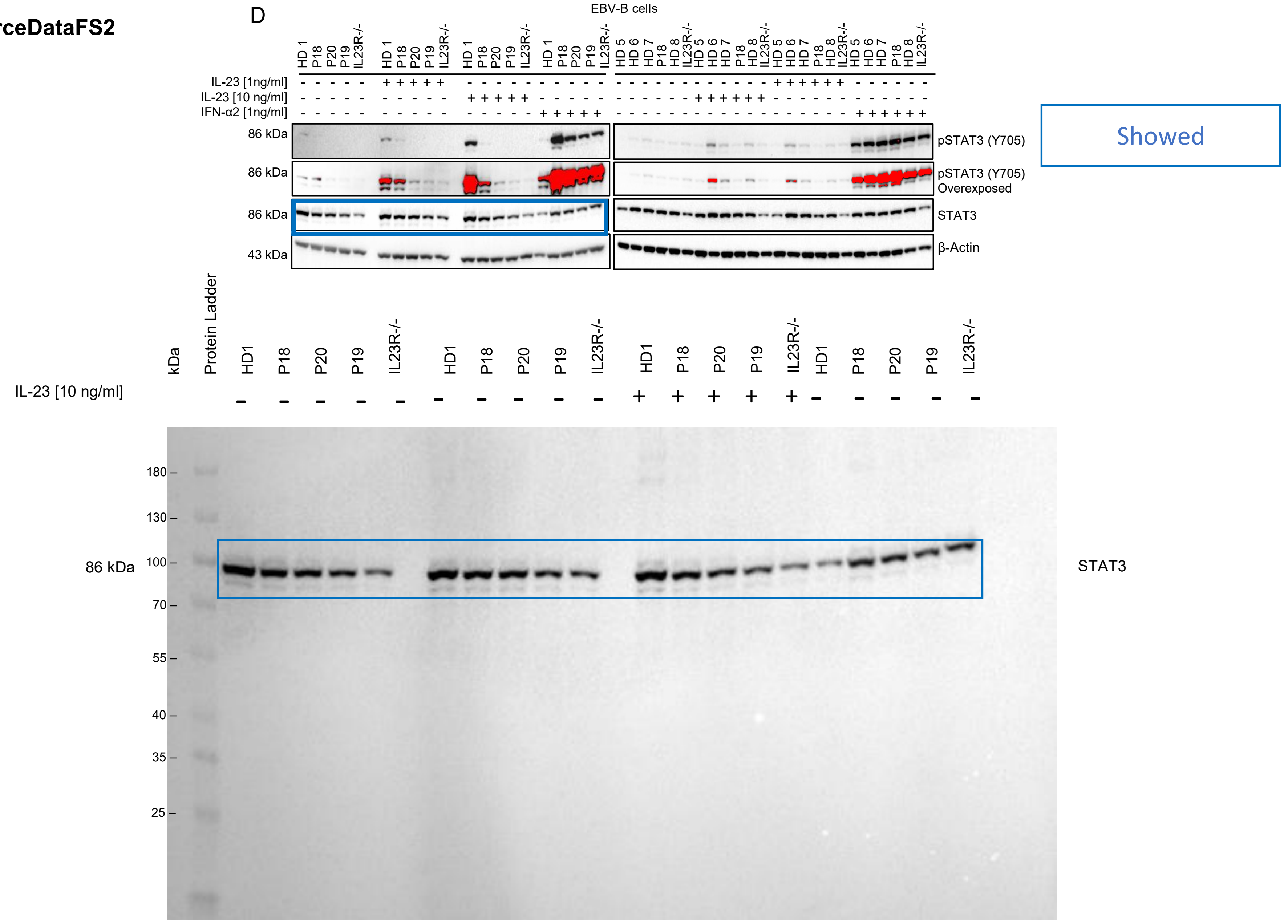


**The ladder image was merged with the original image from the figure to visualize protein size**

**SourceDataFS2**

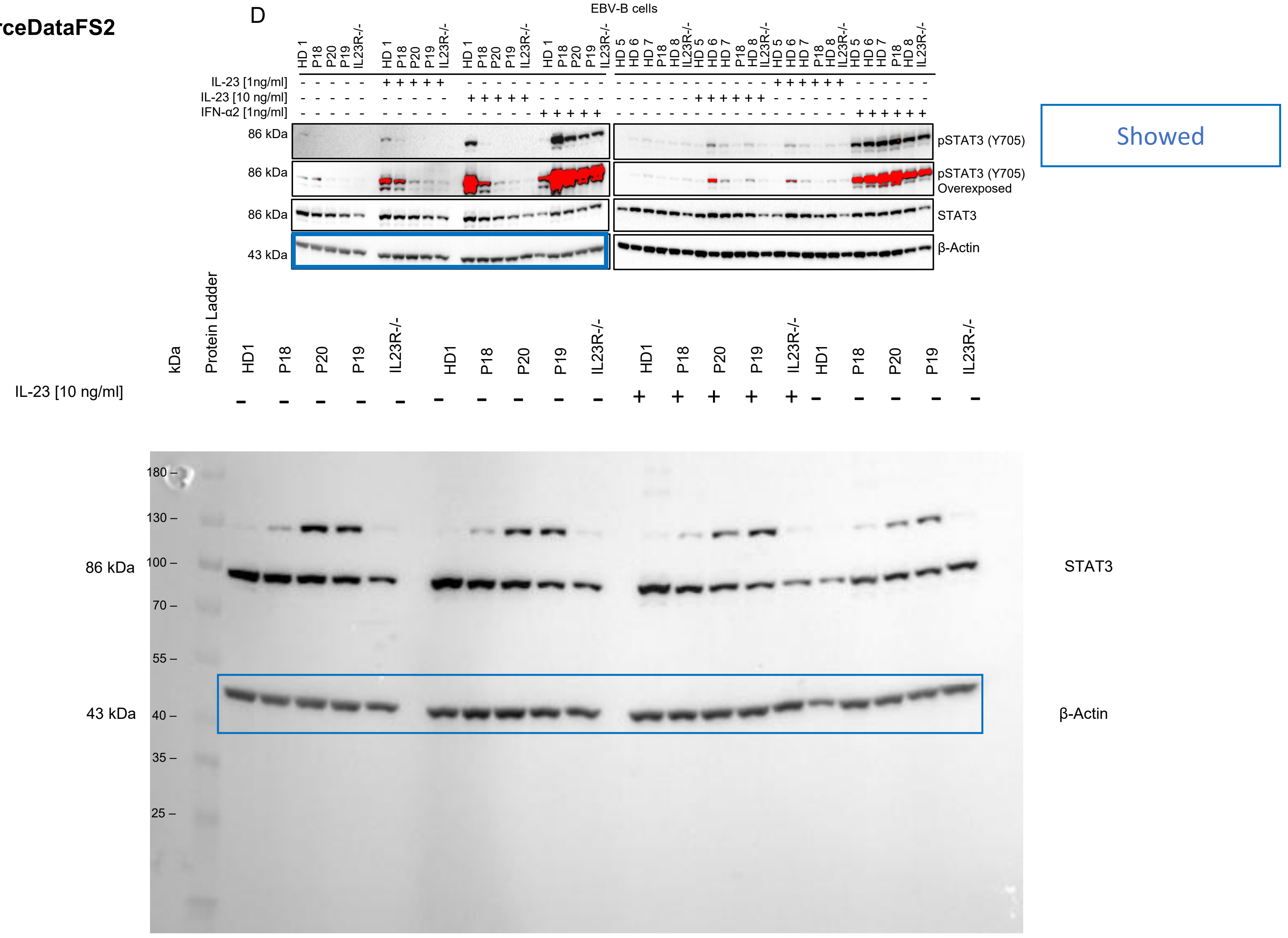


**The ladder image was merged with the original image from the figure to visualize protein size**

**SourceDataFS2**

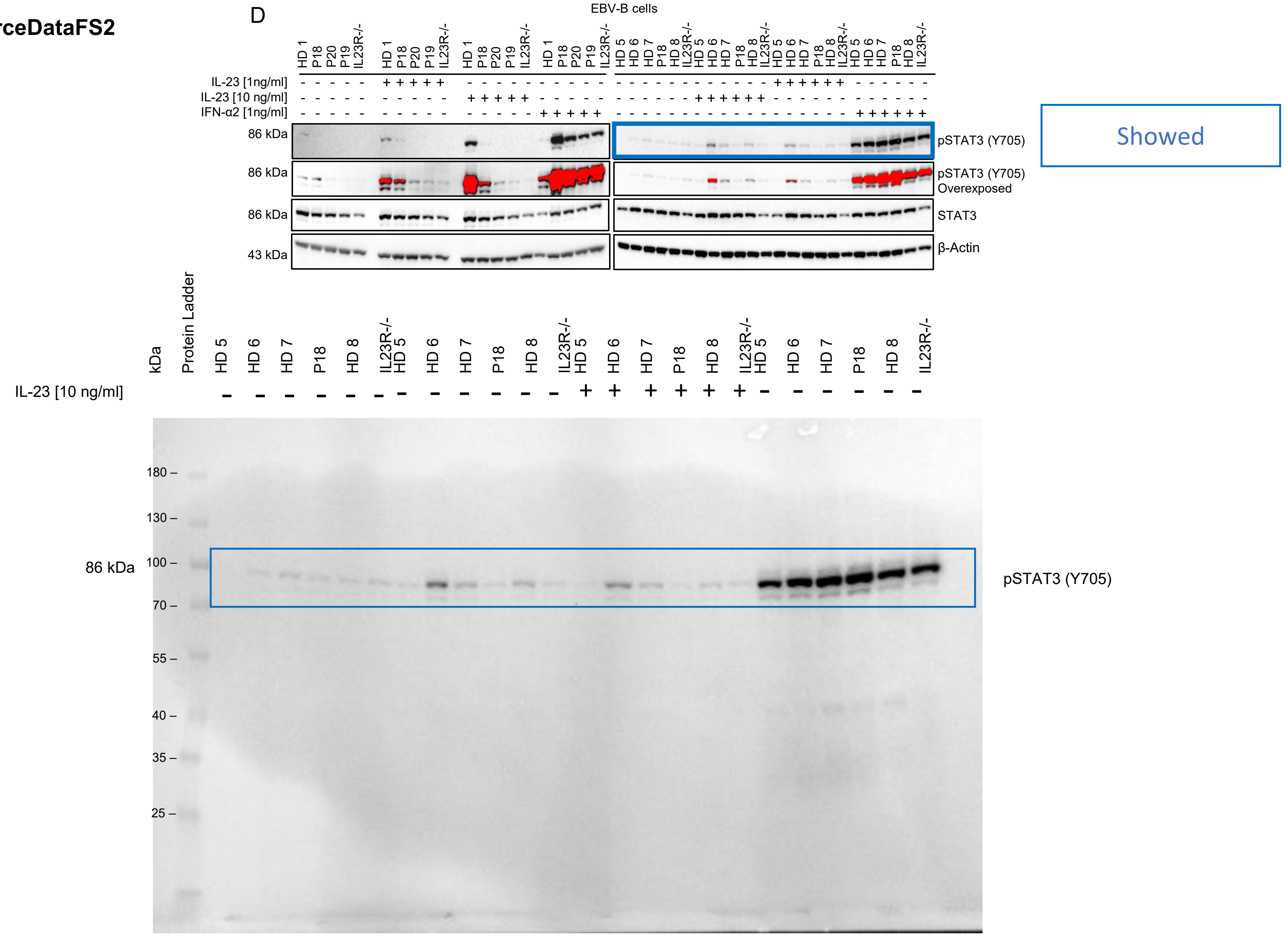


**The ladder image was merged with the original image from the figure to visualize protein size**

# SourceDataFS2

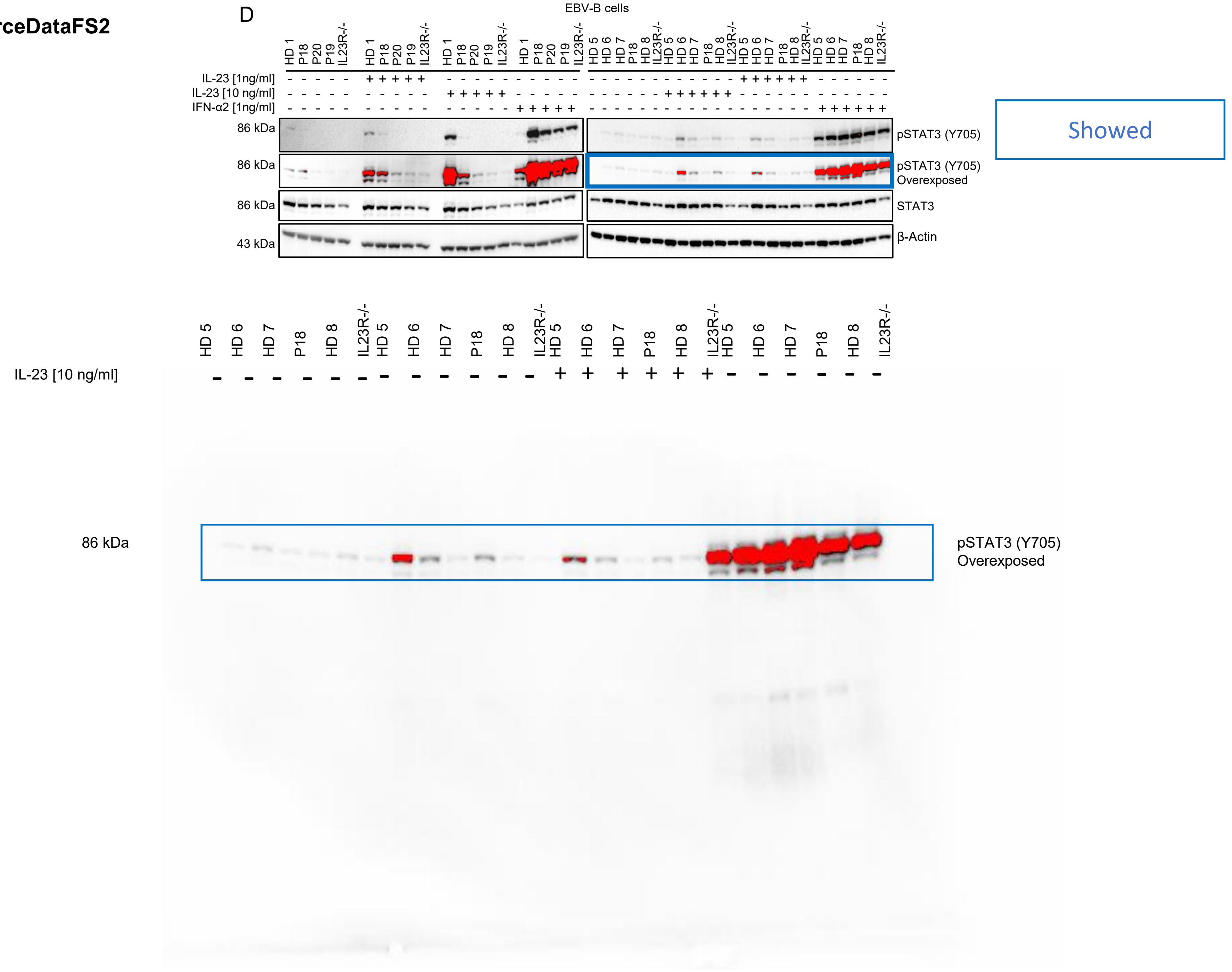

SourceDataFS2

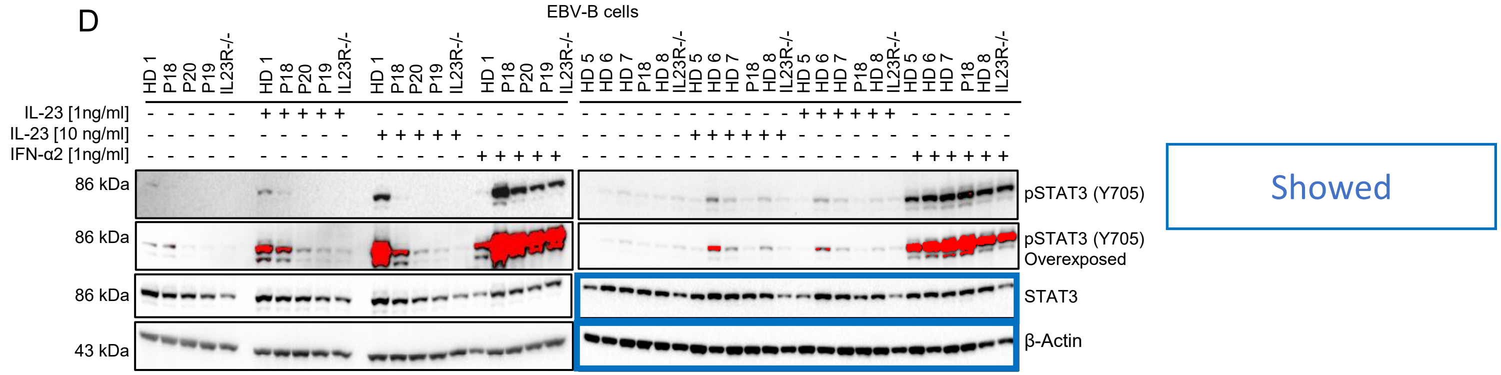


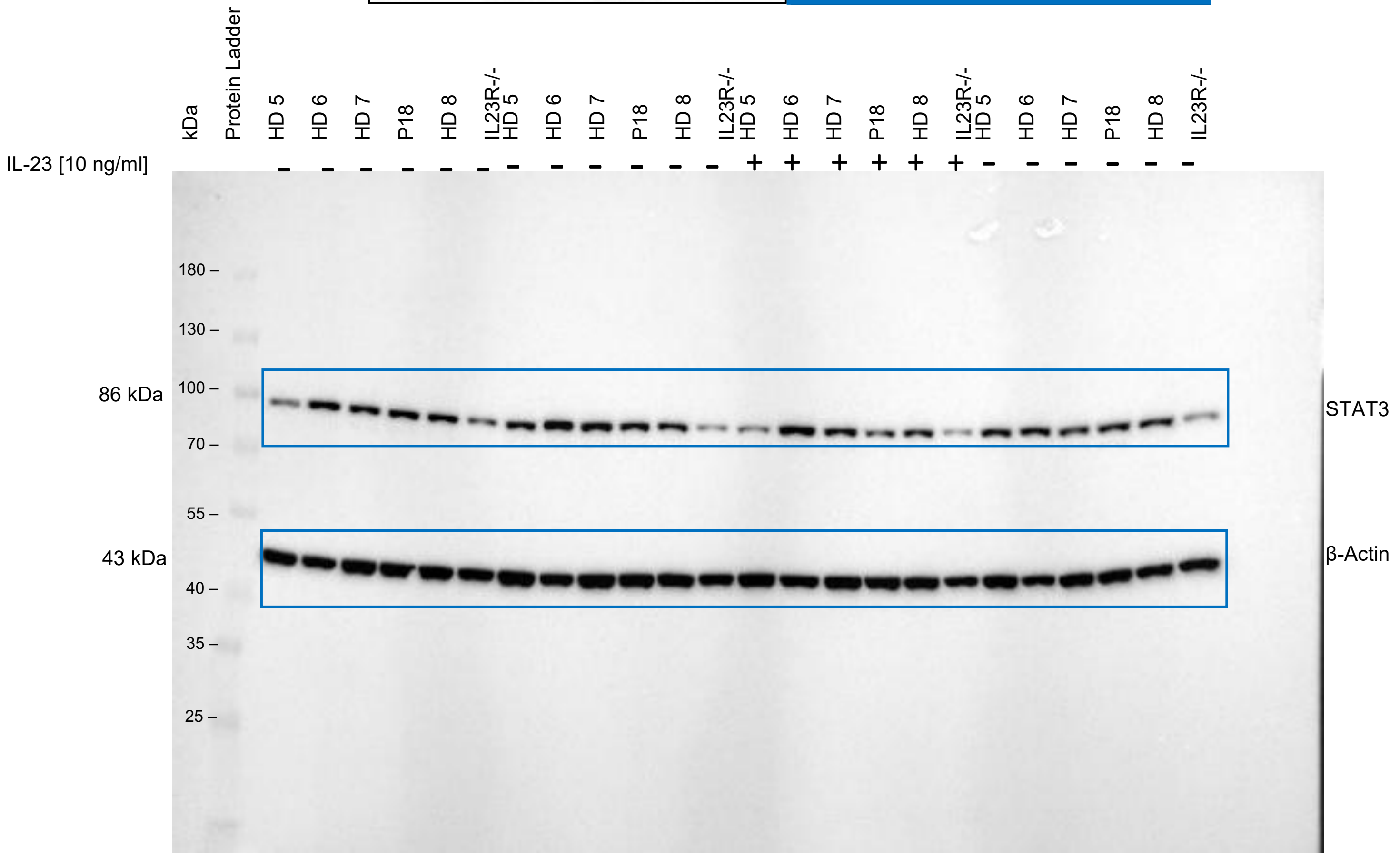


**The ladder image was merged with the original image from the figure to visualize protein size**

**SourceDataFS2**

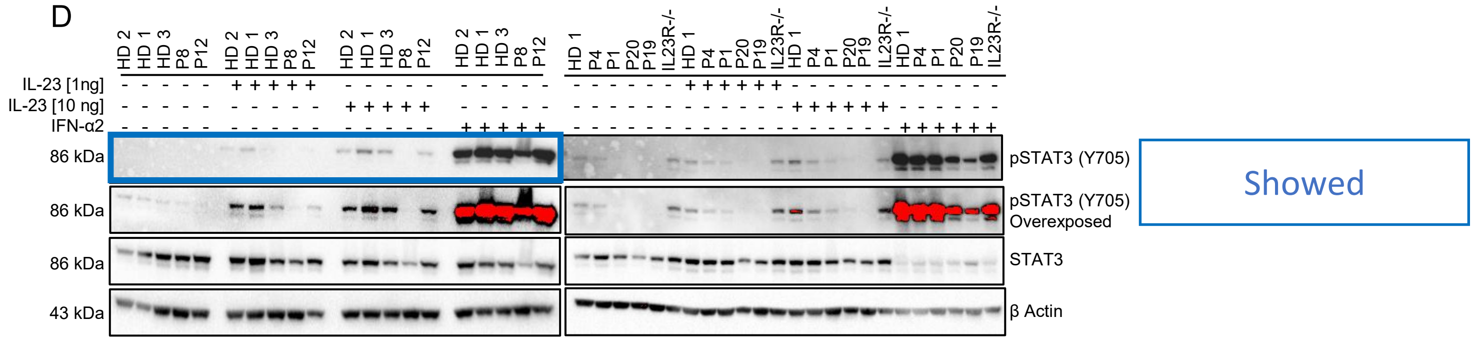


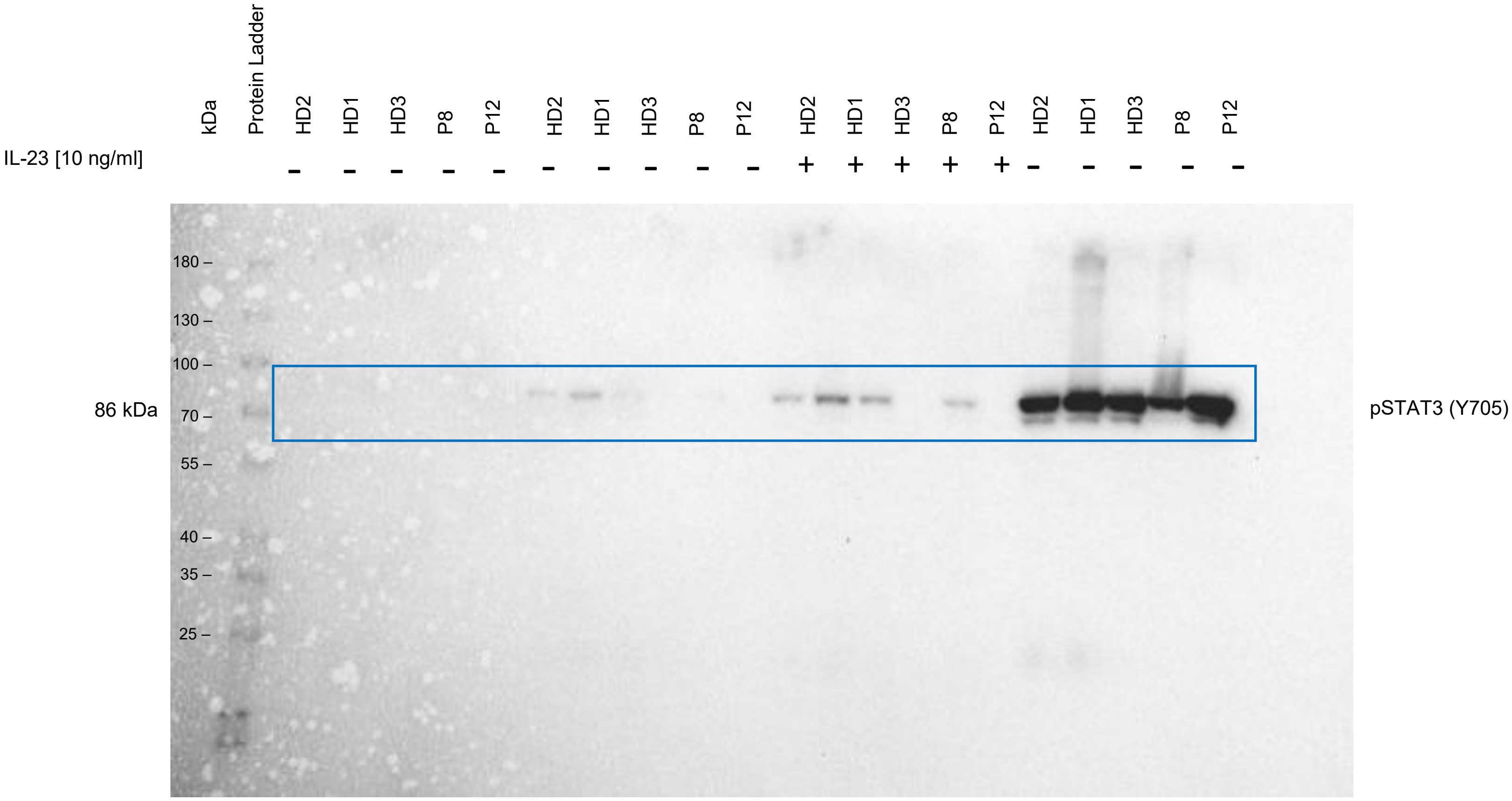


**The ladder image was merged with the original image from the figure to visualize protein size**

**SourceDataFS2**

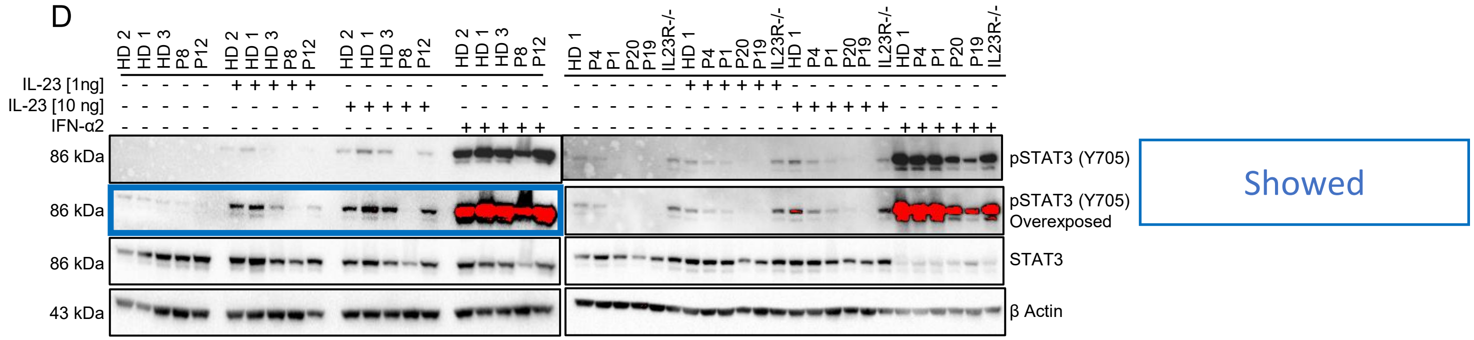


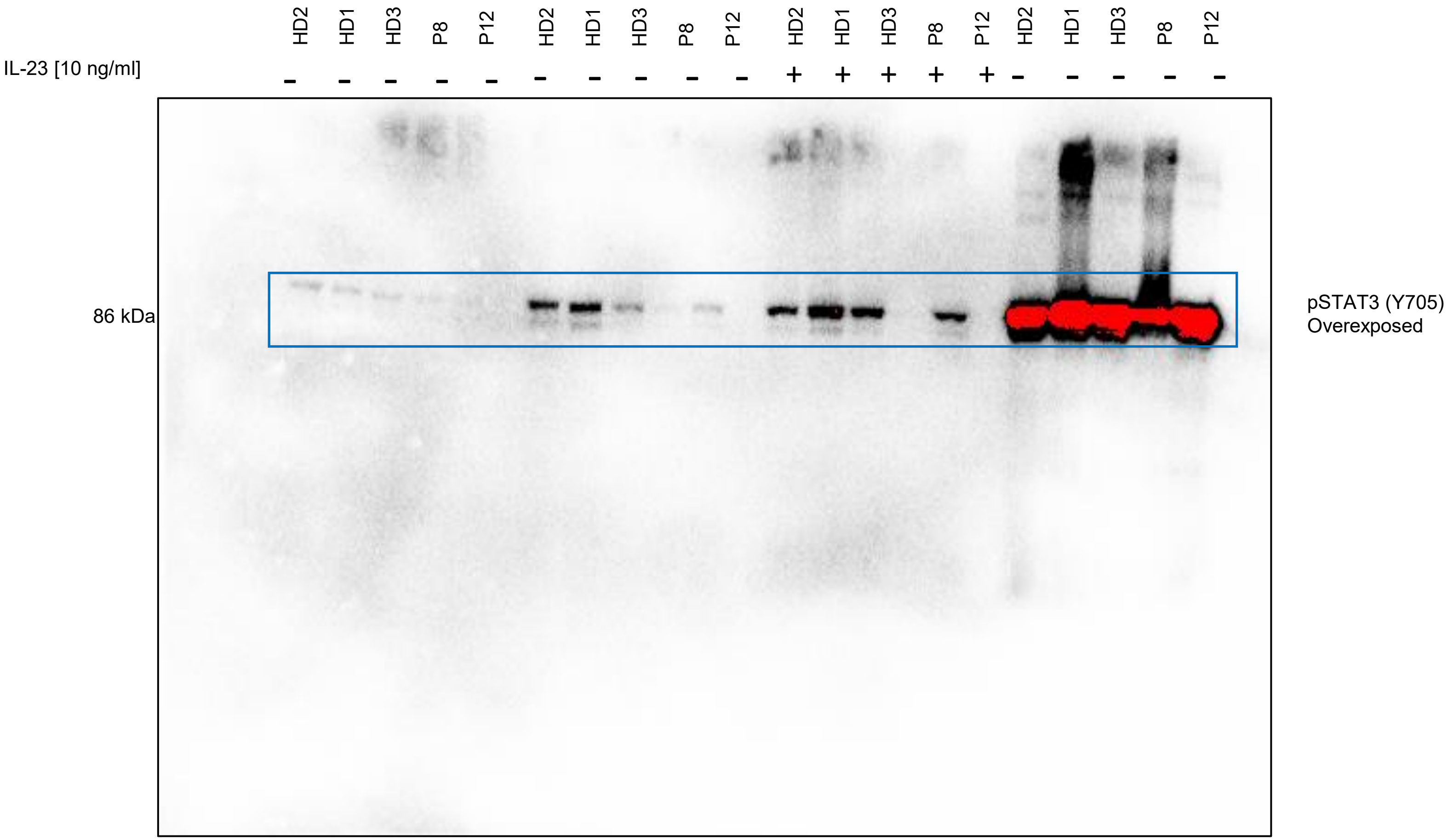

**SourceDataFS2**

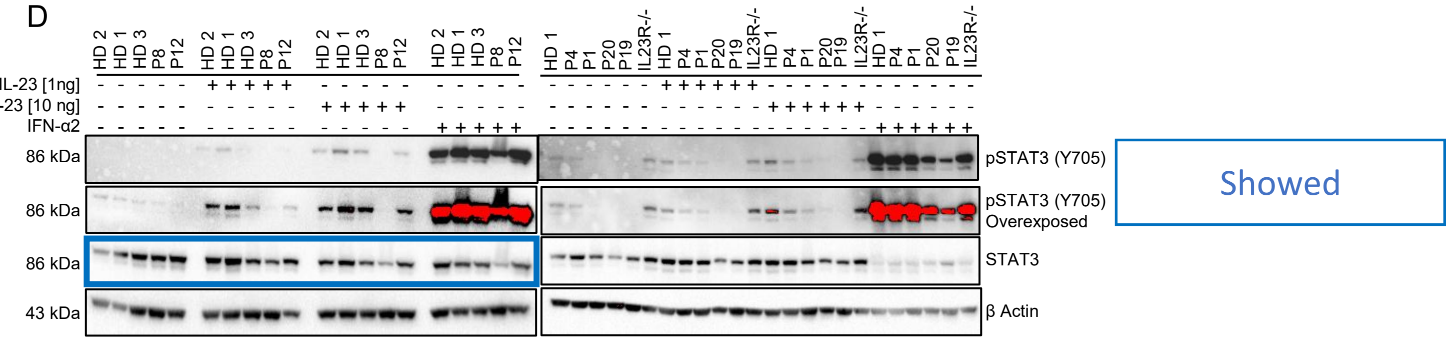


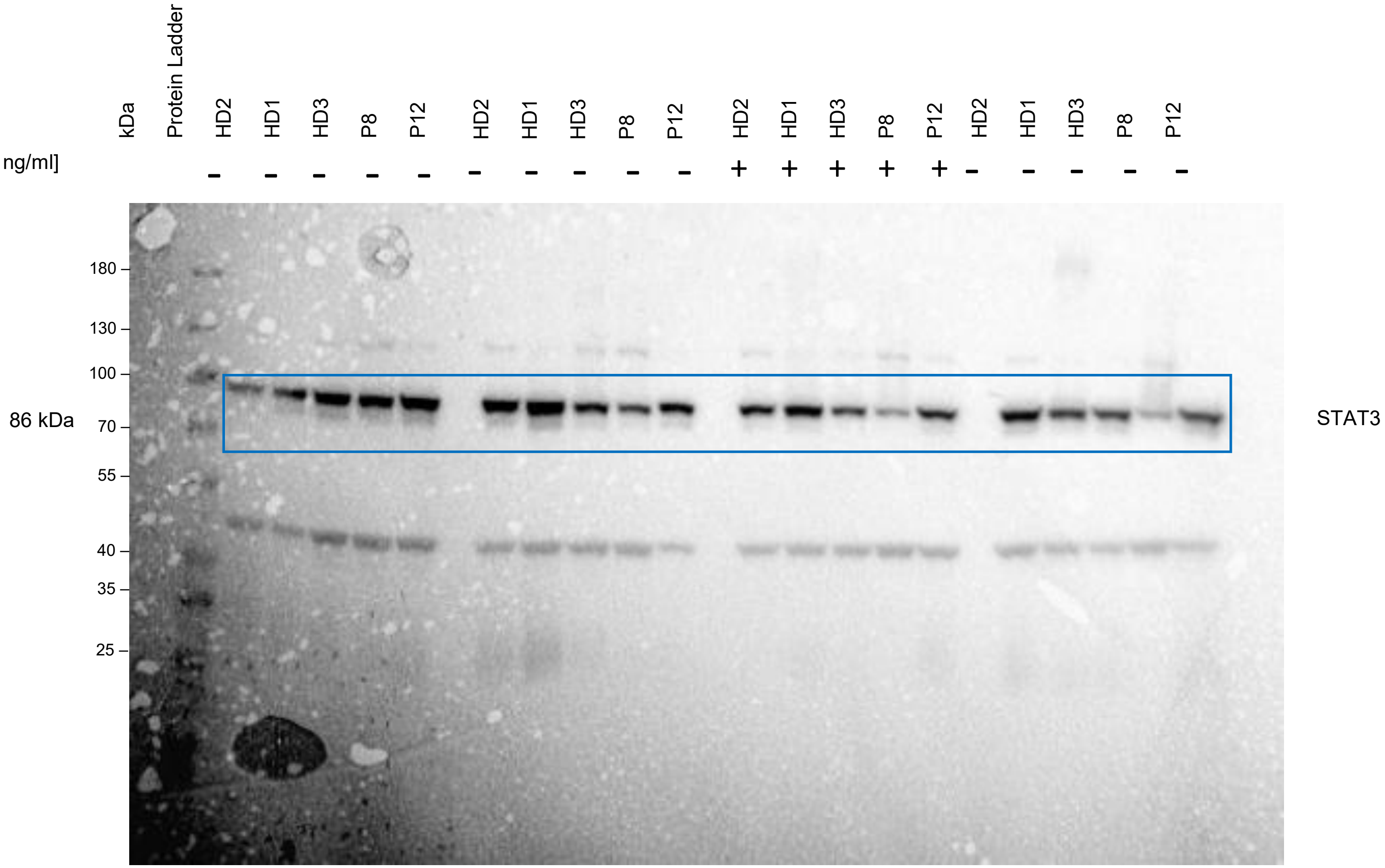


**The ladder image was merged with the original image from the figure to visualize protein size**

**SourceDataFS2**

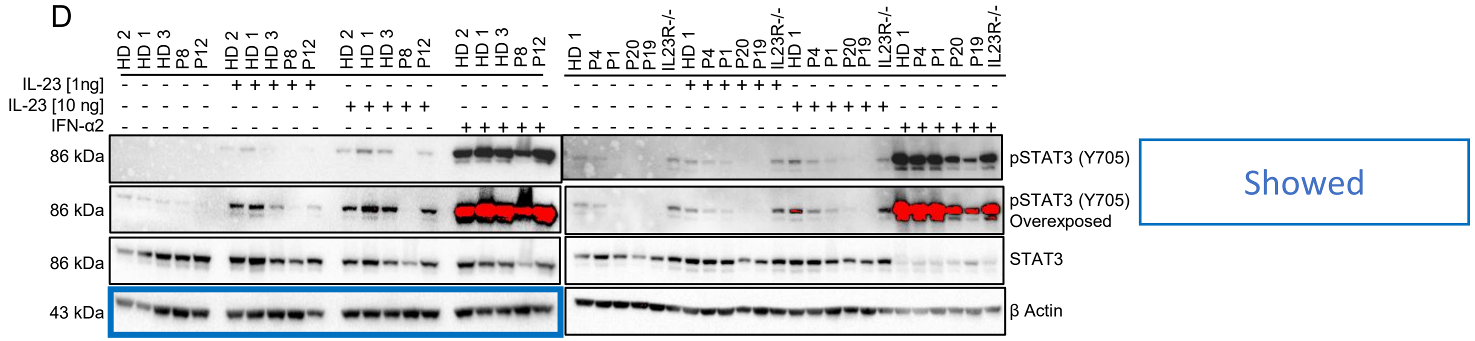


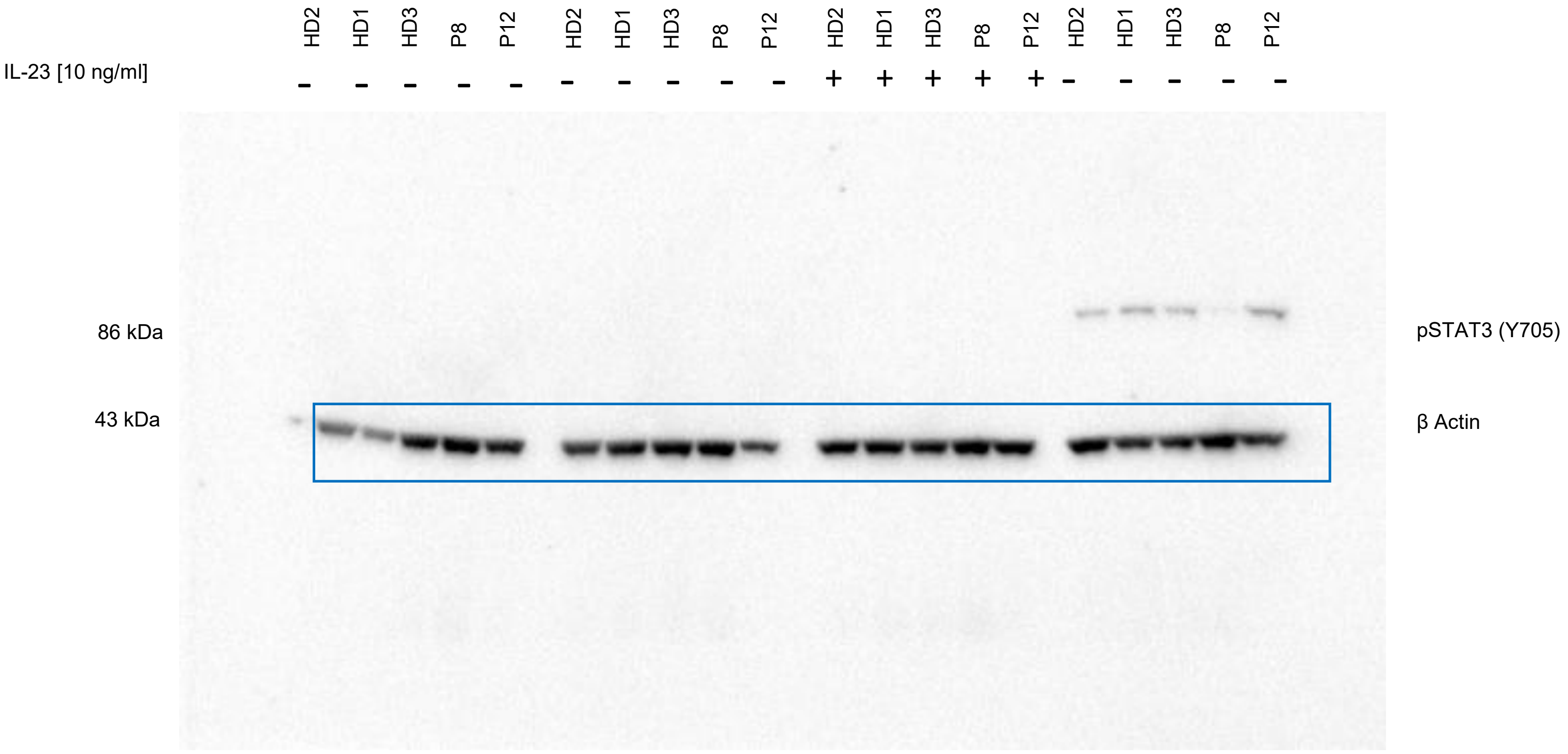

**SourceDataFS2**

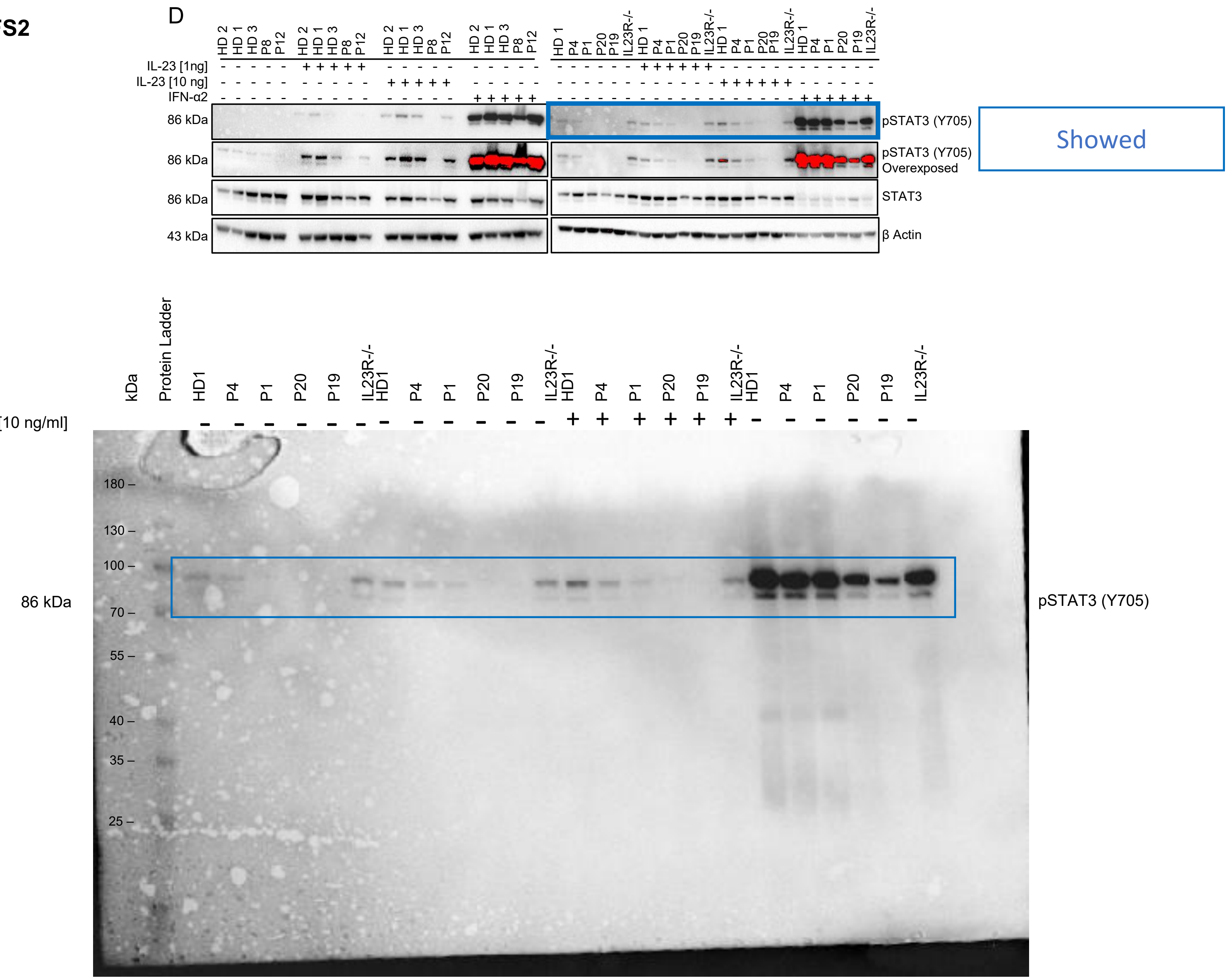


**The ladder image was merged with the original image from the figure to visualize protein size**

# SourceDataFS2

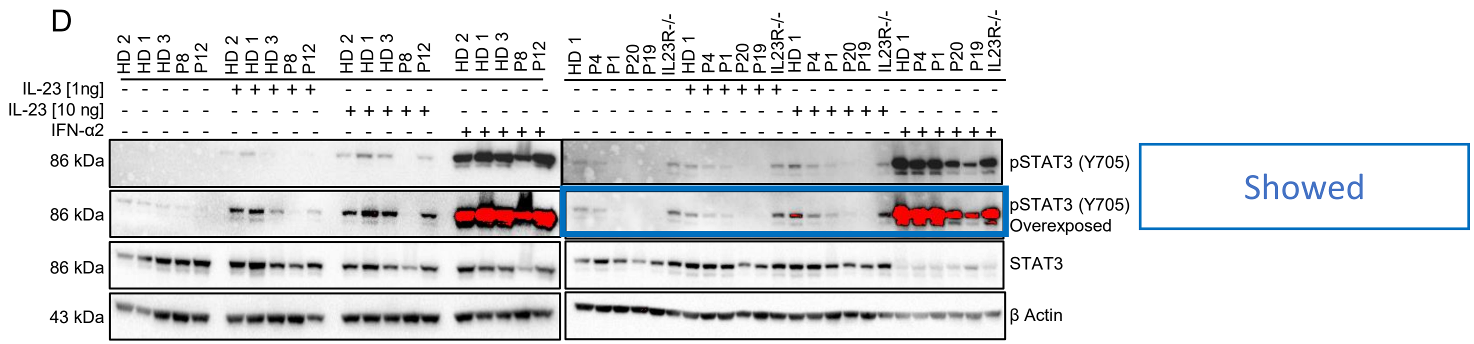


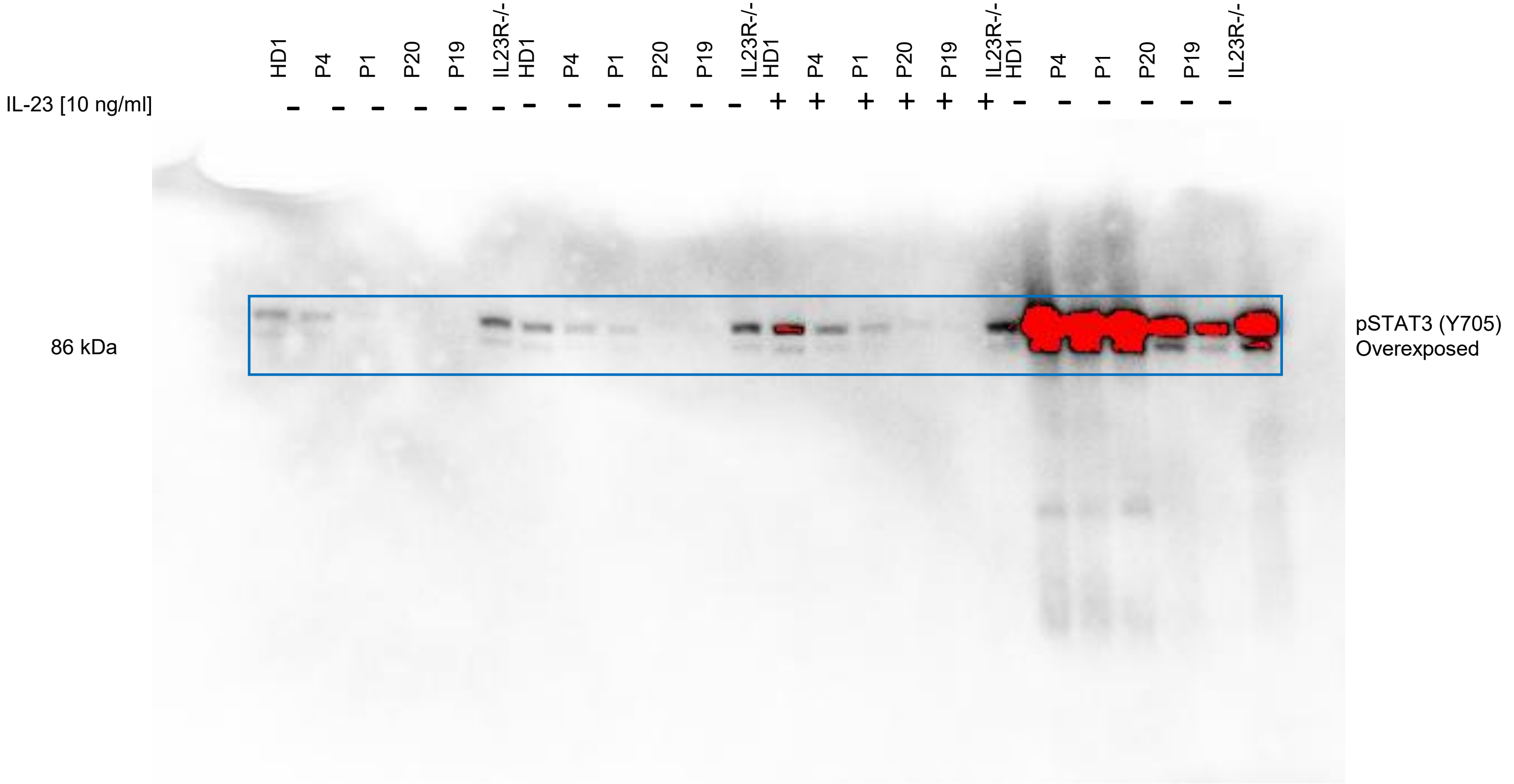

**SourseDataFS2**

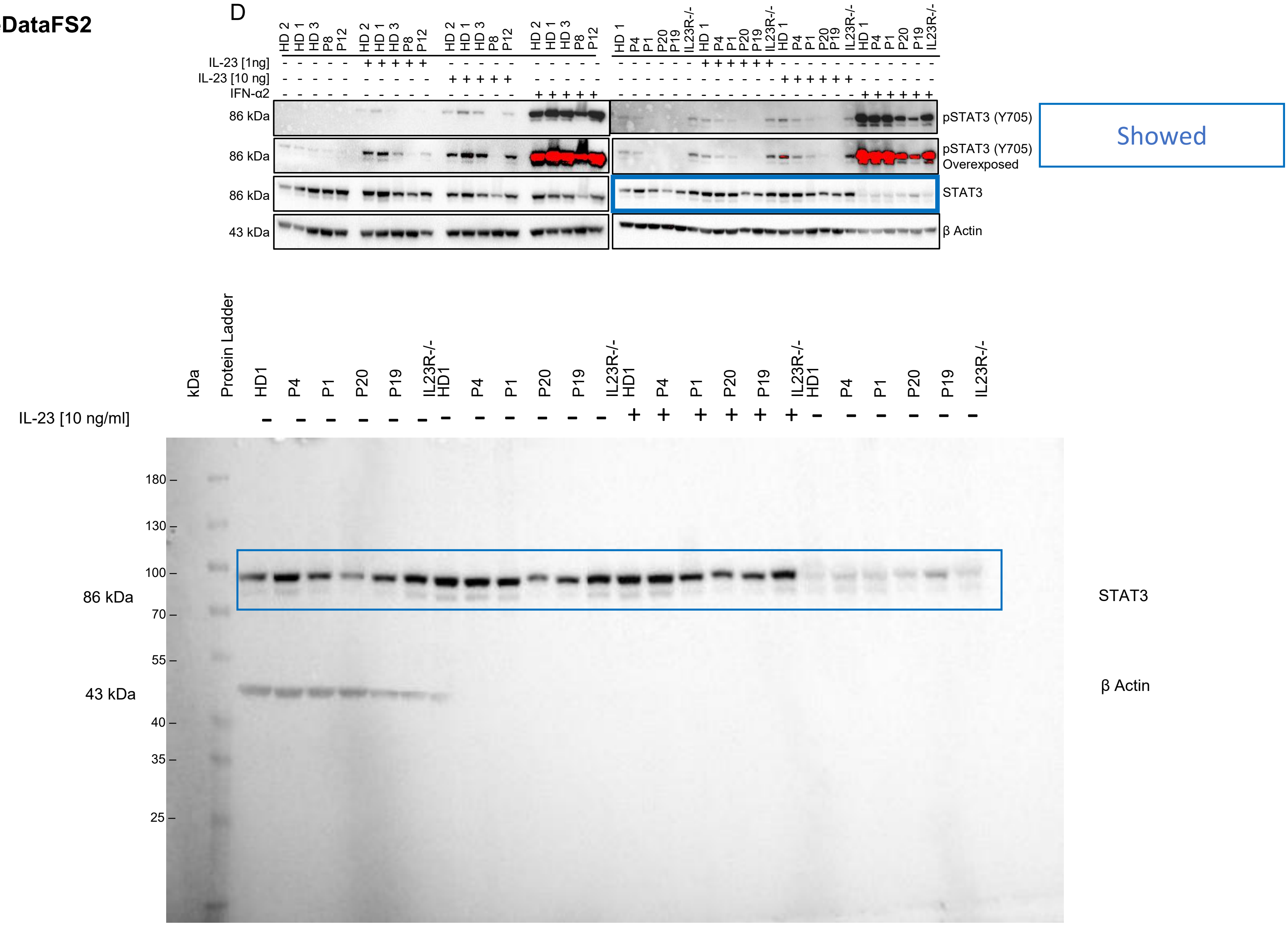


**The ladder image was merged with the original image from the figure to visualize protein size**

**SourceDataFS2**

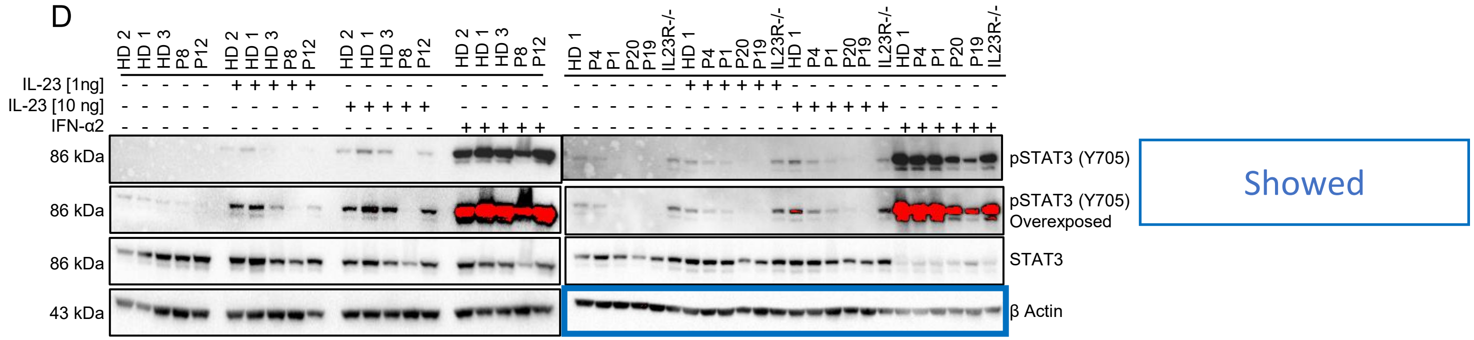


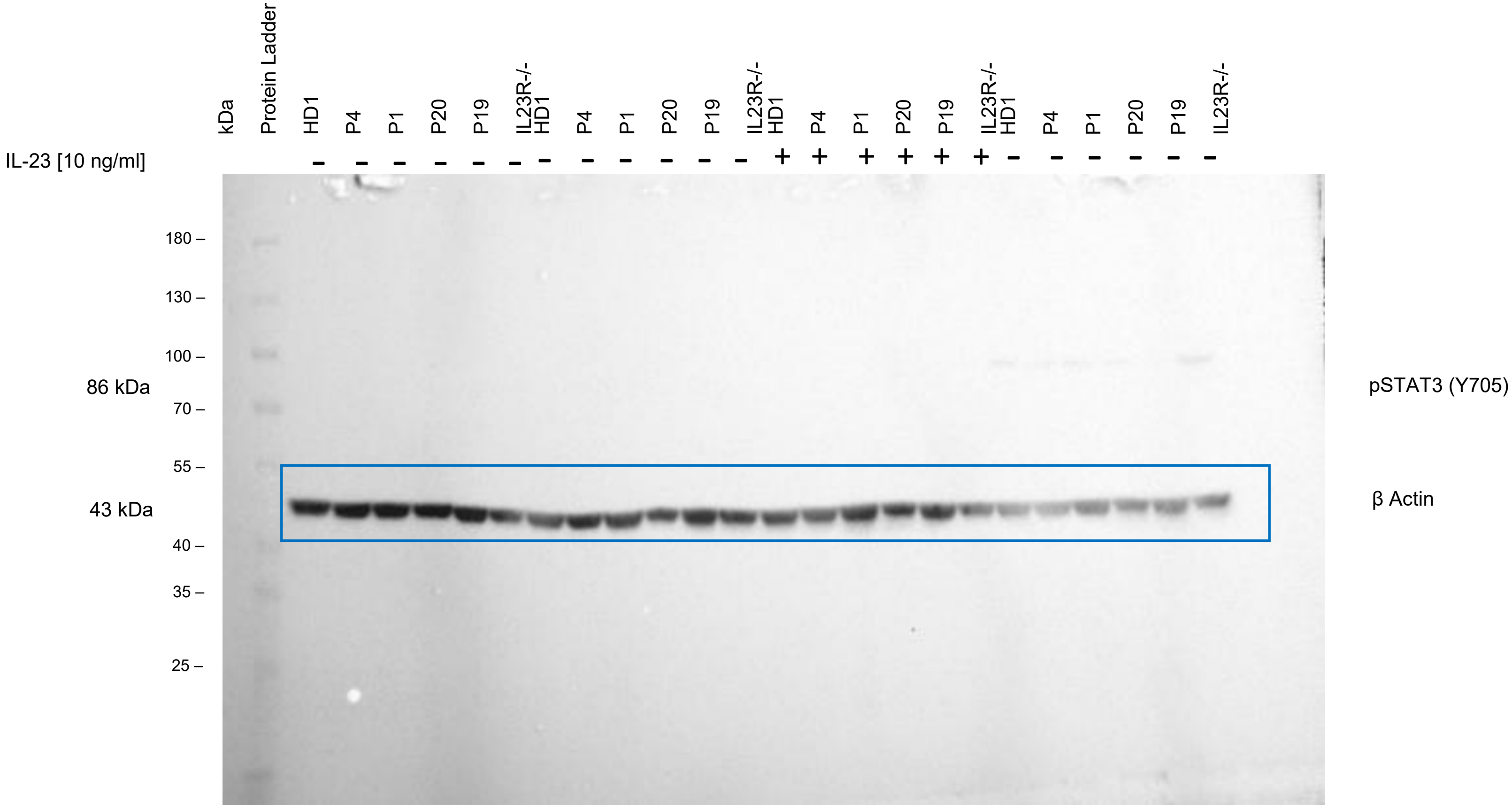


**The ladder image was merged with the original image from the figure to visualize protein size**
